# Structural variants and modifications of hammerhead ribozymes targeting influenza A virus conserved structural motifs

**DOI:** 10.1016/j.omtn.2022.05.035

**Published:** 2022-05-31

**Authors:** Tomasz Czapik, Julita Piasecka, Ryszard Kierzek, Elzbieta Kierzek

**Affiliations:** 1 Institute of Bioorganic Chemistry, Polish Academy of Sciences, Noskowskiego 12/14, 61-704 Poznan, Poland

**Keywords:** MT: oligonucleotides: therapies and applications, hammerhead ribozyme, influenza virus, RNA secondary structure, RNA conserved structural motifs, modified nucleosides, fluoroarabinonucleosides, shRNA

## Abstract

The naturally occurring structure and biological functions of RNA are correlated, which includes hammerhead ribozymes. We proposed new variants of hammerhead ribozymes targeting conserved structural motifs of segment 5 of influenza A virus (IAV) (+)RNA. The variants carry structural and chemical modifications aiming to improve the RNA cleavage activity of ribozymes. We introduced an additional hairpin motif and attempted to select ribozyme-target pairs with sequence features that enable the potential formation of the *trans*-Hoogsteen interactions that are present in full-length, highly active hammerhead ribozymes. We placed structurally defined guanosine analogs into the ribozyme catalytic core. Herein, the significantly improved synthesis of 2′-deoxy-2′-fluoroarabinoguanosine derivatives is described. The most potent hammerhead ribozymes were applied to chimeric short hairpin RNA (shRNA)-ribozyme plasmid constructs to improve the antiviral activity of the two components. The modified hammerhead ribozymes showed moderate cleavage activity. Treatment of IAV-infected Madin-Darby canine kidney (MDCK) cells with the plasmid constructs resulted in significant inhibition of virus replication. Real-time PCR analysis revealed a significant (80%–88%) reduction in viral RNA when plasmids carriers were used. A focus formation assay (FFA) for chimeric plasmids showed inhibition of virus replication by 1.6–1.7 log_10_ units, whereas the use of plasmids carrying ribozymes or shRNAs alone resulted in lower inhibition.

## Graphical abstract

**Figure:**
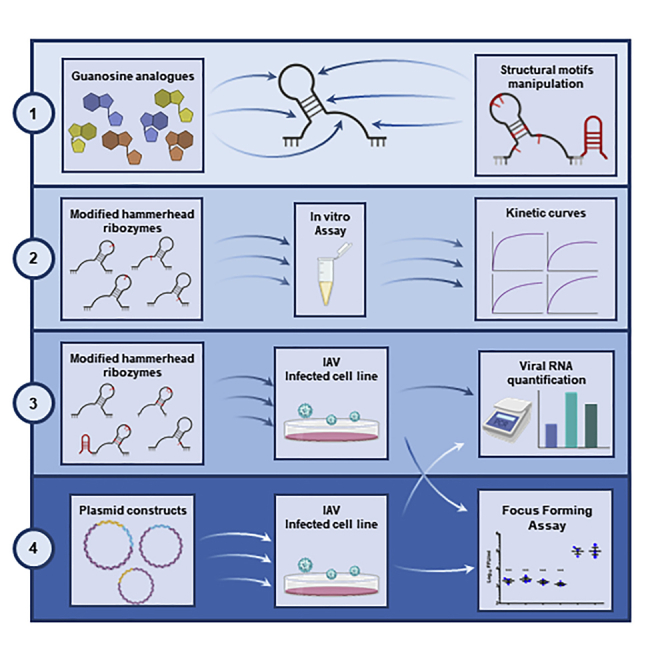

## Teaser

As a result of *in vitro* and *in cellulo* tests, the authors propose a new insight into structural modification of the hammerhead ribozyme. In this study, the authors introduced several chemical and structural modifications to the hammerhead ribozyme. Modified ribozymes were designed to target RNA of the IAV.

## Introduction

**Figure 1:**
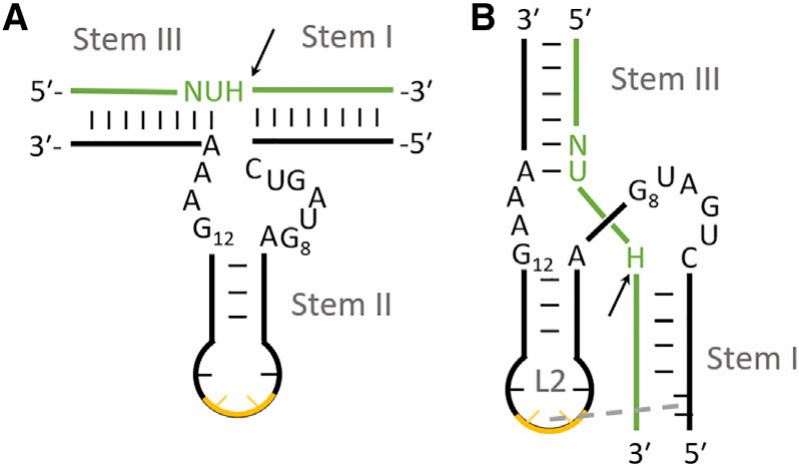
Hammerhead ribozyme type II scheme (A) Model hammerhead ribozyme secondary structure. (B) Possible tertiary interactions between loop L2 and stem I are enabled in the Y conformation. The target sequence is marked in green, with the black arrow indicating the cleavage site. Two nucleosides, 2.2 and 2.3, of tetraloop L2 are marked in yellow. Only the sequence of the conserved catalytic core and target NUH are shown.

Hammerhead ribozymes are short RNA sequences with well-characterized catalytic properties. The smallest variant consists of a 22-nt conserved catalytic core capable of cleaving RNA molecules and two arms with variable sequences complementary to the regions adjacent to the target RNA cleavage site.^1^ A primary requirement for the hammerhead ribozyme target site is the presence of the NUH sequence (N, any nucleotide; U, uridine; H, any nucleotide except guanosine) (Figure 1). Among the frequently highlighted advantages of using ribozymes are their high specificity for their target sequence and the ability of a single ribozyme to cleave multiple target RNA molecules. The latter feature is exceptional for *trans*-acting ribozymes and do not refer to natural *cis*-acting ribozymes. The limitations of this strategy are also known and include effective cellular delivery and stability. One of the main factors regulating the high catalytic activity of ribozymes is the dependence on the concentration of magnesium ions, as its concentration in cells is much lower than the optimal concentration for *in vitro* RNA cleavage. Attempting to adjust ribozyme catalytic activity to the conditions present in cells results in the introduction of a number of changes to the catalytic core, chemical modifications, and the addition of elements stabilizing the active conformation of the ribozyme-substrate complex.^2^, ^3^, ^4^, ^5^, ^6^, ^7^ To date, reports have shown that standard variants of hammerhead ribozymes have the ability to inhibit viral proliferation, including that of influenza A virus (IAV).^8^, ^9^, ^10^, ^11^, ^12^, ^13^, ^14^ Hammerhead ribozymes have been the subject of clinical trials demonstrating their effective delivery and applications as a therapeutic for human immunodeficiency virus (HIV).^9^^,^^15^ In addition, new variants of catalytic nucleic acids have been used against HIV with success.^16^

In this study, we present a few potentially favorable structural variants and modifications of *trans*-acting hammerhead ribozymes that should exhibit multiple turnover kinetics. We also tested these ribozymes against the IAV in infected cell culture. One of these strategies is based on an attempt to reconstruct the tertiary interactions present in full-length natural hammerhead ribozymes, the catalytic activity of which significantly exceeds the minimum variant (Figure 1).^3^^,^^17^, ^18^, ^19^, ^20^ This can potentially be obtained by selecting a target sequence containing a U residue at the position 7 nt downstream of the cleavage site to form a *trans*-Hoogsteen base pair with the A residue in the GUGA tetraloop of the conserved ribozyme core. In previous studies, this interaction was identified to be one of the requirements that a sequence must fulfill, in addition to conservation of the ribozyme CUGA tetraloop, to improve the catalytic activity of the ribozyme.^3^ Another variant contains an additional hairpin motif introduced into the hammerhead ribozyme structure, which may also contribute to ribozyme conformation stabilization.^4^ The 19-nt hairpin, also called the tetraloop receptor (TLR), is placed upstream of the 5′ ribozyme arm, which enables the formation of tertiary interactions with the tetraloop of the ribozyme catalytic core (Figure 1). Two guanosine residues in the catalytic core are potentially involved in transesterification catalysis. These residues (G8 and G12) connect with the RNA strand that undergoes cleavage. N1 of the G12 residue interacts with O2ʹ of the target RNA in the cleavage site, resulting in activation of the 2ʹ-hydroxyl to act as a nucleophile in an S_N_2 reaction, forming the 2ʹ,3ʹ-cyclic phosphate terminus. The G8 2ʹ-hydroxyl stabilizes O5ʹ of the leaving group in a cleavage reaction, resulting in the creation of a new 5ʹ end.^21^^,^^22^ It is clear that the conformations of these two guanosine residues are crucial for the hammerhead transesterification reaction rate. In the present study, we applied conformationally defined nucleosides to modify the hammerhead catalytic core to influence ribose puckering and the *syn-anti* conformation by introducing guanosine modifications at the G8 and G12 positions (Figure S2). These chemical modifications were intended to change the ribozyme conformation and modulate its reactivity. Finally, we applied the most potent hammerhead ribozymes to chimeric short hairpin RNA (shRNA)-ribozyme plasmid constructs to improve the antiviral activity of the two components.^23^^,^^24^ All hammerhead ribozymes tested in this study were designed to target conserved motifs of IAV segment 5 (+)RNA. Notably, the target sites were selected on the basis of the previously published structure of segment 5 (+)RNA ((+)RNA5) of the A/California/04/2009 (H1N1) strain and available data on structure-guided viral inhibition (Figure S1).^25^^,^^26^ Moreover, these sequences fulfill the known requirements for potential structural stabilization and were determined to be effective targets for nucleic-acid-based inhibitory approaches.

The obtained results show unexpected adverse effects caused by the modifications and structural variants of the hammerheads. The analyses also indicate the critical role of target-site selection for effective inhibition of the virus. Moreover, we demonstrate significant inhibition of influenza virus replication by applying the plasmid constructs. This approach, based on effective delivery of the inhibitory molecules and considering the target structural context, may lead to the development of constructs resulting in substantial antiviral effects.

## Results

### Improved chemical synthesis of 2′-deoxy-2′- fluoropurine arabinoside derivatives

Herein, a new approach to synthesize purine derivatives of 2′-deoxy-2′-fluoroarabinonucleotides via the substitution of 2′-O-trifluoromethanesulfonyl with triethylammonium fluoride without the use of bulky protecting groups at the 5′ and 3′ positions is presented (Scheme S1).

As described earlier, the synthesis of 2′-deoxy-2′-fluoroarabinonucleosides is a laborious process with low yield of the final products because the incorporation of fluoride at the β-C2′ position requires simultaneous protection of the 5′ and 3′ hydroxyls with bulky protecting groups.^27^ This approach is necessary when the bulky, protected derivative is directly fluorinated with (diethylamino) sulfur trifluoride (DAST)^28^, ^29^, ^30^ but also when the 2′-O-triflate derivative is substituted with tetrabutylammonium fluoride (TBAF).^31^ Both approaches face two significant difficulties. The first is the low-yield synthesis of 5′,3′-diprotected nucleosides. The second is linked to the considerable contribution of the side reaction product accompanying DAST and TBAF treatment, which is related to the equilibrium of the *C2′-endo/C3′-endo* conformation of ribonucleotides.

To avoid such a laborious and low-yield synthesis, N^2^-(dimethylaminomethylene)-guanosine^32^ was protected with tetraisopropyldisiloxane (TIPDSi) (Markiewicz protecting group)^33^ followed by placement of a triflate at the C2′ position. Deprotection of the last derivative of TIPDSi with triethylammonium fluoride (TEAHF) resulted in the formation of a 2′-O-triflate derivative (^19^F nuclear magnetic resonance [NMR] δ −74.86 ppm; ^1^H NMR δ 5.96 ppm [d, H-1′, J_H1′-H2′_ = 4.75 Hz]), which spontaneously and completely became inverted in the 3′-O-triflate derivative (^19^F NMR δ −77.75 ppm; ^1^H NMR δ 5.89 ppm [d, H-1′, J_H1′-H2′_ = 5.86 Hz]). To avoid 2′-O-triflate isomerization, deprotection of TIPDSi was performed with triethylammonium fluoride in the presence of acetic anhydride at room temperature for 2 h.^34^ The N-protected 5′,3′-diacetyl-2′-O-triflate guanosine in tetrahydrofuran (THF) solution was treated in a plastic vessel with 7 equivalents of a 1 molar solution of TEAHF^35^ in pyridine for 60–65 h at 37°C. ^19^F NMR analysis of the reaction mixture indicated the appearance of a new signal at −197 ppm, and the peak at −74 ppm shifted to −77 ppm. The signal at −197 ppm indicates the presence of a fluoride substituent at β-C2′, whereas the peak at −77 ppm is related to the appearance of the released triflate anion. Synthesis of the 8-bromoguanosine derivatives with bromine in water was performed at that stage of synthesis. Next, the reaction mixture was treated with aqueous ammonia in methanol, and the deprotected 2′-deoxy-2′-fluoroarabinonucleosides were converted into N2-dimethylaminomethylene derivatives using the same method as that used for the N protection of guanosine.^32^ The next step was the protection of the 5′-hydroxyl with a dimethoxytrityl group. Finally, the N-protected-5′-O-dimethoxytrityl guanosine derivatives were converted into the corresponding 3′-O-phosphoramidites according to a standard method.^36^, ^37^, ^38^

Using the above procedure, protected derivatives of 2′-deoxy-2′-fluoroarabinoguanosine, 2′-deoxy-2′-fluoro-8-bromoarabinoguanosine, 2′-deoxy-2′-fluoroarabinoadenosine, and 2′-deoxy-2′-fluoro-2,6-diaminopurine arabinoriboside were synthesized. The overall yields of the synthesis of the purine 2′-deoxy-2′-fluoroarabinoside derivatives were 40%–50%, which are 2- to 3-fold greater than that reported for 2′-deoxy-2′-fluoroarabinoguanosine.^27^^,^^39^ Because 2′-deoxy-2′-fluoroarabinonucleosides and their stability have been well described in the literature, we did not determine the stability of the synthesized analogs.^40^^,^^41^

### Selection of target sites

Selection of the target sites for ribozyme activity was based on an analysis of the published structural model.^25^ All RNA regions targeted in this paper are located in conserved RNA secondary structure motifs, which are preserved among distinct and distant influenza strains. They are also potentially functional in the influenza virus replication cycle and accessible for interaction with oligonucleotides, according to available experimental data (Figure S1).^25^^,^^26^ Target region 184–200 contains a U residue at a position 7 nt downstream of the CUC cleavage site for potential *trans*-Hoogsteen base pair formation with the GUGA tetraloop in the ribozyme core. Importantly, RNA motifs containing target sites 615–631, 628–644, and 688–704 were previously targeted by antisense oligonucleotides and small interfering RNAs (siRNAs).^25^^,^^26^ Tqhese studies indicated that targeting selected regions leads to effective viral inhibition. Moreover, the designed hammerhead ribozymes are able to target both segment 5 mRNA and cRNA, as they share the same sequence (with the exception of the extreme 5′ and 3′ ends, which are shortened in mRNA).

### Design of the hammerhead ribozymes

**Figure 2:**
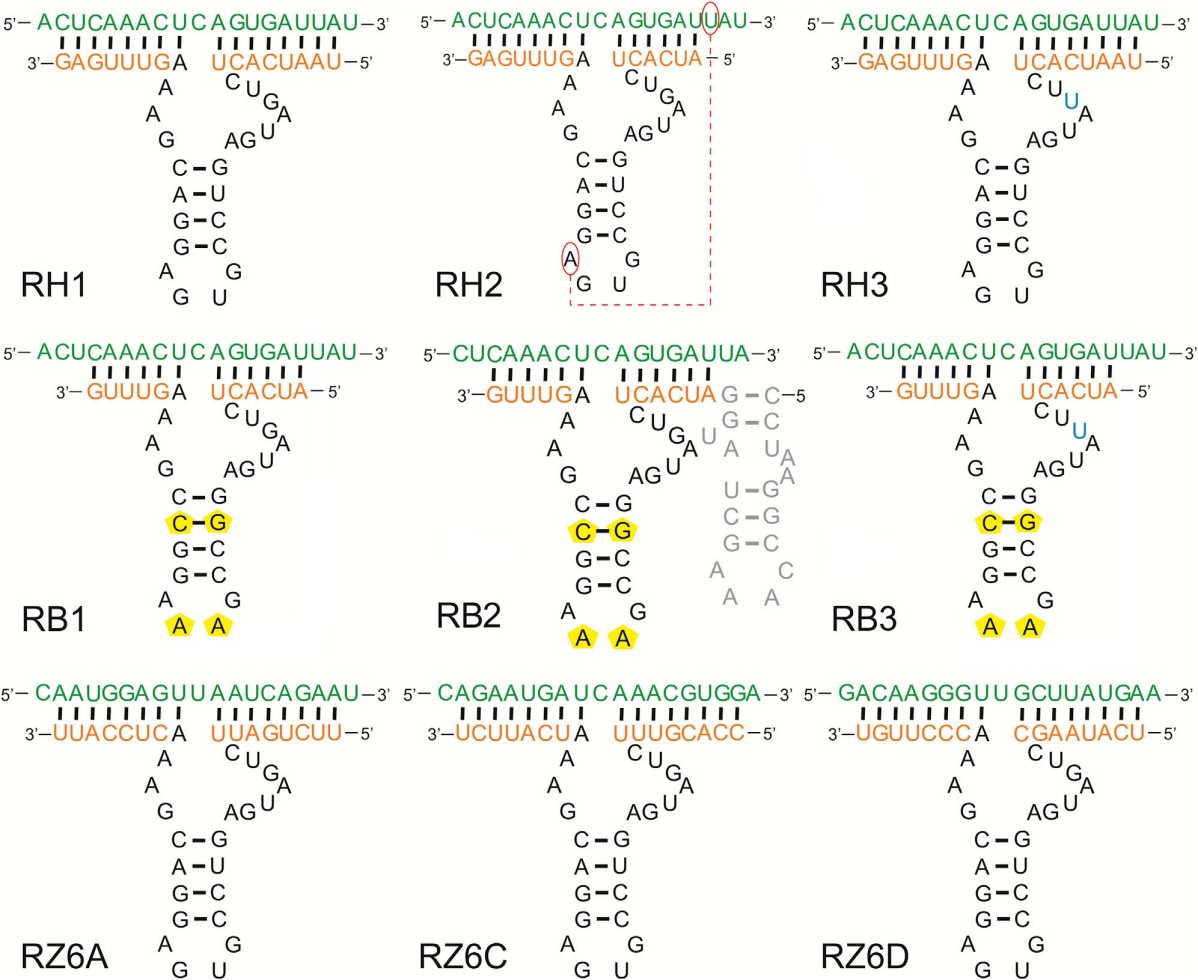
Model hammerhead ribozymes with the RNA target sequences Target RNA sequences are shown in green, and the side arms of the ribozymes are shown in orange. Nucleosides that may be involved in *trans*-Hoogsteen iterations are marked in red, and changes in hammerhead stem II and loop L2 are marked in yellow.

All ribozyme sequences are listed in Table 1. RH1 is a standard minimal hammerhead ribozyme. RH2 is a variant containing a 6-nt-long 5′ arm to allow potential tertiary interactions between target RNA containing the U residue at a position 7 nt downstream of the cleavage site and the A residue in the ribozyme GUGA tetraloop (Figure 2). Both of these ribozymes target the (+)RNA5 region starting at nucleotide 184 (Figure S1). RB1 is another version of the standard minimal hammerhead ribozyme, which differs from RH1 in the sequence of ribozyme stem loop II. Its core became the basis for the RB2 variant containing a TLR hairpin motif that stabilizes the active ribozyme conformation in accordance with previously published data.^42^ The target region starts at nucleotide 186 in the (+)RNA5 sequence. Three ribozymes based on the RH1 core, targeting regions 615–631 (RZ6A), 630–644 (RZ6C), and 688–704 (RZ6D), were also designed to assess the dependence of target site selection on the inhibitory potential. As an additional control, ribozymes RH3 (based on the RH1 core) and RB3 (based on the RB1 core) were used, which contain a single G5 to U mutation in the catalytic core of the ribozyme to impair its activity (Table 1).

**Table 1:** Sequences of the analyzed hammerhead ribozymes

Names	Sequences of the hammerhead ribozymes	Target ((+)RNA numbering)	Cleavage NUH triplet
RH1NL	5'-UAAUCACU**CUGAUGAGUCCGUGAGGACGAA**AGUUUGAG-3'	184–200	CUC
RH1	5'-UAAUCACU**CUGAUGAGUCCGUGAGGACGAA**AGUUUGA**G**^**L**^-3'		
RH2	5'-AUCACU**CUGAUGAGUCCGUGAGGACGAA**AGUUUGAG-3'	184–198	
RH3	5'-UAAUCACU**CU*U*AUGAGUCCGUGAGGACGAA**AGUUUGAG-3'	184–200	
RB1	5'-AUCACU**CUGAUGAGGCCGAAAGGCCGAA**AGUUUG-3'	186–198	
RB2	5'-CCUAAGGCCAAAGCUAUGGAUCACU**CAGAUGCGGCCGAAAGGCCGUA**AGUUUG-3'		
RB3	5'-AUCACU**CU*U*AUGAGGCCGAAAGGCCGAA**AGUUUG-3'		
RZ6A	5'-UUCUGAUU**CUGAUGAGUCCGUGAGGACGAA**ACUCCAU**U**^**L**^-3'	615–631	GUU
RZ6C	5'-CCACGUUU**CUGAUGAGUCCGUGAGGACGAA**AUCAUUC**U**^**L**^-3'	628–644	AUC
RZ6D	5'-UCAUAAGC**CUGAUGAGUCCGUGAGGACGAA**ACCCUUG**U**^**L**^-3'	688–704	GUU

The sequence of the hammerhead ribozyme catalytic core is marked as bold letters, and its mutations are indicated in italic. The TLR motif is underlined in the ribozyme sequence. The LNA residue is marked with **N**^**L**^.

Moreover, within RH1, RZ6A, and RZ6C, the catalytic cores were modified with guanosine analogs. RH1 was also modified with nucleoside derivatives in the tetraloop. These conformationally defined analogs of guanosine included arabinoguanosine, 2′-deoxyguanosine, 2′-deoxy-2′-fluoroguanosine, 2′-deoxy-2′-fluoroarabinoguanosine, 8-bromo-2′-deoxy-2′-fluoroarabinoguanosine, 8-bromoarabinoguanosine, and 8-bromoguanosine (Figure S2). The chosen derivatives of guanosine differ by only one or two functional groups at a time. The introduced changes influence the conformation of the sugar residue and/or N-glycosidic bond in the modified residue. Regarding the S_N_2 mechanism and conformation of the catalytic core of the hammerhead ribozyme, changes in the G12 and G8 conformations will be noticed by changes in the catalytic activity of the ribozyme.

### Cleavage kinetics of the modified hammerhead ribozymes

Twenty-five synthetic hammerhead variants with modified nucleoside residue were tested to evaluate their cleavage activity and calculate the observed cleavage rate constants (k_obs_ values). The collected results are presented in Table 4. In the case of all ribozymes, cleavage was observed in the expected site of the NUH triplet in the target 5ʹ end FAM-labeled oligomers (Figure S5). All modifications introduced into the catalytic core resulted in decreased ribozyme activity toward target RNA cleavage. In the case of ribozymes RH1-8-6 and RH1-8-8, there was no cleavage product observed after 1.5 h. The experiments for every hammerhead ribozyme variant were performed in quadruplicate. Kinetic curves for all tested variants of the modified ribozymes are given in Figure S6. Hammerhead ribozymes with modifications introduced in G8 showed greater changes in the reaction rate than ribozymes with G12 modification. Most of the hammerhead ribozymes modified in G8, such as RH1-8-3, RH1-8-4, RH1-8-5, and RH1-8-7, do not reach the kinetic cleavage plateau within 1.5 h. This suggests that the performance of these ribozymes is impaired compared with unmodified variants. After 1.5 h, ribozymes RH1-8-4 and RH1-8-5 showed less than 15% cleavage. Hammerhead ribozymes modified at position G12, such as RH1-12-3 and RH1-12-6, gave over 50% of substrate cleavage after 1.5 h of reaction, whereas ribozyme RH1-12-4 presented only 10% cleavage. The possible reason for these lower cleavage yields could be the conformation of the 8-bromoarabinoguanosine in G12. Even though RH1-12-3 contains arabinose, its puckering may be similar to that of 8-bromoarabinoguanosine, and RH1-12-6 contains 8-bromoguanosine, which favorably adopts the *syn* conformation, but the combined puckering of the sugar ring (arabinose) and the *syn* conformation of the base (8-bromoguanine) drastically lowers the yield of ribozyme RH1-12-4.

Next, the k_obs_ parameters were determined from the kinetic curves (Figure S6) and further used to select ribozymes for experiments investigating the inhibition of IAV replication in cell culture. Based on these data, only ribozymes with chemical modifications in G12 were used in the *in cellulo* experiments.

### Antiviral effect of the ribozyme variants targeting (+)RNA5 region 184–200

**Figure 3:**
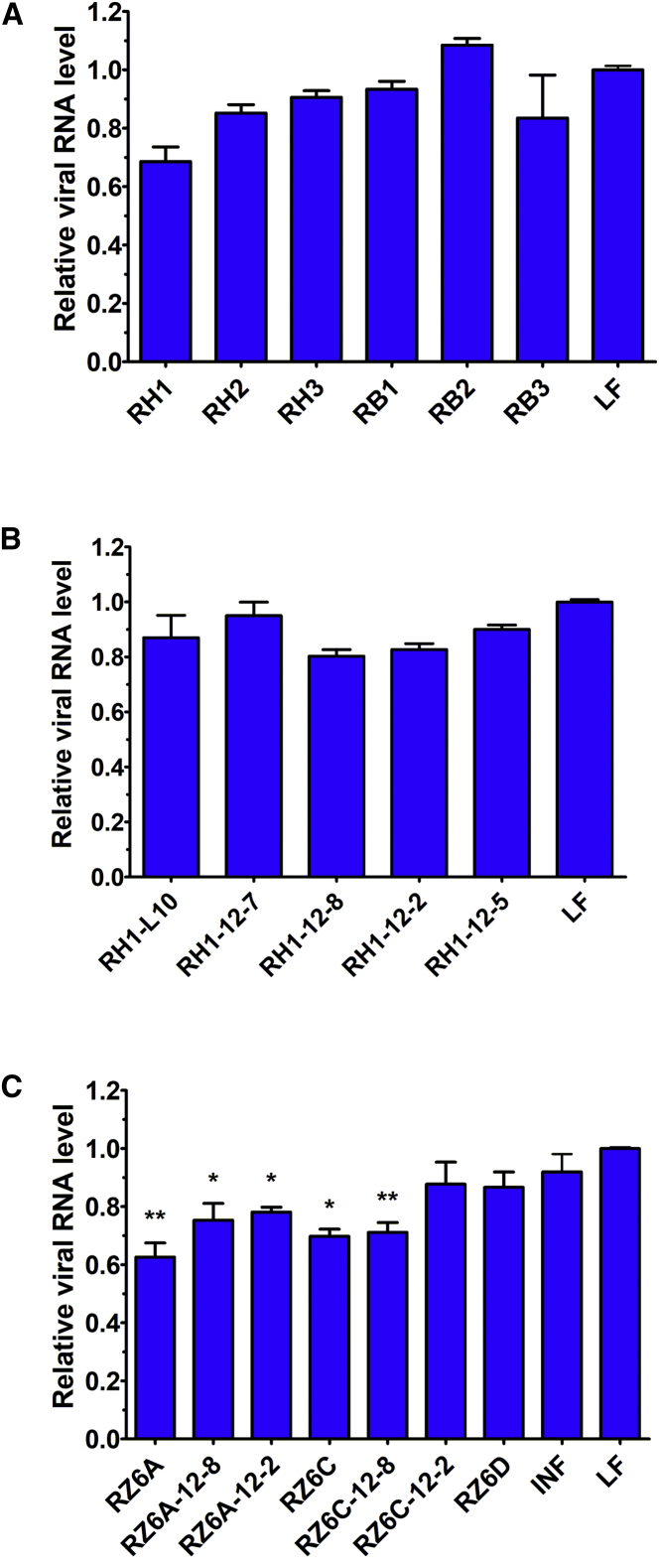
Antiviral activity of the ribozymes in MDCK cell culture based on real-time PCR quantitative analysis of viral RNA (A) Sequential and structural variants of the ribozymes targeting (+)RNA5 region 184–200. (B) Chemically modified variants of the ribozyme targeting (+)RNA5 region 184–200 are shown. (C) Ribozymes targeting (+)RNA5 region 615–704 are shown. The viral RNA level in the ribozyme-treated samples was compared with the Lipofectamine-treated control (LF). INF denotes the untreated infected cells. Error bars represent the standard error of the mean (SEM). An unpaired two-tailed t test was performed for statistical comparisons (∗p < 0.05 and ∗∗p < 0.01).

Selected ribozyme variants were tested against IAV in Madin-Darby canine kidney (MDCK) cell cultures infected with the A/California/04/2009 (H1N1) strain. According to real-time PCR analysis, the RH1 ribozyme caused 31.4% inhibition of viral replication in comparison to the Lipofectamine control (Figure 3A). This result was proven to be statistically insignificant (p > 0.05). The antiviral activity was also insignificant for the sequential and structural variants of the ribozyme, such as RH2 and RB1. No inhibitory effect was observed in the IAV titer from cells treated with RB2. In addition, all chemically modified RH1 ribozyme variants, RH1-L10, RH1-12-7, RH1-12-8, RH1-12-2, and RH1-12-5, caused insignificant changes in viral replication (Figure 3B). These data are similar to those obtained for the mutant ribozymes RH4 and RB3. The number of viral RNA copies in each sample was similar to that found in the Lipofectamine-treated control.

### Viral inhibition by ribozymes targeting (+)RNA5 region 615–704

The highest statistically significant inhibitory effect on IAV replication was obtained by the application of the ribozymes RZ6A and RZ6C, which reduced replication by 37.4% and 30.2%, respectively (Figure 3C). The chemically modified variants of these ribozymes also reduced the viral load; however, none of them exceeded the effect obtained by the primary unmodified ribozyme. Ribozymes RZ6A-12-8, RZ6A-12-2, and RZ6C-12-8 decreased the number of viral RNA copies by 24.7%, 21.9%, and 28.9%, respectively. The modification in RZ6C-12-2 resulted in almost complete loss of ribozyme inhibitory potential, and viral reduction reached 12.3%. In addition, RZ6D showed negligible antiviral potential, causing 13.1% IAV inhibition.

### Design of the plasmid constructs coding shRNA and hammerhead ribozymes

The ribozymes showing the most prominent inhibitory effects against influenza virus, such as RZ6A and RZ6C, were selected for the preparation of chimeric shRNA-ribozyme constructs (Table 2).^23^ These constructs combined the catalytic properties of ribozymes and the actions of siRNA through the RNA-induced silencing complex (RISC) cascade, finalized by enzymatic RNA cleavage by Ago2. As a precursor concomitant with both ribozymes joined by a cleavable linker, shRNA corresponding to the highly active siRNA 613 tested in our previous studies was used.^25^ As a result, the chimeric shRNA-ribozyme sh613RZ6A and sh613RZ6C were created. To assess the role of each molecule in the viral inhibition process, constructs were prepared carrying only ribozyme RZ6A or shRNA sh613. As an additional control, mutant ribozymes containing a single G5 to U point mutation in the catalytic core were also used in single-molecule construct mutRZ6A and the chimeric constructs sh613mutRZ6A and sh613mutRZ6C.

**Table 2:** Sequences of the chimeric constructs

Names	Sequence of chimeric constructs
sh613RZ6A	5'-GCAATGGAGTTAATCAGAATTCAAGAGATTCTGATTAACTCCATTGCTTCAACTTACGTTTCTGATGAGTCCGTGAGGACGAAATCATTCT-3'
sh613RZ6C	5'-GCAATGGAGTTAATCAGAATTCAAGAGATTCTGATTAACTCCATTGCTTCAACTTCTGATTCTGATGAGTCCGTGAGGACGAAACTCCATT-3'
sh613mutRZ6A	5'-GCAATGGAGTTAATCAGAATTCAAGAGATTCTGATTAACTCCATTGCTTCAACTTACGTTTCTTATGAGTCCGTGAGGACGAAATCATTCT-3'
sh613mutRZ6C	5'-GCAATGGAGTTAATCAGAATTCAAGAGATTCTGATTAACTCCATTGCTTCAACTTCTGATTCTTATGAGTCCGTGAGGACGAAACTCCATT-3'

### Antiviral potential of the constructs coding shRNA and hammerhead ribozymes delivered to cells as plasmids

**Figure 4:**
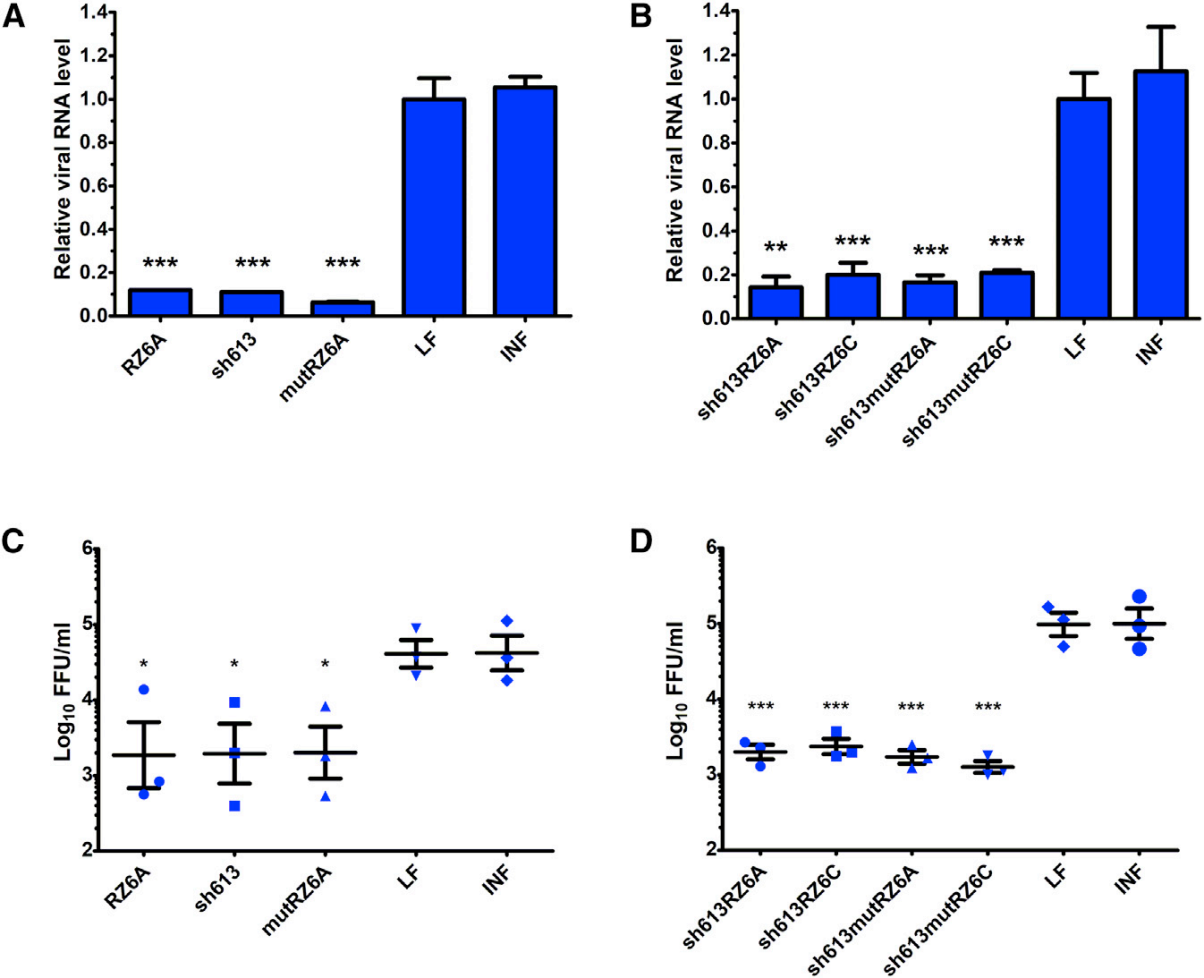
Antiviral potential of plasmid constructs (A and B) Inhibitory potential of the tested plasmid constructs based on quantitative real-time PCR analysis of viral RNA in samples treated with (A) single-molecule constructs and (B) shRNA-ribozyme constructs. (C and D) Analysis of infectious viral particles by FFA in samples treated with (C) single-molecule constructs and (D) shRNA-ribozyme constructs. Error bars represent the SEM. An unpaired two-tailed t test was performed for statistical comparisons (∗p < 0.05, ∗∗p < 0.01, and ∗∗∗p < 0.001).

Plasmid constructs were tested against IAV in MDCK cell cultures infected with the A/California/04/2009 (H1N1) strain. All of the tested constructs caused significant inhibition of IAV replication in comparison to the Lipofectamine-treated control. Real-time PCR analysis revealed a significant reduction in the viral RNA copy number in the samples treated with the single-molecule constructs. For sh613, inhibition reached 88.8%, and for RZ6A, it reached 88.0% (Figure 4A). Interestingly, comparable, high antiviral potential was also observed for mutRZ6A. The chimeric constructs carrying both shRNA and ribozyme significantly inhibited viral replication; however, none of them were able to exceed the effect obtained by constructs coding only shRNA or hammerhead ribozyme. Inhibitory effects of 85.9% and 80.0% were observed in the samples treated with sh613RZ6A and sh613RZ6C, respectively (Figure 4B). A comparable decrease was also detected for the constructs carrying the mutated, catalytically inactive ribozymes sh613mutRZ6A or sh613mutRZ6C.

The antiviral effects of the plasmid constructs were also investigated with focus formation assay (FFA). This method is based on the analysis of infectious virus particle production. The single-molecule constructs sh613 and RZ6A reduced the viral load by 1.0 and 1.3 log_10_ units, respectively. Application of chimeric sh613RZ6A and sh613RZ6C led to high inhibition of virus replication (1.6 and 1.7 log_10_ units, respectively). This method also confirmed the high and significant antiviral effects of mutRZ6A, sh613mutRZ6A, and sh613mutRZ6C.

## Discussion

### Nucleoside substitution in the catalytic core hinders ribozyme activity

Hammerhead ribozymes with introduced modified nucleosides differ only by one or two groups relative to the unmodified variants (Figure S2). The modifications of guanosine used were designed to initiate a pucker in the case of ribose analogs and/or the *syn-anti* conformation of the glycosidic bond. In the case of ribozymes modified in the G8 position, such as RH1-8-2, RH1-8-3, RH1-8-4, RH1-8-5, RH1-8-6, RH1-8-7, RH1-8-8, and RH1-8-9 (Table 3), the target RNA cleavage yields were significantly lower than those with the unmodified ribozyme. The 2ʹ-OH moiety of G8 in the catalytic core acts as the general acid in the proposed mechanism. It is also known that the G8 base is involved in Watson-Crick pairing with C3.^17^^,^^20^ The lowered yields indicate that the sugar residue of G8 cannot be changed without loss of ribozyme activity. Interaction of the 2′-hydroxyl with the cleavage site is impossible for all 2′-deoxy analogs introduced into G8. The ribozymes RH1-8-5 and RH1-8-7 containing 2′-deoxyguanosine and 2′-fluoro-2′-deoxyarabinoguanosine in G8, respectively, showed residual activity. This result may suggest that another mechanistic pathway is involved. Only in the case of the 8-bromoguanosine-substituted hammerhead ribozymes did variants of RH-8-6 and RH-8-8 show no activity in the kinetic assay experiment. This must be due to the *syn* conformation of the nucleobase (8-bromoguanine) disrupting the necessary G8-C3 base pairing in the catalytic core.^20^ This indicates that the conformation of nucleobase G8 is as important for hammerhead cleavage activity as the presence and configuration of the 2′-hydroxyl group. Modifications introduced into G12 of this ribozyme seem to have less influence on the cleavage kinetics. Changes in the sugar residue of G12 mainly affected ribozyme activity in the case of substitution with 2′-fluoro-2′-deoxyguanosine in RH1-12-7. This could indicate that the substituted sugar ring changes the availability of N1 of the nucleobase to act as a general base in this reaction.^43^, ^44^, ^45^ A similar lowering in the yields was expected in the case of ribozymes with 8-bromoguanine because the *syn* conformation should be adopted. Whereas 8-bromoguanosine derivatives introduced in G12 slightly influenced the ribozyme activity, RH1-12-4 showed a reaction rate similar to that of RH1-12-7, with a k_obs_ equal to 0.002 min^−1^ (Table 4).

**Table 3:** Modified nucleotide residues included in the hammerhead ribozymes

Name	Ribozyme variant	Included modification	Modification site
RH1-12-2	RH1	8-bromoguanosine	G12
RH1-12-3	RH1	arabinoguanosine	G12
RH1-12-4	RH1	8-bromoarabinoguanosine	G12
RH1-12-5	RH1	2′-deoxyguanosine	G12
RH1-12-6	RH1	8-bromo-2′-deoxyguanosine	G12
RH1-12-7	RH1	2′-deoxy-2′-fluoroarabinoguanosine	G12
RH1-12-8	RH1	8-bromo-2′-deoxy-2′-fluoroarabinoguanosine	G12
RH1-12-9	RH1	2-aminoadenosine	G12
RH1-8-2	RH1	8-bromoguanosine	G8
RH1-8-3	RH1	arabinoguanosine	G8
RH1-8-4	RH1	8-bromoarabinoguanosine	G8
RH1-8-5	RH1	2′-deoxyguanosine	G8
RH1-8-6	RH1	8-bromo-2′-deoxyguanosine	G8
RH1-8-7	RH1	2′-deoxy-2′-fluoroarabinoguanosine	G8
RH1-8-8	RH1	8-bromo-2′-deoxy-2′-fluoroarabinoguanosine	G8
RH1-8-9	RH1	2-aminoadenosine	G8
RH1-L10	RH1	UNA-uridine	U2.2
RH1-L11	RH1	UNA-isoguanosine	G2.3
RZ6A-12-2	RZ6A	8-bromoguanosine	G12
RZ6A-12-8	RZ6A	8-bromo-2′-deoxy-2′-fluoroarabinoguanosine	G12
RZ6C-12-2	RZ6C	8-bromoguanosine	G12
RZ6C-12-8	RZ6C	8-bromo-2′-deoxy-2′-fluoroarabinoguanosine	G12

**Table 4:** Calculated kinetic parameters of the modified ribozymes

Observed reaction rate k_obs_ (min^−1^)					
RH1	0.112	RH1-8-2	0.022	RH1-L10	0.108
RH1-12-2	0.042	RH1-8-3	0.013	RH1-L11	0.008
RH1-12-3	0.040	RH1-8-4	0.004	RZ6A	0.015
RH1-12-4	0.002	RH1-8-5	0.003	RZ6A-12-2	0.003
RH1-12-5	0.057	RH1-8-6	n/n	RZ6A-12-8	0.008
RH1-12-6	0.026	RH1-8-7	0.002	RZ6C	0.128
RH1-12-7	0.002	RH1-8-8	n/n	RZ6C-12-2	0.020
RH1-12-8	0.077	RH1-8-9	0.029	RZ6C-12-8	0.093
RH1-12-9	0.032				

Moreover, modification in hairpin loop L2 with unlocked nucleic acid (UNA) analogs of uridine and isoguanosine^46^, ^47^, ^48^ resulted in an almost unchanged reaction rate for RH1-L10 but almost completely stopped the reaction for RH1-L11. This result indicates that the isoguanosine residue in the loop significantly changed the interaction in the ribozyme, which led to nearly no cleavage activity by ribozyme RH1-L11 (Table 4).

Despite their low k_obs_ values, nine modified ribozymes, RH1-12-2, RH1-12-5, RH1-12-7, RH1-12-8, RH1-L10, RZ6A-12-2, RZ6A-12-8, RZ6C-12-2, and RZ6C-12-8, were transfected into the MDCK cell line, and their antiviral activities against the IAV were verified. The *in cellulo* experimental results were consistent with the outcomes of the kinetic assays for the modified ribozymes. Other conclusions regarding ribozyme performance in cells are discussed below.

### Limited utility of the approaches that stabilize hammerhead ribozymes

The presented results show that the application of catalytic nucleic acids in biological systems is challenging. The introduction of a hairpin motif or the selection of target-ribozyme pairs with sequence features enabling potential *trans*-Hoogsteen interactions, both of which promote stabilization, did not result in the desired increase in catalytic activity as reported previously.^3^^,^^4^ However, in our studies, these variants were proposed for the hammerhead ribozyme RH1 with initially low inhibitory potential, which may have diminished the effect. The conditions in which the improvement of features and the high activity of the minimal ribozyme variants are observed require clarification and more detailed analyses. The design of the RH2 ribozyme was based solely on the selection of target and ribozyme sequences that could potentially enable *trans*-Hoogsteen interactions. However, the complete sequences neighboring the interaction site differ from known crystallized ribozyme examples showing the abovementioned tertiary contacts. Further analysis of the sequence and structural context of this target-ribozyme pair as well as preparation of other structural models could be helpful to interpret the observed effects. The results obtained for potentially inactive mutant ribozymes that showed inhibition levels similar to the primary ribozyme support previous reports suggesting potential antisense effects of catalytically inactive ribozymes.^49^^,^^50^ However, it should also be taken into consideration that the catalytic cleavage performed by ribozymes is expected to be a multiple-turnover mechanism that is more efficient than steric hindrance caused by antisense oligonucleotides, which present single-turnover kinetics. The exact mechanism of action of mutant ribozymes remains to be elucidated.

### The antiviral activity of the ribozyme depends on the target site

There are many factors influencing the biological activity of ribozymes, such as various ions and pH values as well as the presence of biomolecules.^51^ The selection of a target sequence and its structural context, particularly within the nucleotide triplet at the RNA cleavage site, may be important.^52^ To date, it has been shown that the availability of the target RNA region and the specific sequence of the NUH triplet determine the activity of the ribozyme.^9^^,^^16^^,^^42^^,^^53^ Not all nucleotide triplets provide highly efficient target RNA cleavage. In published reports, the most effective hydrolysis was observed for GUC and AUC triplets.^16^^,^^42^ Only one of the ribozymes tested in this study was designed within the optimal nucleotide NUH triplet. This was the RZ6C ribozyme, which showed great potential to inhibit IAV replication in cell-culture studies. In addition, the target triplet for RZ6C was located in the single-stranded loop, which was accessible for the previously tested antisense oligonucleotides.^26^ The most active ribozyme, RZ6A, cleaved influenza RNA after the GUU triplet, as did the ribozyme RZ6D, which gave a low yield. These results show that the efficiency of ribozyme-initiated catalytic cleavage is not exclusively dependent on the type of target NUH triplet. It was previously reported that the sequence context of the cleavage triplet is also important; however, no clear rules were determined.^54^ On the other hand, when considering the structural context of the cleavage site, all triplets except AUC (RZ6C) were located in regions either partially or fully involved in the formation of double-stranded structures. However, they were also preceded by a string of unpaired nucleotides to enable potential hybridization of at least one of the ribozyme arms. In this case, it is not possible to clearly indicate how the target region structure influences the activity of the tested ribozyme. Information on tertiary structure, RNA-protein interactions, and RNA dynamics could explain the observation. However, the target region of the two most active hammerhead ribozymes, RZ6A and RZ6C, was the domain at which the effective ASO and siRNA were directed in previous studies.^25^^,^^26^ The second region (682–700 nt) with a similar inhibition profile mediated previously by ASO and siRNA did not prove to be a suitable target region for RZ6D ribozyme effectiveness. These results show that, while the most effective oligonucleotide tools operate within the same domains, not every region of the viral RNA structure may be a universal target for a variety of RNA-targeting strategies. The final effect is probably dependent on a number of factors, all of which simultaneously affect *in cellulo* target cleavage. Inhibitory approaches based on sense-antisense interactions proceed through distinct intracellular pathways, and their performance criteria may differ slightly.

### Increased antiviral potential of constructs coding shRNA and hammerhead ribozymes delivered in plasmids

The obtained results indicate that ribozyme plasmid constructs have a higher potential to inhibit IAV replication than ribozymes delivered directly into the cells via the Lipofectamine reagent. According to real-time PCR analysis of viral RNA copies, a greater than 50% increase in antiviral activity occurred when RZ6A was cloned into the plasmid. This may be due to the continuous expression of the ribozyme from the plasmid vector within the cell during viral infection. Direct transfection of ribozyme RNA is a single event affected by the cell penetration efficiency and RNA degradation during the process. The foreign RNA may also be degraded within the cell after successful entry. These events, which do not apply to plasmids, lead to a decrease in the ribozyme RNA concentration and lower effective target cleavage.

Another aspect is the contribution of the plasmid construct components to the overall inhibitory effect. Quantitation of viral RNA showed that inhibition of viral replication reached similar levels after treatment with constructs coding both shRNAs and ribozyme and constructs coding only shRNA or ribozyme. However, analysis of the infectious viral particles indicated that the influence of chimeric constructs is more effective than constructs coding only shRNA or ribozyme. The inhibitory effect of the sh613RZ6A construct increased by 0.6 log_10_ units compared with the sh613 plasmid construct and 0.3 log_10_ units in the case of the RZ6A plasmid (Figures 4C and 4D). Plasmid sh613RZ6C causes viral inhibition that is 0.7 log_10_ units higher than the sh613 construct.

### Conclusions

All of the chemically modified hammerhead ribozymes in this study showed lower catalytic activity than the unmodified ribozymes. Our explanations for these results are based on studies of different models and their crystal structures. More experiments involving pH change, ribozyme crystallization and molecular dynamics or quantum mechanics calculations would be useful for further understanding how each modification used here influenced the active and stationary state of the ribozyme catalytic core. The obtained results also indicate that variants with the potential to increase the structural stabilization of the ribozyme (carrying the TLR motif or potential *trans*-Hoogsteen interactions) and its catalytic activity should be further investigated to reveal critical factors determining successful property improvement. Even the analysis of the unmodified minimal ribozyme variants showed that multiple factors may affect ribozyme activity, including target site and sequence selection; however, our knowledge in this area is still limited. In this study, the highest inhibitory potential was achieved by the application of plasmid constructs. The maximum reduction in the viral load (1.7 log_10_ units) was obtained by application of the chimeric construct sh613RZ6C.

## Materials and methods

### Materials

Most reagents used during the synthesis of the protected derivatives of guanosine were purchased from Sigma-Aldrich. Dimethoxytrityl chloride, 2-cyanoethyl N,N,N′,N′-tetraisopropylphosphorodiamidite, and 1,1,3,3-tetraisopropyldisiloxane-1,3-dichloride were purchased from ChemGenes. Solvents used were of the highest purity available and dried with molecular sieves if necessary. The stepwise chemical synthesis of 3′-O-phosphoramidite of protected 2′-fluoro-2′-deoxyarabinoguanosine and 8-bromo-2′-fluoro-2′-deoxyarabinoguanosine is described in detail in supplemental information S3.

### Synthesis and deprotection of the oligonucleotides

Oligonucleotides were synthesized on a BioAutomation MerMade12 DNA/RNA synthesizer using β-cyanoethyl phosphoramidite chemistry^37^ and commercially available phosphoramidites (ChemGenes and GenePharma). Deprotection and purification of the oligonucleotides were accomplished according to previously published procedures.^55^ However, deprotection of the oligonucleotides with an 8-bromoguanosine residue with aqueous ammonia/ethanol (3:1 v/v) was performed for 48 h at room temperature.

### *In vitro* cleavage assay (kinetic assay)

Cleavage reactions were performed in a 30-μL volume of 500 nM ribozyme and 5 mM FAM 5ʹ end-labeled RNA target substrate in the presence of 50 mM Tris-HCl (pH 7.5) and 5 mM MgCl_2_ at 25°C. The sequences of the target substrates were as follows: for RH1, 5'-FAM-ACUCAAACUCAGUGAUUAU-3' ; for RZ6A, 5'-FAM-CAAUGGAGUUAAUCAGAAU-3'; and for RZ6C, 5'-FAM-CAGAAUGAUCAAACGUGGA-3'. The same oligonucleotides were used for the modified ribozymes. Stock solutions of ribozyme and substrate were prepared in 50 mM Tris-HCl (pH 7.5), heated separately at 90°C for 1 min, and cooled to 25°C. Next, MgCl_2_ was added to both solutions to obtain a 5 mM concentration. The reaction was initiated by the addition of ribozyme to the substrate. Aliquots of 3 μL were removed at predetermined time intervals (0, 0.5, 1, 2.5, 5, 15, 30, 45, 60, and 90 min) and quenched with 17 μL of stop mix (5 mM sodium citrate, 7 M urea, and 200 mM EDTA [pH 5]). The cleavage reaction was analyzed by electrophoresis on a 12% polyacrylamide 8 M urea gel and visualized with PhosphoImager (Fuji FLA-5100) (Figure S5). The bands were quantified, and the k_obs_ parameters were obtained by fitting to *f = 1 − e*^*−kt*^, where *f* is the fraction cleaved at time *t*.

### Cell culture and virus titration

MDCK cells (Sigma) were cultured as previously described.^25^ Experiments were carried out on the A/California/04/2009 (H1N1) influenza strain (a gift from Prof. Luis Martinez-Sobrido, Texas Biomedical Research Institute, USA) propagated in MDCK cells. A standard plaque assay determined the virus titer.

### Plasmid preparation

The DNA sequences of the ribozyme, shRNA, and shRNA-ribozyme chimeric constructs were chemically synthesized. Each insert was flanked with restriction site sequences for HindIII and BamHI restriction endonucleases. Digestion was performed using FastDigest HindIII and FastDigest BamHI restriction enzymes (Thermo Fisher Scientific) according to the manufacturer’s protocol, followed by phenol-chloroform extraction and ethanol precipitation. The inserts were cloned into a pcDNA3.1(+) vector using T4 DNA ligase (EURX) according to the manufacturer’s recommendation.

### Ribozyme and plasmid transfection into the cells

Transfection was carried out using Lipofectamine 2000 (Invitrogen), which was diluted in Opti-MEM (Invitrogen) according to the manufacturer’s instructions and incubated for 10 min at room temperature (RT). Next, ribozyme RNA or plasmid was diluted with the appropriate volume of Opti-MEM, mixed gently with Lipofectamine-Opti-MEM solution, and incubated for 30 min at RT. The final concentration of ribozyme during transfection was 200 nM, or 500 ng of plasmid construct was added per well. Just before transfection, the MDCK cells were trypsinized and resuspended in fresh culture medium. Transfection solution (100 μL) was added to the MDCK cell suspension containing 1.4 × 10^5^ cells per well at a final volume of 600 μL, which was then seeded in 24-well plates.

### Viral infection of the cell culture

Eighteen hours after transfection, the cell cultures were washed with PBS and infected with influenza virus A/California/04/2009 (H1N1) at a multiplicity of infection (MOI) of 0.01. The cells were incubated with a solution containing virus diluted with infection medium (0.3% BSA [Sigma], 100 U/mL penicillin, and 100 μg/mL streptomycin [penicillin-streptomycin; Sigma]), 0.1 mM CaCl_2_·2H_2_O, and 0.1 mM MgCl_2_·6H_2_O in PBS for 1 h at RT on a gently rocking platform. The supernatant was then removed, and the cells were maintained in postinfection medium (0.3% BSA, 100 U/mL penicillin, 100 μg/mL streptomycin, 2 mM glutamine, and 1 μg/mL N-tosyl-L-phenylalanine chloromethyl ketone (TPCK)-treated trypsin [Sigma] in DMEM) at 33°C for 24 h.

### Quantitative real-time PCR analysis of viral RNA

Total RNA from the cell monolayer was extracted using TRIzol reagent.^56^ DNase treatment, reverse transcription, and real-time PCR were carried out as previously described.^25^ The viral RNA copy number for each sample was calculated as a percentage of the viral copy number detected in the negative control (Lipofectamine-treated cells), which was established as 100% of the viral RNA copies.

### Virus titration by focus formation assay

Supernatants collected from infected cell cultures were used to prepare 10-fold serial dilutions of the virus in infection medium. Next, 50 μL of each dilution was inoculated onto confluent monolayers of MDCK cells cultured in 96-well culture plates. After 1 h of incubation on a gently rocking platform at RT, the supernatants were replaced with 100 μL of postinfection medium containing Avicel (0.2% BSA, 67 U/mL penicillin, 67 μg/mL streptomycin, 1.33 mM glutamine, 2.5 μg/mL N-acetylated trypsin [Sigma], and 1% Avicel in DMEM) and maintained for 24 h at 33°C under 5% CO_2_. The supernatants were discarded, and the cells were fixed and permeabilized with 4% formaldehyde and 0.5% Triton X-100 solution (BioShop) in PBS for 20 min at RT. Blocking was performed using 3% BSA in PBS for 1 h at RT. Next, the solution was replaced with the mouse anti-influenza primary antibody targeting nucleoprotein (NP) (MAB8257 Merck) diluted in PBS (1 μg/mL) and incubated for 1 h at RT. Detection was carried out with an fluorescein isothiocyanate (FITC)-conjugated secondary rabbit anti-mouse immunoglobulin G (IgG) antibody (AP160F Merck) diluted in PBS (1:150 v/v) for 30 min at RT. Visualization under a fluorescence microscope was followed by calculating the focus-forming units (FFUs)/mL.

### Statistical analysis

Statistical analysis of the data from three independent experiments (each containing three technical repeats) was performed using GraphPad Prism 5 software. A two-tailed t test with unequal variance was conducted, and three intervals of statistical confidence were considered: 0.05, 0.01, and 0.001.

## Supplemental information

Document S1. Figures S1–S6 and Scheme S1

Document S2. Article plus supplemental information
