# Supplementary material for: Structural variants and modifications of hammerhead ribozymes targeting influenza A virus conserved structural motifs
Source: Mol Ther Nucleic Acids. 2022 May 31;29:64–74. doi: 10.1016/j.omtn.2022.05.035 (PMC9217987; doi:10.1016/j.omtn.2022.05.035)
Supplement: Document S1. Figures S1–S6 and Scheme S1 [file mmc1.pdf]

OMTN, Volume *29*

Supplemental information

Structural variants and modifications of hammerhead ribozymes targeting influenza A virus conserved structural motifs

Tomasz Czapik, Julita Piasecka, Ryszard Kierzek, and Elzbieta Kierzek

S1. (+)RNA5 A/California/04/2009 (H1N1) secondary structure.

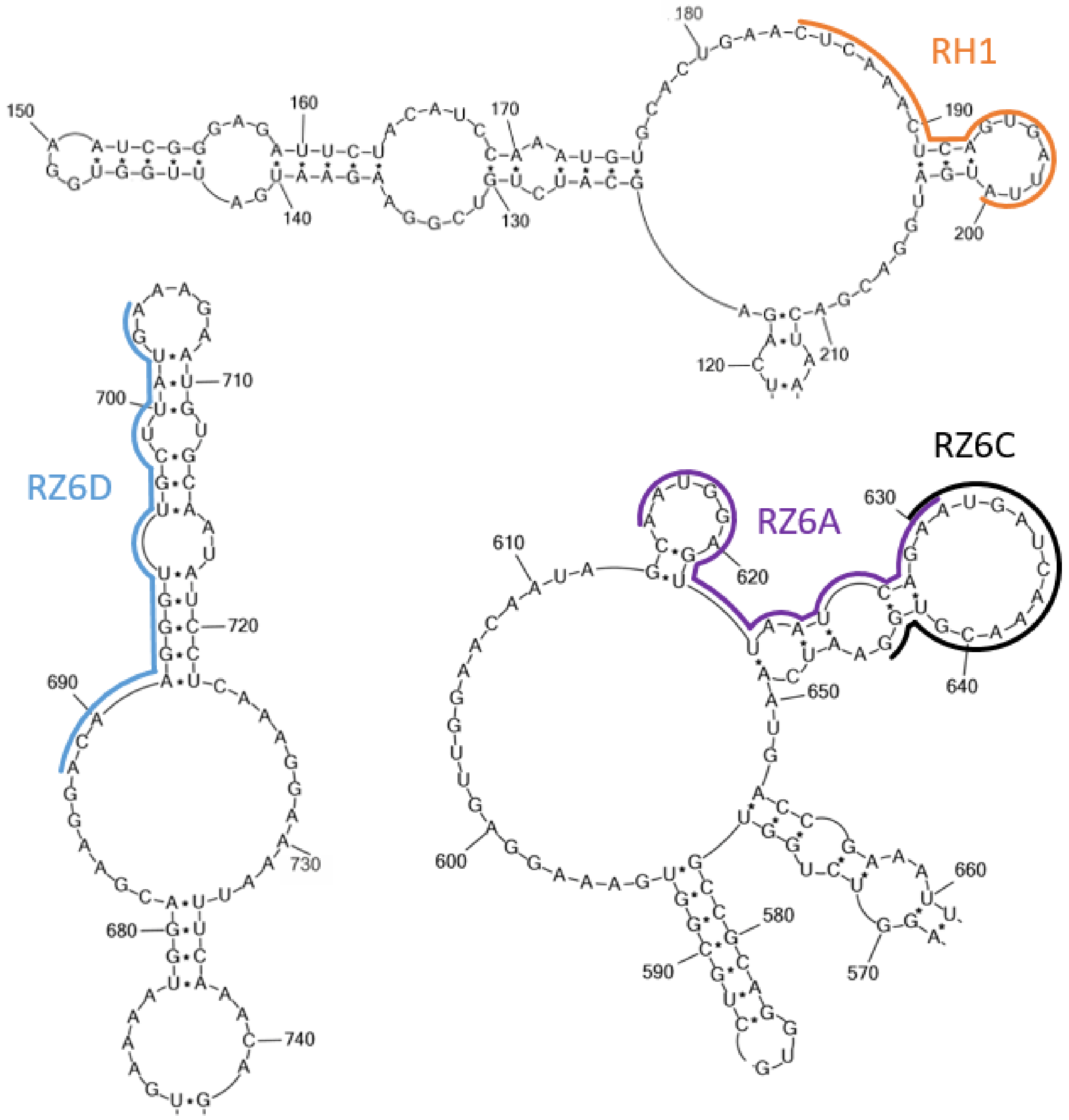

Figure S1. Target regions of hammerhead ribozymes in the (+)RNA5 A/California/04/2009 (H1N1) secondary structure. Secondary structure of (+)RNA5 A/California/04/2009 (H1N1) was previously published[1]. Target regions for analyzed hammerhead ribozymes are marked with colored lines.

S2. Modified nucleosides .

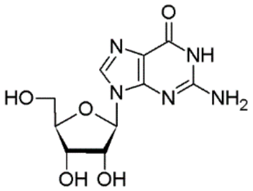

guanosine

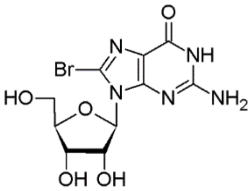

8-bromoguanosine

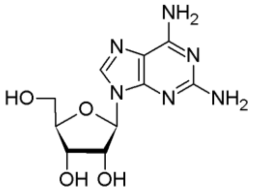

2-aminoadenosine

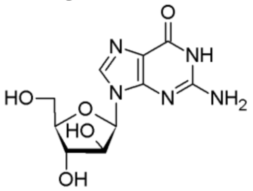

arabinoguanosine

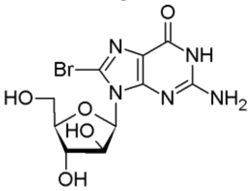

8-bromoarabinoguanosine

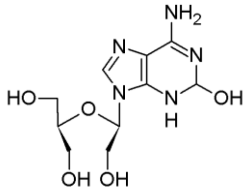

UNA-isoguanosine

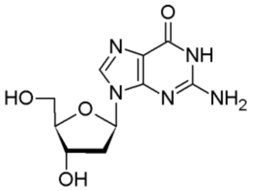

2′-deoxyguanosine

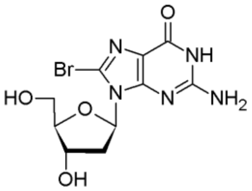

8-bromo-2′-deoxyguanosine

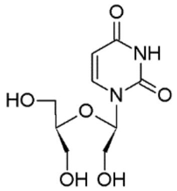

UNA-uridine

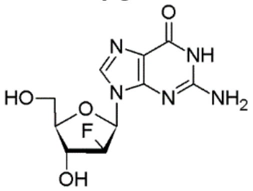

2′-deoxy-2′-fluoroarabinoguanosine

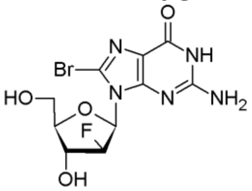

8-bromo-2′-deoxy-2′-fluoroarabinoguanosine

Figure S2. Structural formulas of guanosine derivatives.

S3. Nucleoside and Phosphoroamidite synthesis.

General. Guanosine, adenosine and 2,6-diaminopurine riboside used for synthesis and most other reagents were purchased from Sigma-Aldrich and Merck. Dimethoxytrityl chloride and 2-cyanoethyl N,N,N′,N′-tetraisopropylphosphorodiamidite were purchased from ChemGenes. Solvents were used at the highest available purity and dried with molecular sieves, if necessary.

NMR spectra were acquired using a Bruker Avance II (400 MHz) or Avance III (500 MHz) or Avance III (700 MHz) spectrophotometer. For ^{1}H, ^{13}C, ^{19}F and ^{31}P NMR spectra TMS, H_3PO_4 and trifluoroacetic acid, respectively, were used as references.

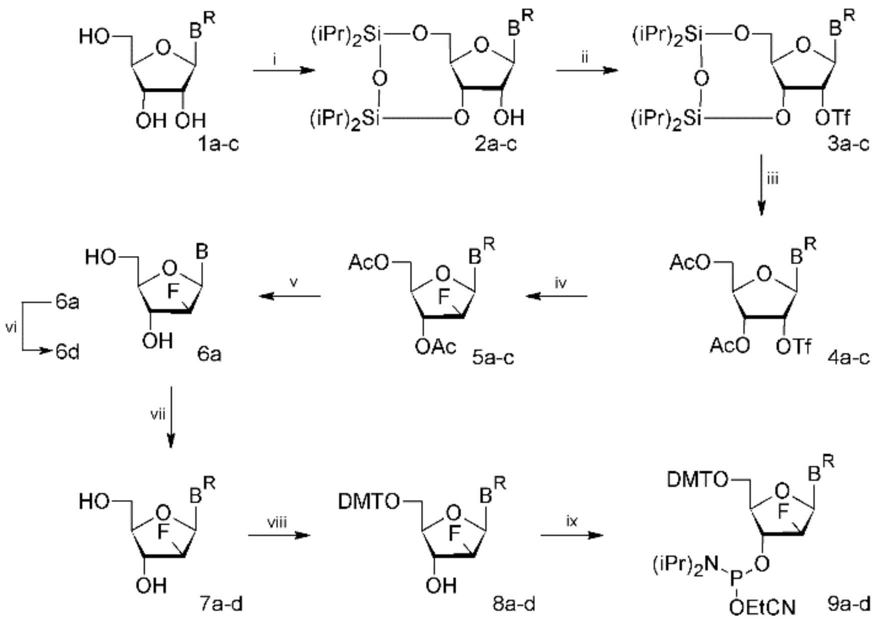

Scheme S1. Synthesis of 2′-deoxy-2′-fluoropurinearabinosides marked as following: a – guanine, b – adenine, c – 2,6-diaminopurine, d – 8-bromoguanine, respectively. Where: i means 1,3-dichloro-1,1,3,3-tetraisopropyldisiloxane/pyridine; ii means triflate anhydride, DMAP/dichloromethane, pyridine; iii means triethylammonium fluoride (TEAHF), acetic anhydride/pyridine; iv means TEAHF/THF, pyridine; v means ammonia/methanol; vi means bromine/water; vii means dimethylformamide dimethyl acetal/methanol; viii means 4,4′-

dimethoxytrityl chloride/pyridine and iv means 2-cyanoethyl N,N,N′,N′-tetraisopropylphosphoramidite, terazole/acetonitrile.

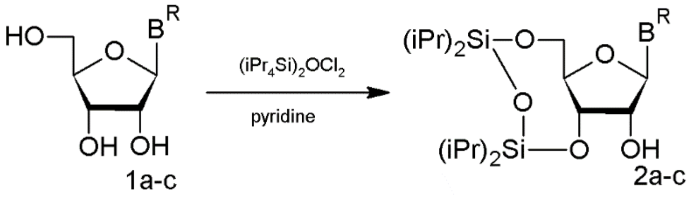

N-protected-3′,5′-O-(tetraisopropyldisiloxane-1,3-diyl)-purine riboside (step i). N-protected purine riboside (**1**) (1 equiv) suspension was evaporated twice with anhydrous pyridine. The dried residue was suspended in pyridine (5 ml per 1 mmol) and 1,1,3,3-tetraisopropyldisiloxane-1,3-dichloride (1.1 equiv) was added. After 3 h at room temperature (rt), the reaction was completed, worked up with aqueous solution of sodium bicarbonate, and extracted 3 times with dichloromethane. The combined organic layers were dried with anhydrous sodium sulfate, filtered and evaporated to obtain product (**2**) as white foam. Yield ca. 100 %.

N^2-(dimethylaminomethylene)-3′,5′-O-(tetraisopropyldisiloxane-1,3-diyl)-guanosine (**2a**). ^{1}H NMR (400 MHz, dmso-d_6), δ, ppm: 11.34 (s, 1H, NH), 8.54 (s, 1H, CH dmm), 7.85 (s, 1H, H-8), 5.79 (d, J_{H-H} = 3.92 Hz, 1H, H-1′), 5.68 (s, 1H, 2′-OH), 4.36 (m, 2H, H-3′, H-2′), 4.09-3.91 (m, 3H, H-5′, H-5″, H-4′), 3.14 (s, 3H, CH_3 dmm), 3.02 (s, 3H, CH_3 dmm), 0.99-1.04 (m, 28H, $(iPr)_4$); ^{13}C NMR (100 MHz, dmso-d_6), δ, ppm : 157.93, 157.54, 157.40, 149.32, 135.37, 119.65, 88.10, 80.95, 73.83, 69.67, 60.73, 17.27, 17.11, 17.08, 16.90, 16.84, 16.81, 16.74, 12.75, 12.40, 12.25, 11.98; HRMS (ESI): calcd for $C_{25}H_{44}N_6O_6Si_2$ $[M+H]^+$ 581.2934; found 581.2932;

N^6-benzoyl-3′,5′-O-(tetraisopropyldisiloxane-1,3-diyl)-adenosine (**2b**) ^{1}H NMR (400 MHz, dmso-d_6), δ, ppm: 11.24 (s, 1H, NH), 8.67 (s, 1H, H-2), 8.53 (s, 1H, H8), 8.06 - 7.53 (5H, H-

Ar), 6.01 (d, 1H, H-1′), 5.68 (d, 1H, 2′-OH), 4.84-4.81 (m, 1H, 2′), 4.65, (m, 1H, 3′) 4.09-3.93 (m, 3H, 4′, 5′, 5″), 1.06 - 0.94 (m, 28H, $(iPr)_4$); ^{13}C NMR (100 MHz, dmso-d_6), δ, ppm: 165.68, 151.41, 150.48, 143.07, 133.34, 132.43, 128.48, 128.43, 128.16, 127.41, 125.95, 89.52, 80.95, 73.38, 69.86, 60.72, 54.88, 17.32, 17.18, 17.17, 17.13, 17.01, 16.89, 16.81, 12.73, 12.42, 12.25, 12.05; HRMS (ESI): calcd for $C_{29}H_{43}N_5O_6Si_2$ $[M+H]^+$ 614.2825; found 614.2831;

N^2,N^6-diacetyl-3′,5′-O-(tetraisopropyldisiloxane-1,3-diyl)-2-aminoadenosine (**2c**) ^1H NMR (400 MHz, dmso-d_6): δ 10.56 (s, 1H, NH), 10.30 (s, 1H, NH), 8.32 (s, 1H, H-8), 5.89 (s, 1H, H-1′), 5.59 (s, 1H, 2′-OH), 4.55 - 4.50 (m, 2H, H-2′, H-3′), 4.09 - 3.92 (m, 3H, H-4′, H-5′, H-5″), 2.31 (s, 3H, Ac CH_3), 2.22 (s, 3H, Ac CH_3), 1.05-0.85 (m, $(iPr)_4$); ^{13}C NMR (176 MHz, dmso-d_6), δ, ppm: 169.88, 152.68, 152.40, 150.15, 141.30, 120.30, 89.09, 81.86, 73.87, 70.57, 61.51, 25.13, 24.98, 17.83, 17.67, 17.64, 17.57, 17.49, 17.41, 17.35, 13.18, 12.94, 12.68, 12.51; HRMS (ESI): calcd for $C_{26}H_{44}N_6O_7Si_2$ $[M-H]^-$ 607.2737; found 607.2747;

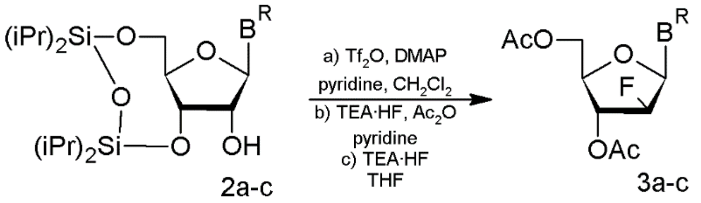

N-protected 3′,5′-di-O-acetyl-2′-deoxy-2′-fluoropurine arabinoside (step ii).

a) N-protected-3′,5′-O-(tetraisopropyldisiloxane-1,3-diyl)-purine riboside (**2**) (1 equiv) was dissolved in anhydrous dichloromethane (6 ml per 1 mmol) with the addition of pyridine (0.2 ml per 1 mmol) and 4-dimethylaminopyridine (DMAP) (5 equiv). The mixture was cooled in an ice bath for 30 min and trifluoromethanesulfonyl anhydride (7 equiv) was added dropwise over 15 min. Then, the reaction mixture was left at rt for 2 h to complete the reaction. The reaction mixture was worked up with an aqueous solution of sodium bicarbonate and extracted

3 times with dichloromethane. The organic layer was washed with an aqueous solution of dibasic sodium phosphate and the combined organic layers were dried with anhydrous sodium sulfate, filtered and evaporated.

b) Residue from previous reaction was dissolved in a 1 molar pyridine solution of triethylammonium fluoride (4 equiv), and acetic anhydride (8 equiv) was added. The mixture was left for 2 h at rt in a plastic container. After that time, the reaction was completed, and the reaction mixture was worked up with an aqueous solution of sodium bicarbonate and was extracted 3 times with dichloromethane. The combined organic layers were dried with anhydrous sodium sulfate, filtered and evaporated.

c) Prepared N-protected 2′-O-trifluoromethanesulfonyl-3′,5′-di-O-acetylpurine riboside without further purification was dissolved in anhydrous tetrahydrofuran (5 ml per 1 mmol), and a 1 molar pyridine solution of TEAHF (7 equiv) was added. The mixture was left for 64 h at 37° C. After that time, ^{19}F NMR signal at -74.57 ppm disappeared, and a new signal at -198.43 ppm was indicated. The reaction mixture was worked up with an aqueous solution of sodium bicarbonate and was extracted 3 times with dichloromethane. The combined organic layers were dried with anhydrous sodium sulfate, filtered and evaporated. Product (**3**) without purification was used for the next reaction.

3′,5′-di-O-acetyl-N^2-(dimethylaminomethylene)-2′-deoxy-2′-fluoroarabinoguanosine (**3a**). ^{1}H NMR (500 MHz, dmso-d$_6$), δ, ppm: 11.44 (s, 1H, NH), 8.62 (s, 1H, CH dmm), 7.87 (d, 1H, H-8), 6.34 (dd, 1H, H-1′, J_{H-F} = 15.18 Hz), 5.74 (m, 1H, H-2′, J_{H-F} = 19.30 Hz), 5.56 (m, 1H, H-3′, J_{H-F} = -51.20 Hz), 4.41-4.23 (m, 3H, H-5′, H-5″, H-4′), 3.18 (s, 3H, CH_3 dmm), 3.05 (s, 3H, CH_3 dmm), 2.15 (s, 3H, CH_3 Ac), 2.03 (s, 3H, CH_3 Ac); ^{13}C NMR (125 MHz, dmso-d$_6$), δ, ppm: 170.60, 170.32, 158.48, 157.98, 150.11, 138.12, 119.73, 93.76, 92.23, 81.89, 81.76, 77.84, 75.71, 75.49, 63.42, 41.09, 40.54-39.45, 35.18, 21.11, 20.93, 17.77, 13.08; ^{19}F NMR

(470 MHz, dmso-d$_6$), δ, ppm: -198.43 (ddd, 1F, $J_{H\text{-}F}$ = 17.36 Hz, $J_{H2'\text{-}F}$ = -51.58 Hz); HRMS (ESI): calcd for $C_{17}H_{21}FN_6O_6$ $[M+H]^+$ 425.1579; found 425.1573;

3',5'-di-O-acetyl-N^6-benzoyl-2'-deoxy-2'-fluoroarabinoadenosine (**3b**). ^{1}H NMR (500 MHz, dmso-d$_6$), δ, ppm: 11.27 (s, 1H, NH), 8.81 (s, 1H, H-2), 8.56 (s, 1H, H-8), 8.06-7.54 (5H, H-Ar), 6.64 (dd, 1H, H-1′, $J_{H\text{-}F}$ = 15.18 Hz), 5.71-5.57 (m, 2H), 4.44-4.33 (m, 3H), 2.16 (s, 3H, CH_3 Ac), 2.05 (s, 3H, CH_3 Ac); ^{13}C NMR (125 MHz, dmso-d$_6$), δ, ppm: 170.16, 169.63, 152.03, 151.91, 150.57, 143.24, 143.19, 133.24, 132.51, 128.51, 128.48, 93.68, 91.77, 81.96, 81.80, 78.17, 78.13, 75.41, 75.13, 63.00, 20.61, 20.52; ^{19}F NMR (470 MHz, dmso-d$_6$), δ, ppm: -197.90 (ddd, 1F, $J_{H\text{-}F}$ = 17.18 Hz, $J_{H2'\text{-}F}$ = -51.74 Hz); HRMS (ESI): calcd for $C_{21}H_{20}FN_5O_6$ $[M+H]^+$ 458.1470; found 458.1453;

N^2,N^6-diacetyl-3',5'-di-O-acetyl-2'-deoxy-2'-fluoro-2-aminoarabinoadenosine (**3c**). ^{1}H NMR (500 MHz, dmso-d$_6$), δ, ppm: 10.43 (s, 1H, NH), 8.36 (s, 1H, H8), 6.44 (dd, 1H, H-1′, $J_{H1'\text{-}F}$ = 16.73 Hz, $J_{H1'\text{-}H2'}$ = 3.96 Hz), 5.65-5.51 (m, 2H), 4.41-4.32 (m, 4H), 2.32 (s, 3H, NAc CH_3), 2.23 (s, 3H, NAc CH_3), 2.14 (s, 3H, OAc CH_3), 2.04 (s, 3H, OAc CH_3); ^{13}C NMR (125 MHz, dmso-d$_6$), δ, ppm: 170.65, 169.98, 169.92, 152.93, 152.79, 142.39, 119.51, 94.02, 92.49, 82.24, 82.10, 78.76, 75.93, 75.72, 63.61, 25.09, 25.06, 21.07, 21.00, 20.95, 20.81, 20.69, 19.00, 17.76, 13.08; ^{19}F NMR (470 MHz, dmso-d$_6$), δ, ppm: -197.75 (ddd, 1F, $J_{H\text{-}F}$ = 17.28 Hz, $J_{H2'\text{-}F}$ = -50.92 Hz); HRMS (ESI): calcd for $C_{18}H_{21}FN_6O_7$ $[M+H]^+$ 453.1529; found 453.1514;

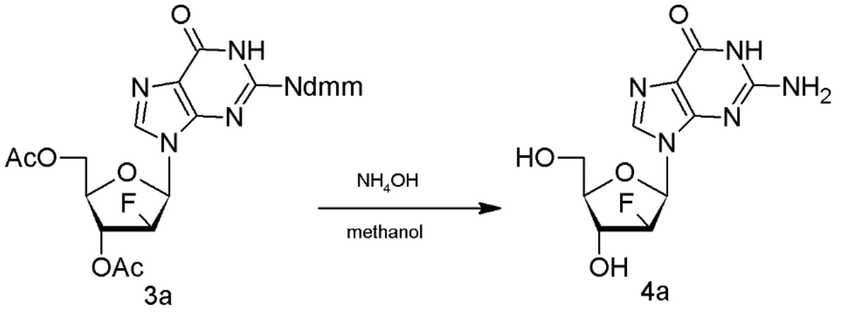

2′-deoxy-2′-fluoroarabinoguanosine (step iii). The residue from the previous reaction was dissolved in methanol (5 ml per 1 mmol) and 25 % aqueous ammonia (5 ml per 1 mmol) was added; the reaction was left in sealed vessel overnight at 55 °C. The mixture was cooled and evaporated twice with isopropanol to dryness. The yield for the two last steps was 60 %.

2′-deoxy-2′-fluoroarabinoguanosine (**4a**). ^{1}H NMR (500 MHz, dmso-d$_6$), δ, ppm: 10.94 (s, 1H, NH), 7.77 (s, 1H, H-8), 6.65 (s, 1H), 6.13 (dd, 1H, H-1′, $J_{H\text{-}F}$ = 16.19 Hz), 5.10 (m, 2H, H-2′, $J_{H2'\text{-}F}$ = -52.45 Hz, 2′-OH), 4.36 (dt, 1H, H-3′, $J_{H\text{-}F}$ = 17.87 Hz), 3.80 (m, 1H, H-4′), 3.40 (m, 2H, H-5′, H-5″); ^{13}C NMR (176 MHz, dmso-d$_6$), δ, ppm: 171.98, 157.10, 154.52, 151.31, 136.27, 136.24, 116.35, 96.12, 95.03, 84.14, 84.12, 81.73, 81.64, 73.18, 73.05, 60.90, 40.32-39.60, 22.97; ^{19}F NMR (376 MHz, dmso-d$_6$), δ, ppm: -198.09 (ddd, 1F, $J_{H\text{-}F}$ = 16.99 Hz, $J_{H2'\text{-}F}$ = -52.40 Hz); HRMS (ESI): calcd for $C_{10}H_{12}FN_5O_4$ $[M+H]^+$ 286.0946; found 286.0941;

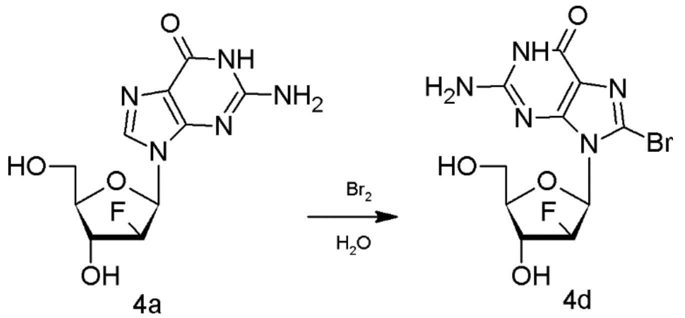

2′-deoxy-2′-fluoro-8-bromoarabinoguanosine (**4d**) (step iv). 2′-Deoxy-2′-fluoroarabinoguanosine (**4a**) was suspended in water and aqueous bromine was added dropwise until discoloration occurred. The product (**4d**), in the form of a white precipitate, was filtered and dried.

^{1}H NMR (500 MHz, dmso-d$_6$), δ, ppm: 10.83 (s, 1H, NH), 6.52 (s, 1H, 2′-OH), 6.27 (dd, 1H, H-1′, $J_{H\text{-}F}$ = 16.19 Hz), 5.23 (dt, 1H, H-2′, $J_{H2'\text{-}F}$ = -52.45 Hz), 4.68 (dt, 1H, H-3′), 3.78-3.71 (m, 3H, H-4′, H-5′, H-5″); ^{13}C NMR (125 MHz, dmso-d$_6$), δ, ppm: 155.45, 153.55, 152.35, 119.67, 116.97, 97.40, 95.45, 82.20, 82.11, 82.08, 81.90, 73.61, 73.40, 61.53; ^{19}F NMR (376 MHz,

dmso-d6), δ, ppm: -197.05 (dq, 1F, J_{H-F} = 10.06 Hz, J_{H-F} = 21.88 Hz, $J_{H2'-F}$ = -54.17 Hz); HRMS (ESI): calcd for $C_{10}H_{11}BrFN_5O_4$ $[M+H]^+$ 364.0051; found 364.0055

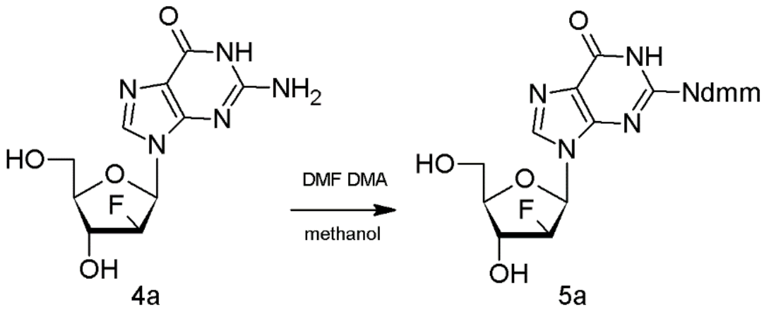

N^2-(dimethylaminomethylene)-2′-deoxy-2′-fluoroarabinoguanosine (**5a**) (step v). 2′-Deoxy-2′-fluoroarabinoguanosine (**4a**) was evaporated twice with methanol. The substrate was suspended in methanol (5 ml per 1 mmol) and N,N-dimethylformamide dimethyl acetal (DMF DMA) (5 equiv) was added and stirred for 6 h at 50 °C. Product (**5a**) was filtered, dried and used without further purification.

^{1}H NMR (500 MHz, dmso-d6), δ, ppm: 8.59 (s, 1H, CH dmm), 7.89 (d, 1H, H-8), 6.29 (dd, 1H, H-1′, J_{H-H} = 4.48 Hz, J_{H-F} = 15.69 Hz), 5.16 (dt, 1H, H-2′, $J_{H2'-F}$ = -52.55 Hz), 4.41 (dt, 1H, H-3′, J_{H-F} = 19.11 Hz), 4.09 (m, 1H, H-4′), 3.63 (m, 2H, H-5′, H5″), 3.16 (s, 3H, CH_3 dmm), 3.03 (s, 3H, CH_3 dmm); ^{13}C NMR (125 MHz, dmso-d6), δ, ppm: 162.80, 151.70, 135.23, 116.68, 96.50, 94.98, 83.98, 83.95, 81.65, 81.52, 73.40, 73.21, 61.01, 36.25, 31.23; ^{19}F NMR (376 MHz, dmso-d6), δ, ppm: -197.27 (ddd, 1F, J_{H-F} = 17.26 Hz, $J_{H2'-F}$ = -52.45 Hz); HRMS (ESI): calcd for $C_{13}H_{17}FN_6O_4$ $[M+H]^+$ 341.1368; found 341.1366

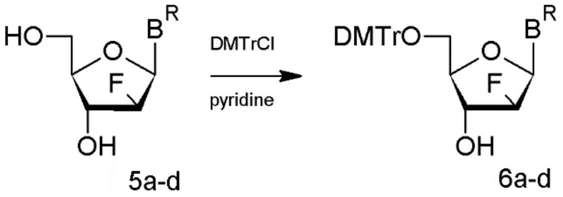

N-protected 5′-O-(4,4′-dimethoxytrityl)-2′-deoxy-2′-fluoropurine arabinoside (step vi). Base-protected 2′-deoxy-2′-fluoropurine arabinoside (**5**) was evaporated twice with anhydrous pyridine and dissolved in pyridine (5 ml per 1 mmol). The dimethoxytrityl chloride (1.1 equiv) was added and the mixture was stirred at rt for 3 h. After reaction completion, the reaction mixture was worked up with an aqueous solution of sodium bicarbonate and extracted 3 times with dichloromethane. The combined organic layers were dried with anhydrous sodium sulfate, filtered and evaporated. Product (**6**) was purified by silica gel column chromatography using dichloromethane and methanol (linear gradient up to 7 %) as eluent. Yield: ca. 80 %.

5′-O-(4,4′-dimethoxytrityl)-N^2-(dimethylaminomethylene)-2′-deoxy-2′-fluoroarabinoguanosine (**6a**). ^{1}H NMR (500 MHz, dmso-d_6), δ, ppm: 11.37 (s, 1H, NH), 8.53 (s, 1H, CH dmm), 7.92 (d, 1H, H-8), 7.74-6.83 (Ar-DMT), 6.84 (dd, 1H, H-1′, $J_{H1'-F}$ = 16.39 Hz, $J_{H1'-H2'}$ = 4.38 Hz), 6.12 (s, 1H, 3′-OH), 5.19 (dt, 1H, H-2′, $J_{H2'-F}$ = -52.19 Hz), 4.43 (m, 1H, H-3′), 3.99 (m, 1H, H-4′), 3.71 (s, 6H, CH_3-DMT), 3.32-3.20 (m, 2H, H-5′, H-5″), 3.10 (s, 3H, CH_3 dmm), 3.04 (s, 3H, CH_3 dmm); ^{13}C NMR (125 MHz, dmso-d_6), δ, ppm: 162.98, 158.55, 158.13, 158.03, 150.25, 145.15, 137.61, 137.57, 135.91, 135.84, 130.13, 128.30, 128.09, 127.24, 119.28, 113.61, 96.43, 94.90, 85.97, 82.15, 82.11, 81.61, 81.48, 74.29, 74.10, 63.84, 55.48, 41.15, 40.22, 40.05, 39.97, 39.88, 39.80, 39.71, 39.55, 39.38, 39.21, 36.30, 35.15, 31.27; ^{19}F NMR (470 MHz, dmso-d_6), δ, ppm: -197.28 (ddd, 1F, J_{H-F} = 17.52 Hz, $J_{F-H2'}$ = -53.34 Hz); HRMS (ESI): calcd for $C_{34}H_{35}FN_6O_6$ $[M+H]^+$ 643.2675; found 643.2676

N^6-benzoyl-5′-O-(4,4′-dimethoxytrityl)-2′-deoxy-2′-fluoroarabinoadenosine (**6b**). ^{1}H NMR (500 MHz dmso-d_6), δ, ppm: 11.27 (s, 1H, NH), 8.03-6.83 (m, Bz-Ar, DMT-Ar), 6.61 (dd, 1H, $J_{H1'-F}$ = 13.9 Hz, $J_{H1'-H2'}$ = 4.74 Hz), 6.04 (d, 3′-OH), 5.32 (dt, 1H, H-2′, $J_{H2'-F}$ = -52.44 Hz), 4.57-4.51 (m, 1H, H-3′), 4.07 (m, 1H, H-4′), 3.72 (s, 6H, CH_3-DMT), 3.41-3.37, 3.28-3.26 (m, 2H, H-5′, H-5″); ^{13}C NMR (125 MHz dmso-d_6), δ, ppm: 167.90, 165.65, 158.10, 151.92, 150.50, 144.74, 142.90, 135.53, 135.39, 134.25, 133.30, 132.48, 131.19, 129.71, 129.69, 128.51,

128.47, 128.30, 128.18, 127.81, 127.68, 127.44, 126.72, 125.08, 113.18, 96.33, 94.42, 85.55, 81.74, 81.66, 81.49, 73.69, 73.45, 63.38, 55.01, 39.50; ^{19}F NMR (470 MHz, dmso-d_6), δ, ppm: -197.68 (ddd, 1F, $J_{H\text{-}F}$ = 17.11 Hz, $J_{H2'\text{-}F}$ = -53.14 Hz); HRMS (ESI): calcd for $C_{38}H_{34}FN_5O_6$ $[M+H]^+$ 676.2566; found 676.2572

N^2,N^6-diacetyl-5'-O-(4,4'-dimethoxytrityl)-2'-deoxy-2'-fluoro-2-aminoarabinoadenosine (**6c**). ^{1}H NMR (400 MHz, dmso-d_6), δ, ppm: 10.60, 10.35 (2xs, 2H, NH), 8.22 (1H, H-8), 7.38 - 6.79 (m, 9H, Ar-DMT), 6.43 (dd, 1H, H-1′, $J_{H1'\text{-}F}$ = 13.36 Hz, $J_{H1'\text{-}H2'}$ = 4.84 Hz), 5.96 (d, 1H, 3′-OH), 5.27 (dt, 1H, H-2′, $J_{H\text{-}F}$ = -52.47 Hz), 4.65 (m, 1H, H-3′), 4.04 (m, 1H, H-4′), 3.72 (s, 6H, CH_3-DMT), 3.48-3.44, 3.24-3.21 (m, 2H, H-5′, H-5″), 2.32 (s, 3H, Ac CH_3), 2.20 (s, 3H, Ac CH_3); ^{13}C NMR (100 MHz, dmso-d_6), δ, ppm: 162.79, 158.35, 157.93, 157.83, 150.06, 144.96, 135.71, 135.65, 129.93, 128.10, 127.90, 127.04, 119.08, 113.42, 96.23, 94.71, 85.77, 81.95, 81.91, 81.42, 81.28, 74.09, 73.90, 63.64, 55.28, 40.95 - 39.02, 36.11, 34.95, 31.07; ^{19}F NMR (376 MHz, dmso-d_6): δ -198.05 (ddd, 1F, $J_{F\text{-}H2'}$ = = -52.10 Hz, $J_{F\text{-}H}$ = 16.09 Hz); HRMS (ESI): calcd for $C_{35}H_{35}FN_6O_7$ $[M+H]^+$ 671.2624; found 671.2634;

5'-O-(4,4'-dimethoxytrityl)-N^2-(dimethylaminomethylene)-2'-deoxy-2'-fluoro-8-bromoarabinoguanosine (**6d**). ^{1}H NMR (400 MHz, dmso-d_6), δ, ppm: 11.54 (s, 1H, NH), 8.23 (s, 1H, CH dmm), 7.35 - 6.77 (m, Ar-DMT), 6.48 (dd, 1H, H-1′, $J_{H\text{-}F}$ = 10.42 Hz, $J_{H1'\text{-}H2'}$ = 6.38 Hz), 5.97 (s, 1H, 3′-OH), 5.34 (dt, 1H, H-2′), 4.72-4.65 (m, 1H, H-3′), 3.96 (m, 1H, H-4′), 3.72 (s, 6H, CH_3-DMT), 3.55 (m, 1H, H-5″), 3.24-3.17 (m, 1H, H-5′), 3.00 (s, 3H, CH_3 dmm), 2.98 (s, 3H, CH_3 dmm); ^{13}C NMR (100 MHz, dmso-d_6), δ, ppm: 158.50, 158.43, 157.97, 157.52, 156.80, 151.49, 145.23, 135.89, 135.83, 130.13, 130.01, 128.17, 128.12, 127.09, 121.83, 120.46, 113.53, 113.49, 97.39, 95.83, 85.94, 82.23, 82.09, 79.89, 79.80, 74.52, 74.35, 64.21, 55.48, 55.45, 49.07, 41.24, 40.57-39.47, 35.12; ^{19}F NMR (376 MHz, dmso-d6): δ -197.31 (ddd, 1F, $J_{F\text{-}H2'}$ = -52.28 Hz, $J_{F\text{-}H}$ = 17.52 Hz); HRMS (ESI): calcd for $C_{34}H_{34Br}FN_6O_6$ $[M+H]^+$ 721.1780; found 721.1773;

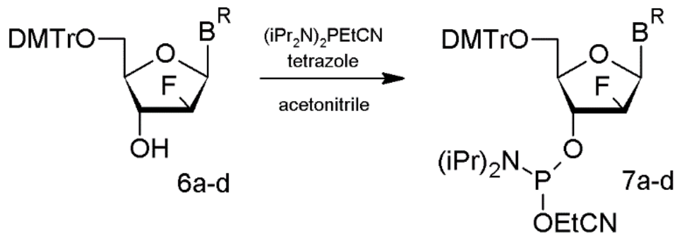

N-protected [5′-O-(dimethoxytrityl)-2′-deoxy-2′-fluoroarabinofuranosyl]purine 3′-O-(2-cyanoethyl N,N-diisopropyl)phosphoramidite (step vii). Dimethoxytrityl and base-protected purine riboside (**6**) (1 equiv) and tetrazole (1 equiv) was vacuum-dried for 8 h. Substrates were dissolved in anhydrous acetonitrile (5 ml per 1 mmol) and 2-cyanoethyl N,N,N′,N′-tetraisopropylphosphorodiamidite (1.1 equiv) was added. The mixture was stirred at rt overnight. The reaction mixture after completion was worked up with an aqueous solution of sodium bicarbonate and extracted 3 times with dichloromethane containing 1 % of triethylamine (TEA). The combined organic layers were dried with sodium sulfate and evaporated. Product was purified using silica gel column chromatography starting with hexane, and ethyl acetate was used to form a gradient. During purification, the eluent contained 1 % TEA. Product (**7**) was lyophilized from benzene. Yield: 92 % (after purification).

5′-O-(dimethoxytrityl)-N²-(dimethylaminomethylene)-2′-deoxy-2′-fluoroarabinoguanosine 3′-O-(2-cyanoethyl N,N-diisopropyl)phosphoramidite (**7a**). ^{1}H NMR (400 MHz, CD_3CN-d_3), δ, ppm: 9.49 (s, 1H, NH), 8.60 (s, 1H, CH dmm), 7.70 (s, 1H, H-8), 7.47-6.84 (13H, Ar-DMT), 6.34 (dd, 1H, H-1′), 5.23 (m, 1H, H-2′), 4.80-4.65 (m, 1H, H-3′), 4.15 (m, 1H, H-4′),3.76 (s, 6H, CH_3-DMT), 3.67-3.60 (m, 4H, O-CH_2 CEO, 2x CH iPr), 3.40-3.36 (m, 2H, H-5′, 5″), 3.10 (s, 3H, CH_3 dmm), 3.05 (s, 3H, CH_3 dmm), 2.62 (t, 1H, CH_2 CEO), 2.52 (t, 1H, CH_2 CEO), 1.18-1.07 (13H, CH_3 iPr); ^{13}C NMR (100 MHz, CD_3CN-d_3), δ, ppm: 159.68, 159.33, 159.26, 158.78, 158.66, 158.62, 151.26, 151.24, 145.95, 145.92, 138.10, 138.06, 138.04, 138.01, 136.73, 136.68, 131.00, 130.96, 130.94, 129.27, 128.97, 128.90, 128.83, 127.87, 127.84, 120.43, 120.36, 119.39, 119.28, 118.27, 114.06, 96.67, 96.63, 96.61, 96.59, 94.75, 94.71, 94.69,

94.67, 87.13, 87.10, 83.24, 83.10, 83.07, 82.99, 82.93, 82.90, 82.82, 82.78, 82.74, 77.77, 77.60, 77.51, 77.34, 77.00, 76.82, 76.74, 76.57, 63.94, 63.72, 59.94, 59.77, 59.75, 59.58, 59.16, 59.11, 55.89, 55.88, 45.99, 45.92, 44.27, 44.20, 44.15,44.07, 41.65, 35.30, 24.94, 24.90, 24.87, 24.83, 24.80, 24.73, 23.16, 23.14, 23.09, 23.07, 20.99, 20.94, 20.92, 20.87, 20.61, 20.53, 1.30; ^{19}F NMR (470 MHz, CD_3CN-d_3), δ, ppm: -197.17 (dddd, 1F, $J_{H2'-F}$ = -52.46 Hz, J_{H-F} = 18.83 Hz, J_{P-F} = 78,49 Hz); ^{31}P NMR (162 MHz CD_3CN-d_3): δ, ppm: 151.27, 150.82; HRMS (ESI): calcd for $C_{43}H_{52}FN_8O_7P$ [M+H]$^+$ 843.3753; found 843.3741;

5′-O-(dimethoxytrityl)-N^6-benzoyl-2′-deoxy-2′-fluoroarabinoadenosine 3′-O-(2-cyanoethyl N,N-diisopropyl)phosphoramidite (**7b**). ^{1}H NMR (500 MHz, CD_3CN-d_3), δ, ppm: 9.51 (s, 1H, NH), 8.67 - 8.66 (1H, H-2), 8.29-8.27 (1H, H-8), 8.03-6.85 (18H, Bz-Ar, DMT-Ar), 6.61-6.57 (m, 1H, H-1′), 5.47 – 5.32 (m, 1H, H-2′), 4.93-4.82 (m, 1H, H-3′), 4.27-4.25 (m, 1H, H-4′), 4.14-3.63 (m, 4H, H-5′, H-5″), 3.77 (s, 6H, CH_3-DMT), 2.66 (t, 1H, CH_2 CEO), 2.56 (t, 1H, CH_2 CEO), 1.26-1.07 (13H, CH_3 iPr); ^{13}C NMR (125 MHz, CD_3CN-d_3), δ, ppm: 159.29, 159.28, 145.53, 145.51, 143.19, 136.32, 136.30, 136.27, 133.14, 130.60, 129.22, 128.73, 128.42, 117.89, 113.64,96.22, 96.19, 96.07, 94.68, 94.65, 94.53, 86.76, 83.15, 83.02, 82.93, 82.79, 82.70, 82.49, 77.11, 76.98, 76.91, 76.78, 76.28, 76.15, 76.08, 75.95, 63.49, 63.28, 59.47, 59.35, 59.32, 59.19, 55.48, 55.47, 45.58, 45.53, 43.83, 43.77, 43.74, 43.67, 24.51, 24.44, 24.42, 24.39, 24.36, 24.33, 22.76, 22.74, 22.69, 22.67, 20.57, 20.52, 20.47, 1.39, 1.23, 1.06-0.40; ^{19}F NMR (470 MHz, CD_3CN-d_3), δ, ppm: -197.12 (dddd, 1F, $J_{H2'-F}$ = -51.78 Hz, J_{H-F} = 17.52 Hz, J_{P-F} = 96,71 Hz); ^{31}P NMR (202 MHz, CD_3CN-d_3), δ, ppm: 151.03, 150.95; HRMS (ESI): calcd for $C_{43}H_{52}FN_8O_7P$ [M+H]$^+$ 876.3655; found 876.3641;

N^2,N^6-diacetyl-5′-O-(dimethoxytrityl)-2′-deoxy-2′-fluoro-2-aminoarabinoadenosine 3′-O-(2-cyanoethyl N,N-diisopropyl)phosphoramidite (**7c**). ^{1}H NMR (400 MHz, CD_3CN-d_3), δ, ppm: 8.74, 8.64 (2xs, 2H, NH), 8.07 (m, 1H, H-8), 7.43 - 6.78 (14H, Ar-DMT), 6.41-6.36 (m, 1H, H-1′), 5.43-5.25 (m, 1H, H-2′), 5.01-4.83 (m, 1H, H-3′), 4.19 (m, 1H, H-4′), 4.14-3.80 (m, 2H,

H-5′, H-5″), 3.74 (s, 6H, CH_3-DMT), 3.68-3.37 (m, 4H, O-CH_2 CEO, 2x CH iPr), 3.37 (m, 2H, H-5′, 5″), 2.75 (t, 1H, CH_2 CEO), 2.63 (t, 1H, CH_2 CEO), 2.31 (1H, CH_3 Ac), 2.16 (1H, CH_3 Ac), 1.24-1.07 (12H, CH_3 iPr); ^{13}C NMR (100 MHz, CD_3CN-d_3), δ, ppm: 170.98, 159.64, 159.62, 159.60, 153.03, 150.62, 145.97, 136.79, 136.71, 136.69, 136.65, 131.05, 131.00, 130.94, 128.99, 128.91, 128.72, 127.79, 119.28, 118.25, 113.93, 87.06, 83.59, 83.42, 83.27, 83.19, 83.10, 64.12, 59.74, 59.65, 59.55, 59.46, 59.12, 59.07, 55.84, 45.91, 44.24, 44.19, 44.11, 44.07, 25.79, 25.29, 24.89, 24.84, 24.81, 24.77, 24.71, 24.68, 23.14, 23.12, 23.06, 23.05, 20.87, 1.89, 1.68, 1.48, 1.27, 1.06, 0.86, 0.65; ^{19}F NMR (376 MHz, CD_3CN-d_3), δ, ppm: -197.79 - -198.16 (m); ^{31}P NMR (162 MHz, CD_3CN-d_3), δ, ppm: 151.12, 150.96; HRMS (ESI): calcd for $C_{43}H_{52}FN_8O_7P$ [M+H]$^+$ 871.3703; found 871.3711;

5′-O-(dimethoxytrityl)-N^2-(dimethylaminomethylene)-2′-deoxy-2′-fluoro-8-bromoarabinoguanosine 3′-O-(2-cyanoethyl N,N-diisopropyl)phosphoramidite (**7d**). ^{1}H NMR (500 MHz CD_3CN-d_3), δ, ppm: 9.77 (s, 1H, NH), 8.28, 8.25 (2xs, 1H, H-8), 7.41-6.72 (14H, Ar-DMT), 6.52-6.47 (m, 1H, H-1′), 5.51-5.35 (m, 1H, H-2′), 5.25-5.02 (m, 1H, H-3′), 4.15 (m, 1H, H-4′),3.75 (s, 6H, CH_3-DMT), 3.72-3.50 (m, 4H, O-CH_2 CEO, 2x CH iPr), 3.37 (m, 2H, H-5′, 5″), 3.09 (s, 3H, CH_3 dmm), 3.04 (s, 3H, CH_3 dmm), 2.62 (t, 1H, CH_2 CEO), 2.52 (t, 1H, CH_2 CEO), 1.18-1.07 (12H, CH_3 iPr); ^{13}C NMR (400 MHz, CD_3CN-d_3), δ, ppm: 159.61, 159.54, 158.58, 158.51, 158.10, 158.03, 157.49, 152.53, 152.48, 146.01, 145.91, 136.70, 136.57, 136.53, 136.50, 130.99, 130.93, 130.77, 129.27, 128.90, 128.79, 128.77, 128.74, 127.79, 127.73, 122.56, 121.58, 119.32, 119.18, 118.27, 113.99, 113.97, 96.83, 95.25, 87.06, 87.03, 83.65, 83.50, 83.40, 83.26, 80.18, 80.14, 80.10, 80.06, 77.33, 77.16, 76.98, 64.61, 64.31, 59.99, 59.85, 59.56, 59.41, 55.89, 55.86, 44.35, 44.26, 44.18, 44.08, 41.81, 41.77, 35.35, 24.88, 24.82, 24.53, 24.47, 20.88, 20.82, 1.79, 1.62, 1.46, 1.29, 1.13, 0.96, 0.80 ; ^{19}F NMR (470 MHz, CD_3CN-d_3), δ, ppm: -197.77 - -197.97 (m); ^{31}P NMR (202 MHz, CD_3CN-d_3), δ, ppm: 152.21, 151.22; HRMS (ESI): calcd for [M+H]$^+$ 921.2858; found 921.2865;

S4. NMR Spectra.

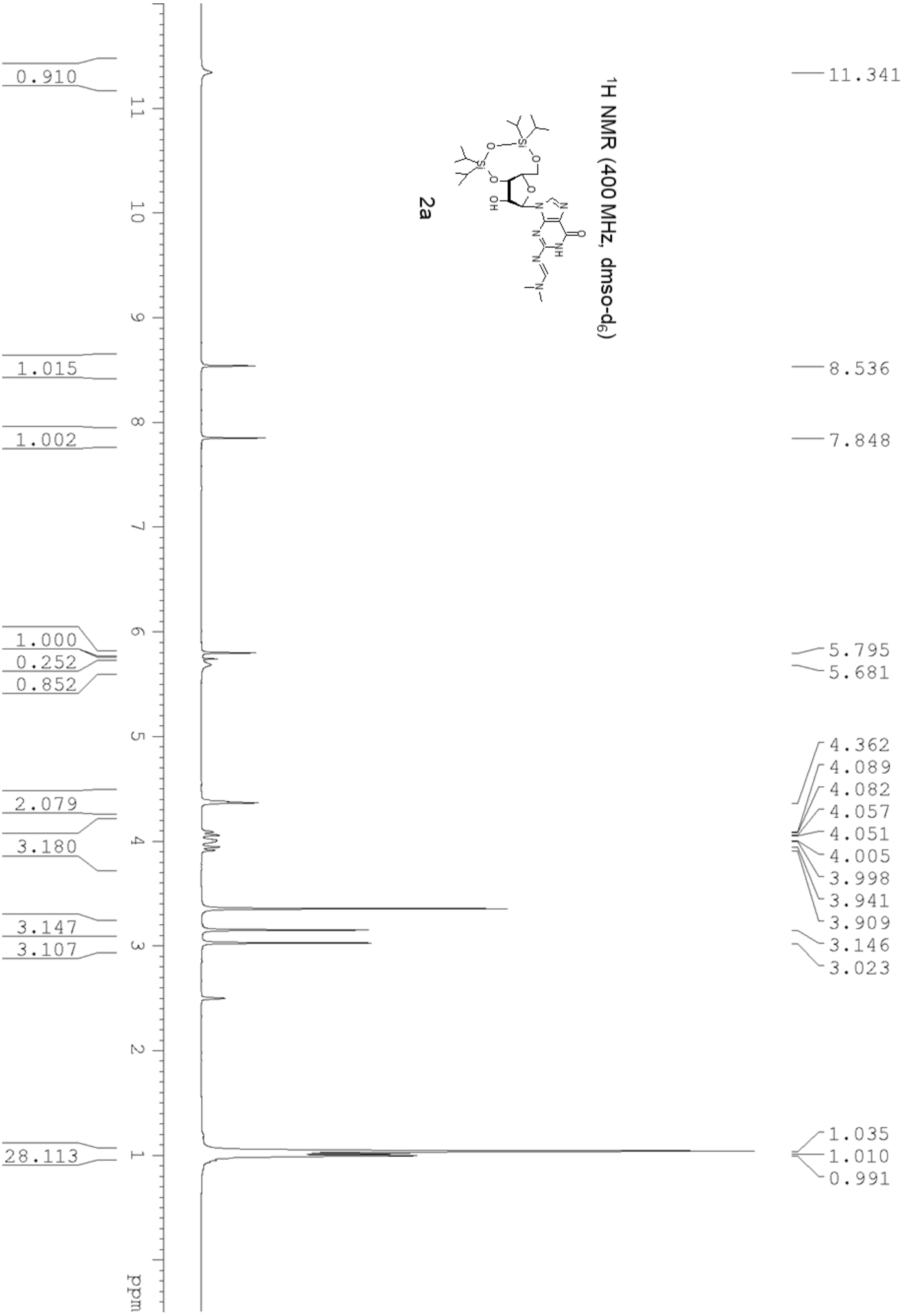

^{13}C NMR (100 MHz, dmso-d_6)

2a

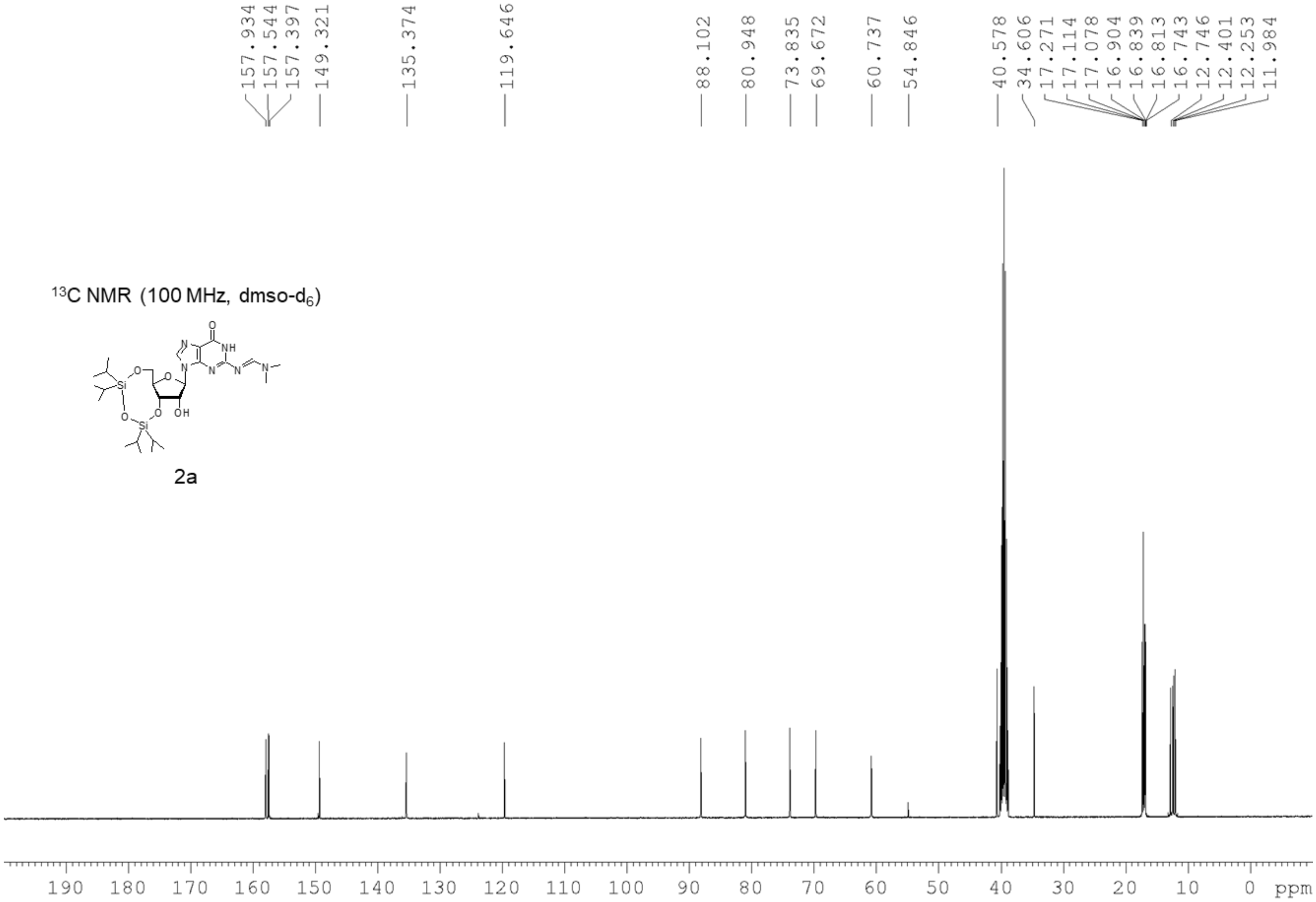

^1H NMR (400 MHz, dmso-d_6)

2b

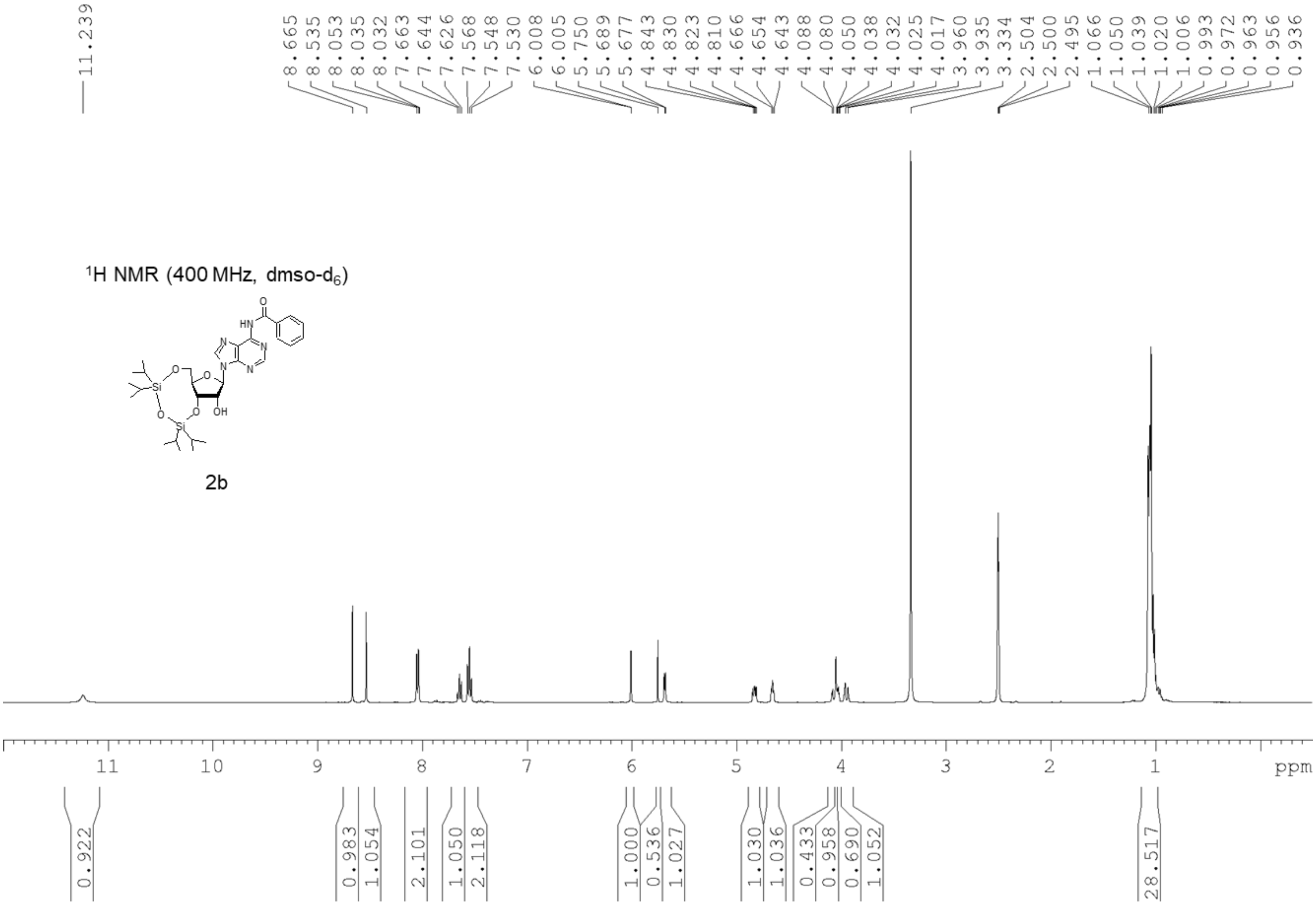

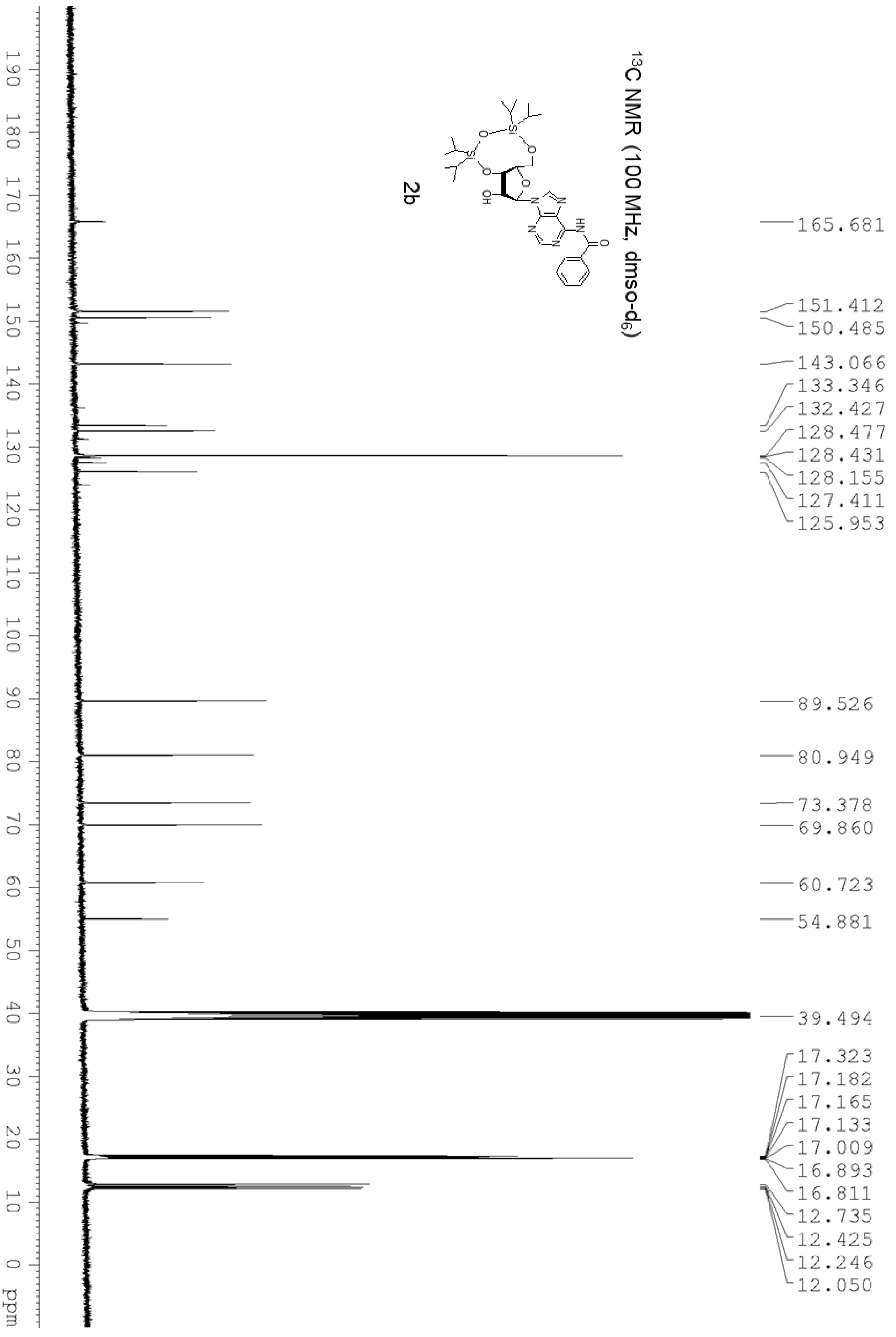
13C NMR (100 MHz, dmso-d6)
2b
165.681
151.412
150.485
143.066
133.346
132.427
128.477
128.431
128.155
127.411
125.953
89.526
80.949
73.378
69.860
60.723
54.881
39.494
17.323
17.182
17.165
17.133
17.009
16.893
16.811
12.735
12.425
12.246
12.050
190 180 170 160 150 140 130 120 110 100 90 80 70 60 50 40 30 20 10 0 ppm

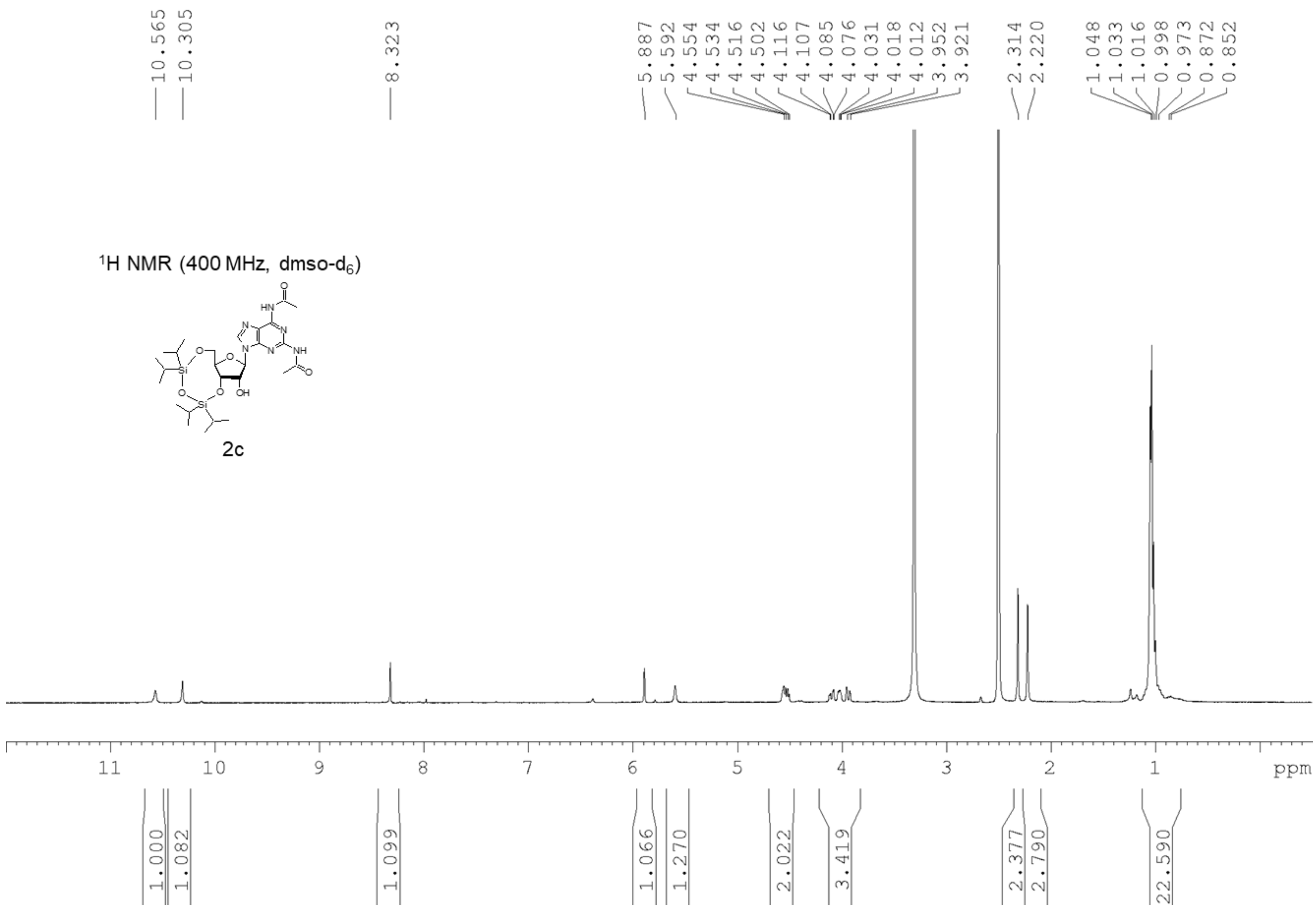

1H NMR (400 MHz, dmso-d6)
2c
10.565
10.305
8.323
5.887
5.592
4.554
4.534
4.516
4.502
4.116
4.107
4.085
4.076
4.031
4.018
4.012
3.952
3.921
2.314
2.220
1.048
1.033
1.016
0.998
0.973
0.872
0.852
11
10
9
8
7
6
5
4
3
2
1
ppm
1.000
1.082
1.099
1.066
1.270
2.022
3.419
2.377
2.790
22.590

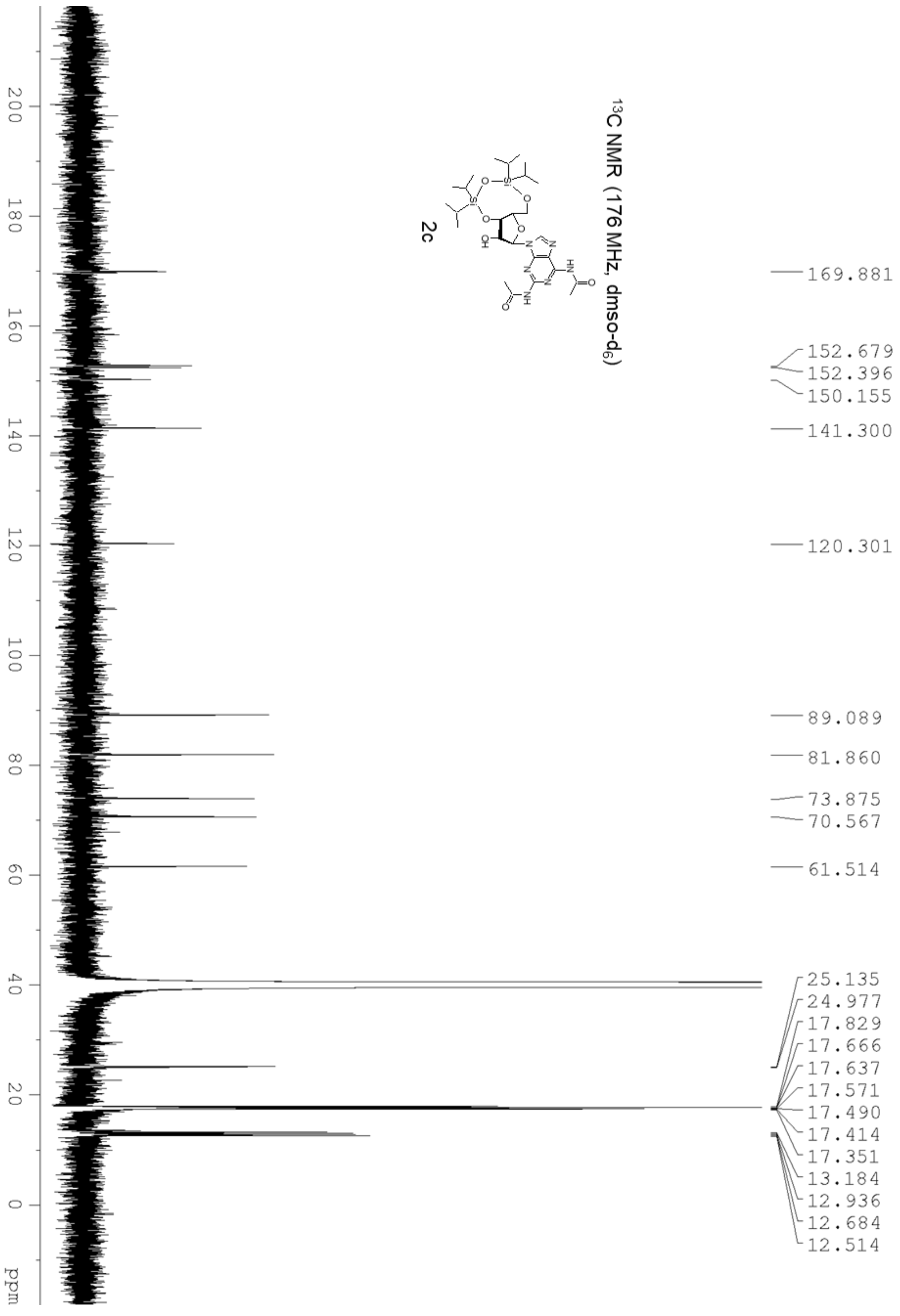
13C NMR (176 MHz, dmso-d6)
2c
169.881
152.679
152.396
150.155
141.300
120.301
89.089
81.860
73.875
70.567
61.514
25.135
24.977
17.829
17.666
17.637
17.571
17.490
17.414
17.351
13.184
12.936
12.684
12.514
200
180
160
140
120
100
80
60
40
20
0
ppm

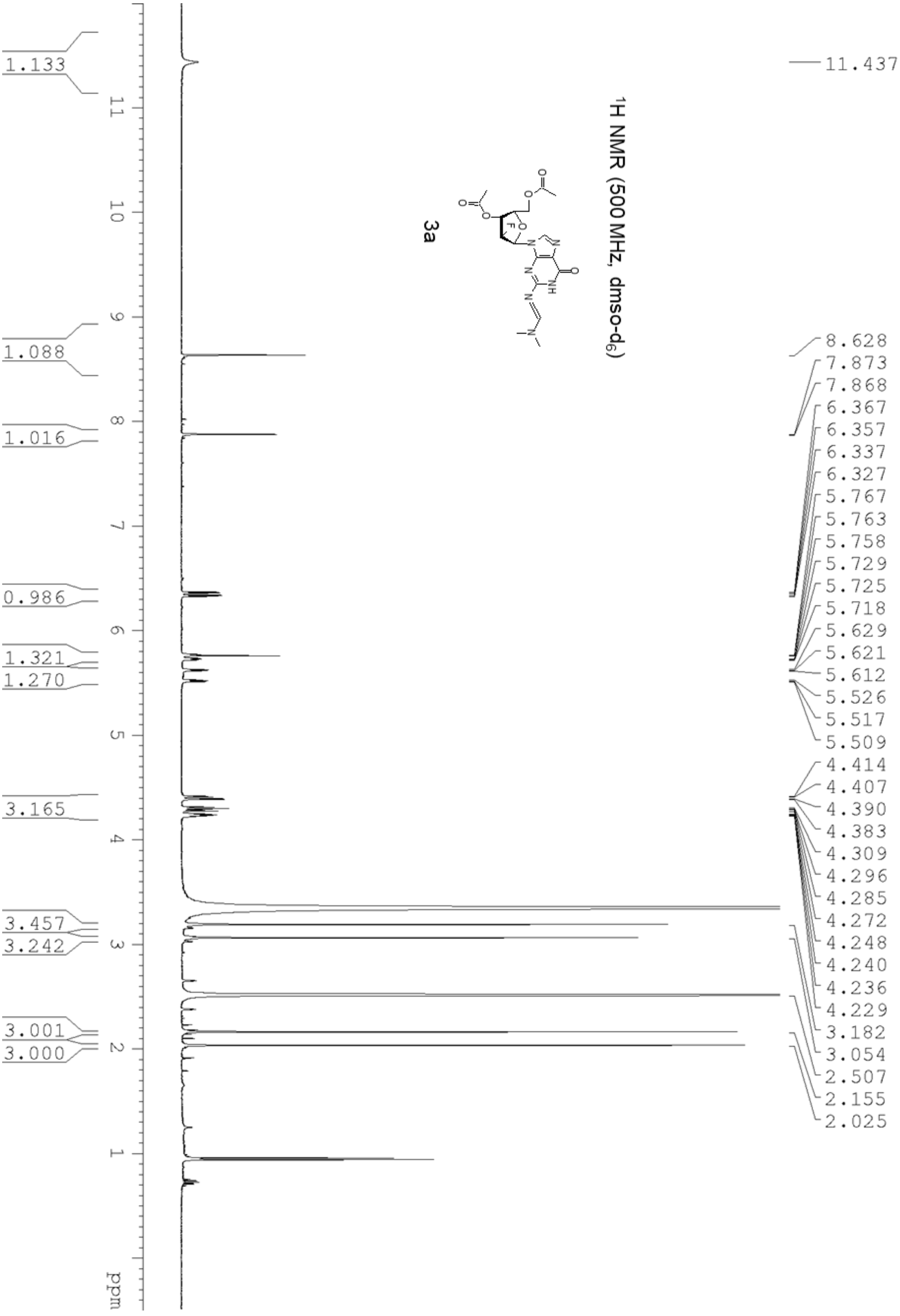
1H NMR (500 MHz, dmso-d6)
3a
11.437
8.628
7.873
7.868
6.367
6.357
6.337
6.327
5.767
5.763
5.758
5.729
5.725
5.718
5.629
5.621
5.612
5.526
5.517
5.509
4.414
4.407
4.390
4.383
4.309
4.296
4.285
4.272
4.248
4.240
4.236
4.229
3.182
3.054
2.507
2.155
2.025
1.133
1.088
1.016
0.986
1.321
1.270
3.165
3.457
3.242
3.001
3.000
ppm

^{13}C NMR (125 MHz, dmso-d$_6$)

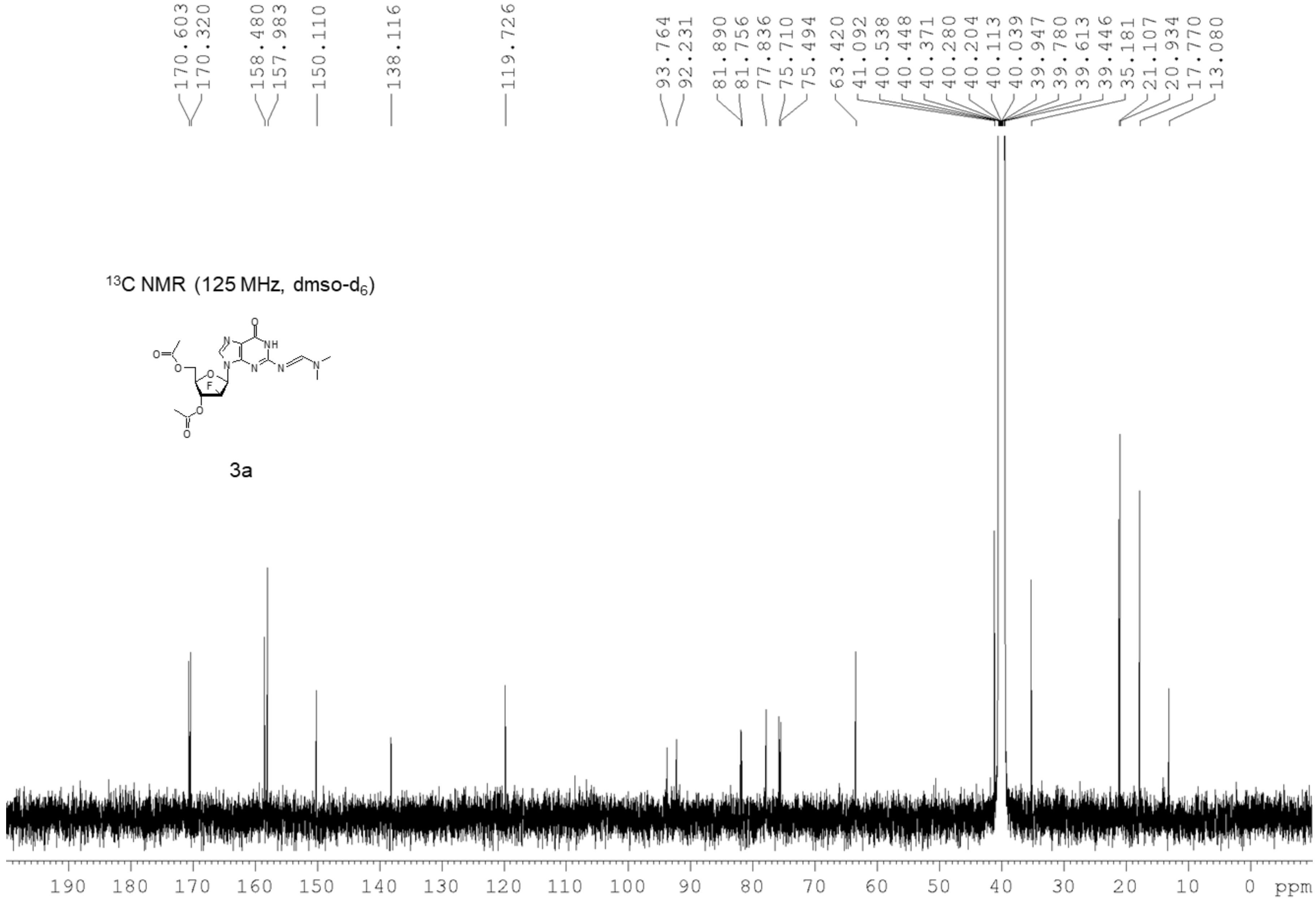

[1]F NMR (470 MHz, dmso-d$_6$)

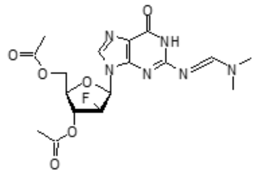

3a

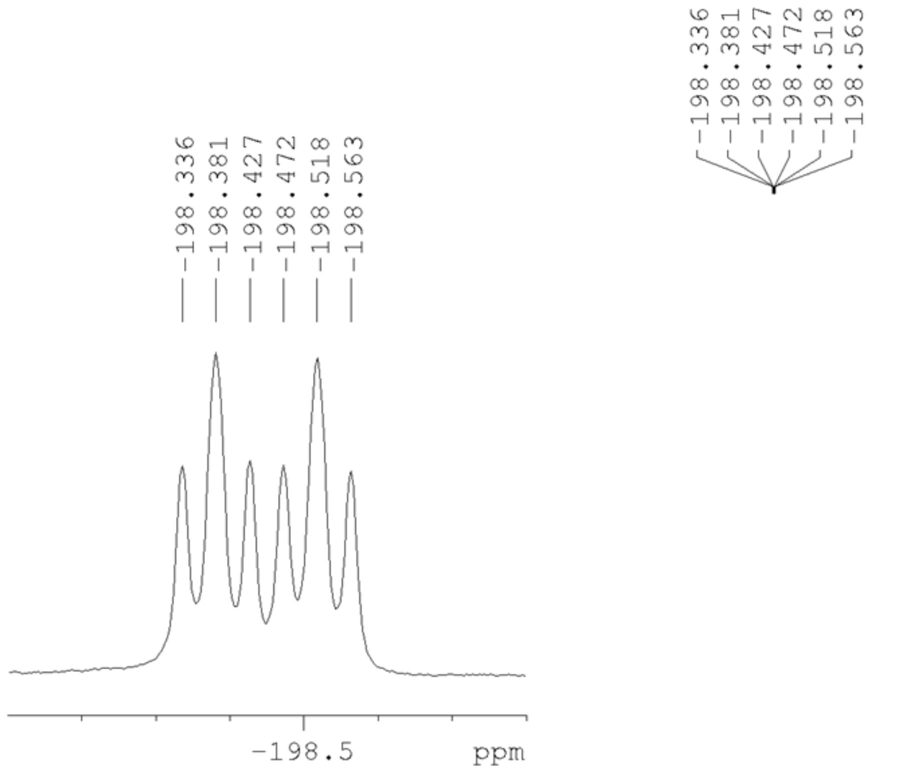

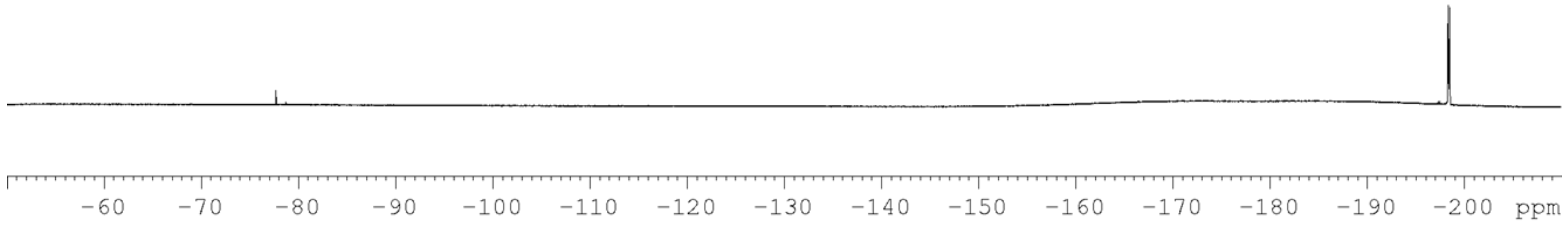

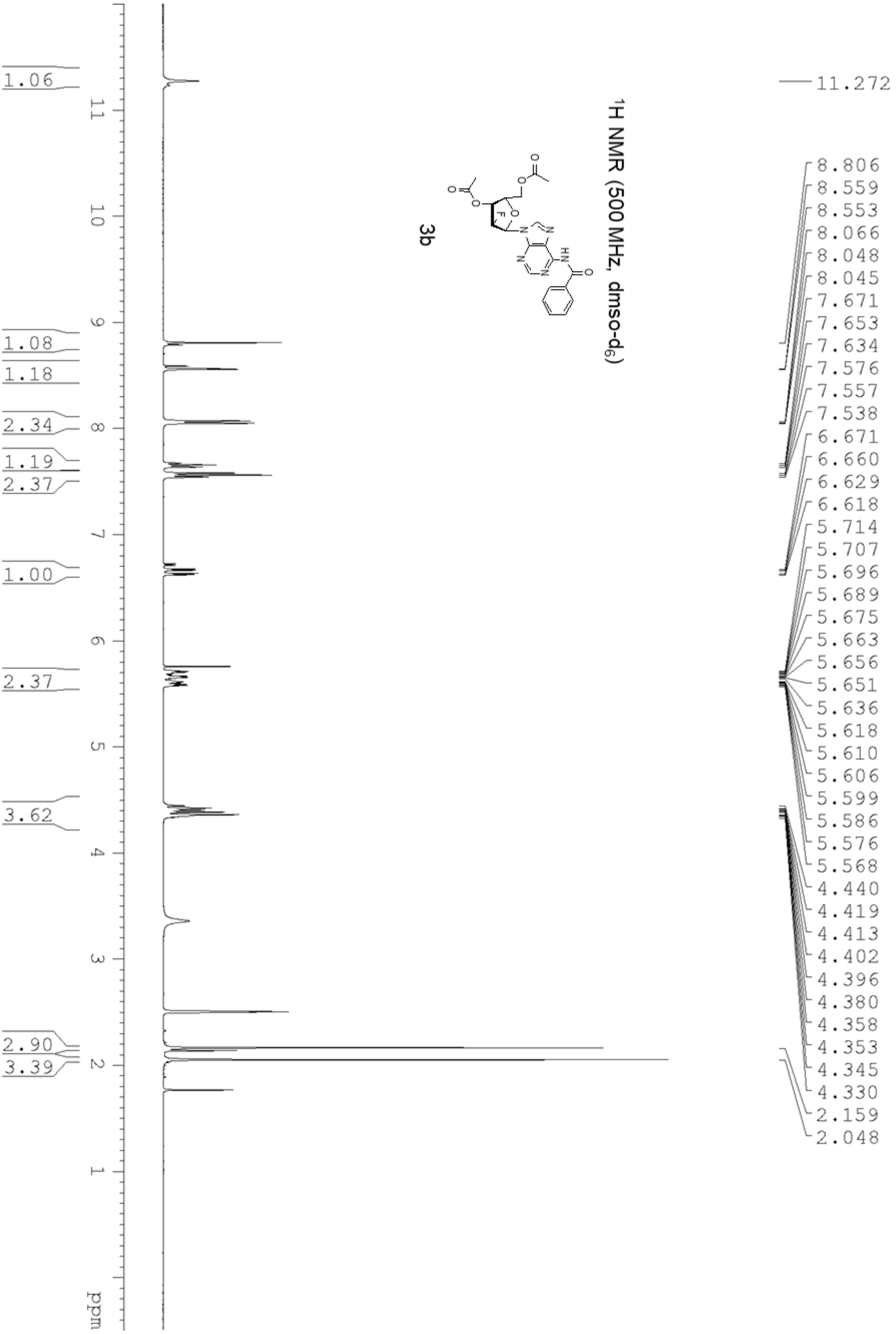

¹H NMR (500 MHz, dmso-d6)
3b
11.272
8.806
8.559
8.553
8.066
8.048
8.045
7.671
7.653
7.634
7.576
7.557
7.538
6.671
6.660
6.629
6.618
5.714
5.707
5.696
5.689
5.675
5.663
5.656
5.651
5.636
5.618
5.610
5.606
5.599
5.586
5.576
5.568
4.440
4.419
4.413
4.402
4.396
4.380
4.358
4.353
4.345
4.330
2.159
2.048
1.06
1.08
1.18
2.34
1.19
2.37
1.00
2.37
3.62
2.90
3.39
ppm

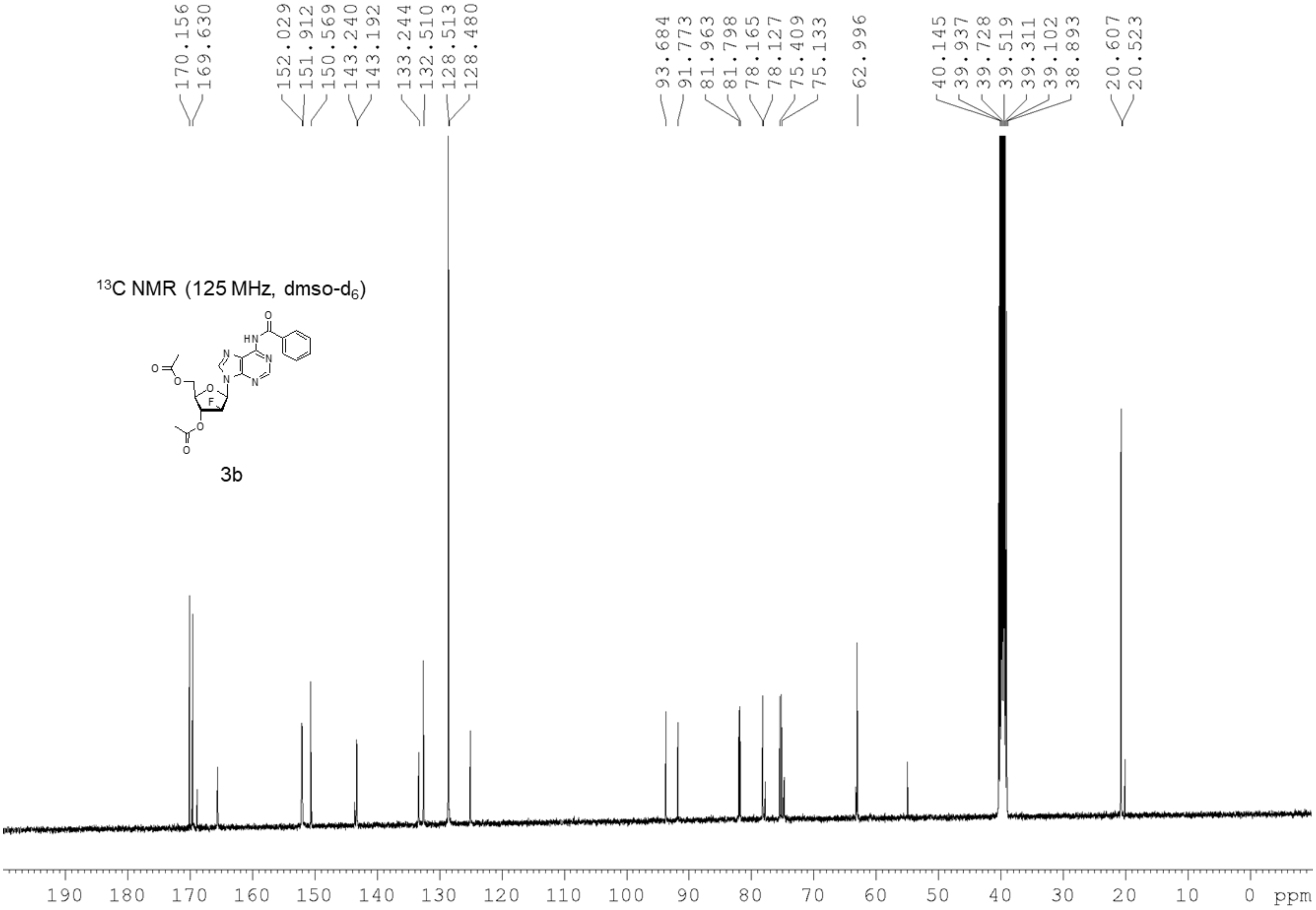

^{13}C NMR (125 MHz, dmso-d_6)
170.156
169.630
152.029
151.912
150.569
143.240
143.192
133.244
132.510
128.513
128.480
93.684
91.773
81.963
81.798
78.165
78.127
75.409
75.133
62.996
40.145
39.937
39.728
39.519
39.311
39.102
38.893
20.607
20.523
190 180 170 160 150 140 130 120 110 100 90 80 70 60 50 40 30 20 10 0 ppm

3b

^{19}F NMR (470MHz, dmso-d_6)

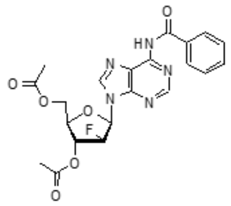

3b

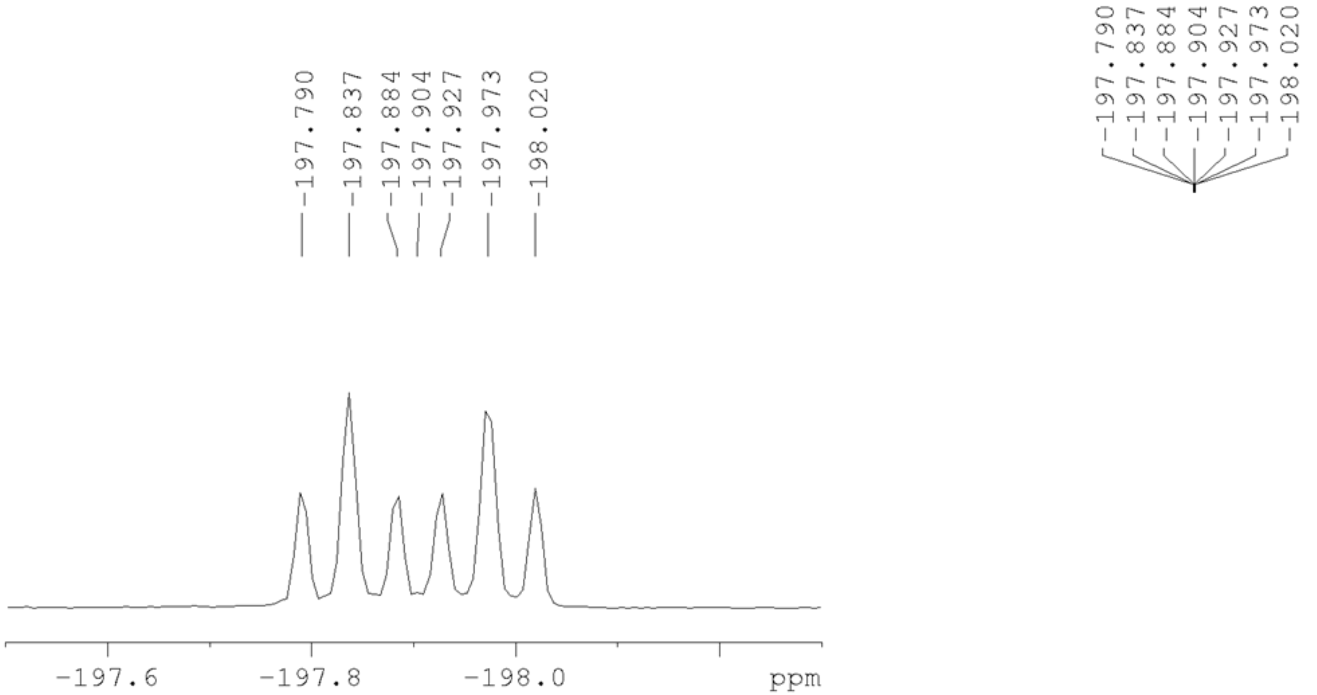

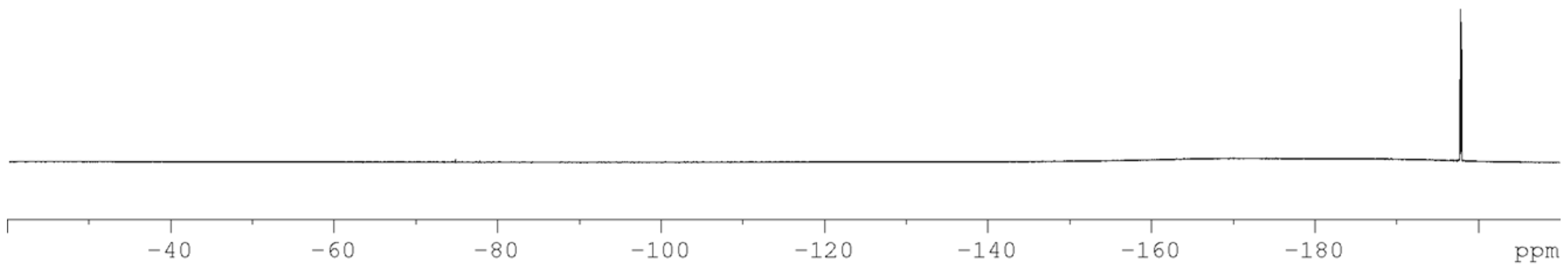

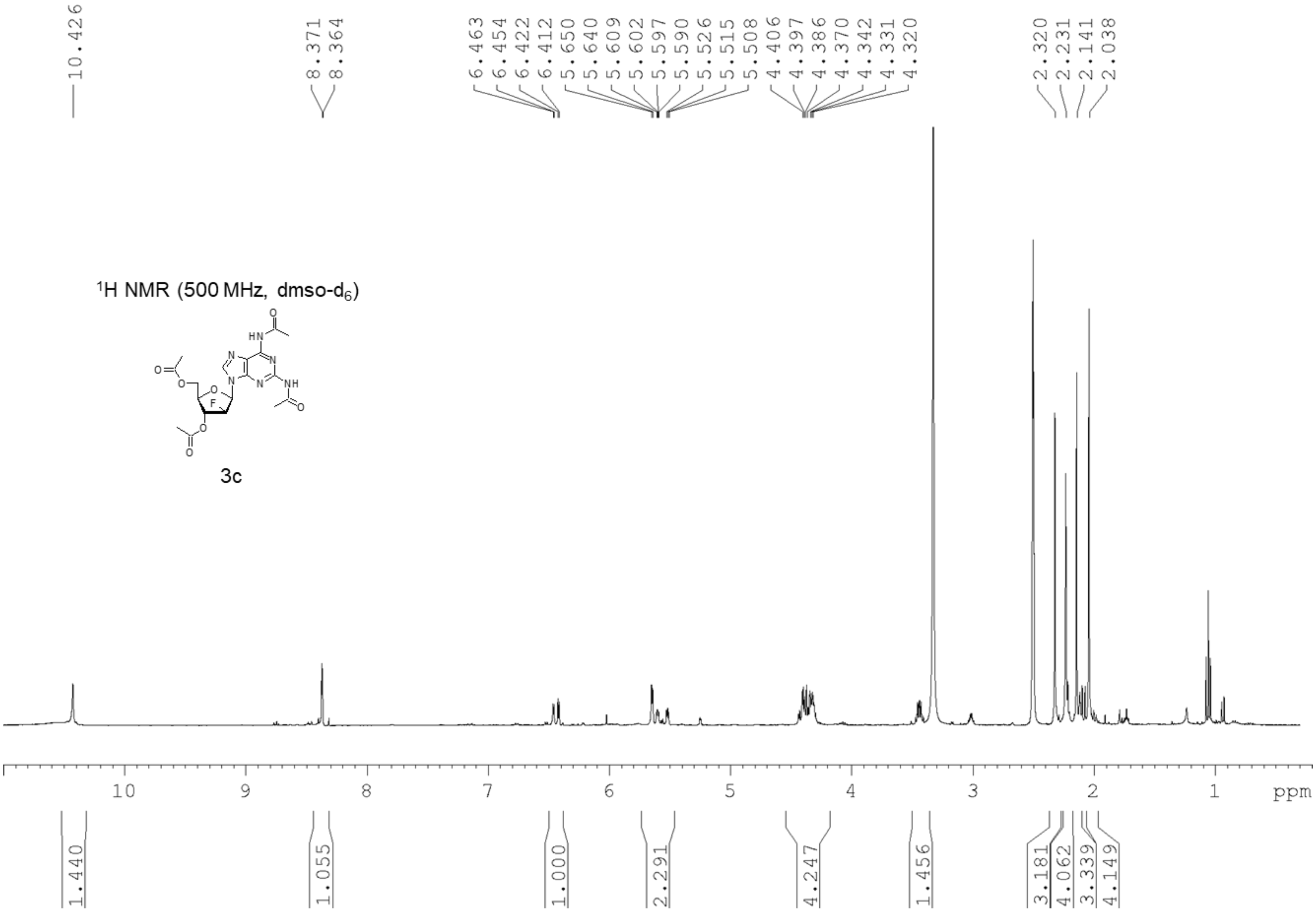

^{1}H NMR (500 MHz, dmso-d$_6$)
3c
10.426
8.371
8.364
6.463
6.454
6.422
6.412
5.650
5.640
5.609
5.602
5.597
5.590
5.526
5.515
5.508
4.406
4.397
4.386
4.370
4.342
4.331
4.320
2.320
2.231
2.141
2.038
10
9
8
7
6
5
4
3
2
1
ppm
1.440
1.055
1.000
2.291
4.247
1.456
3.181
4.062
3.339
4.149

^{13}C NMR (125 MHz, dmso-d_6)

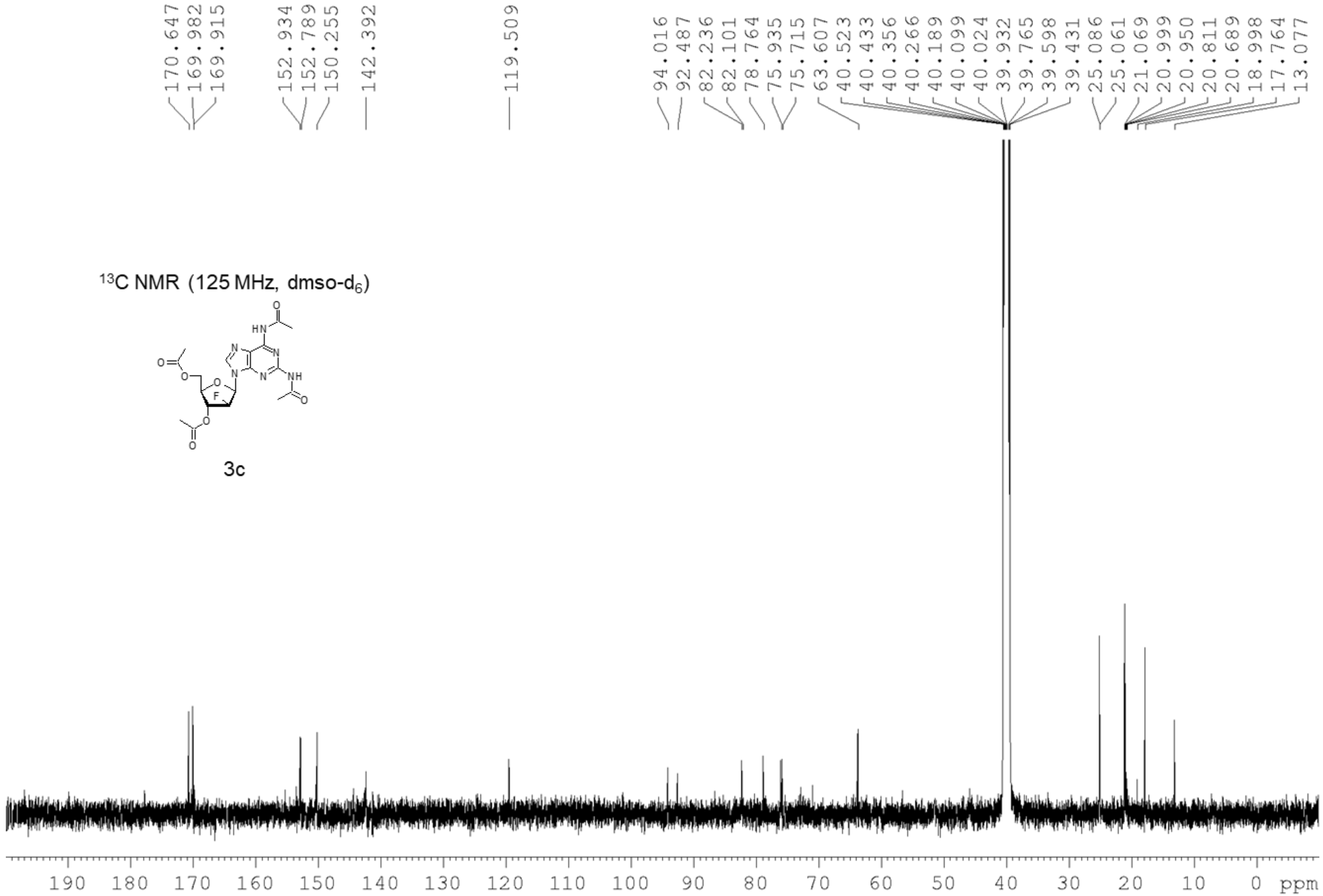

3c

^{19}F NMR (470 MHz, dmso-d_6)

3c

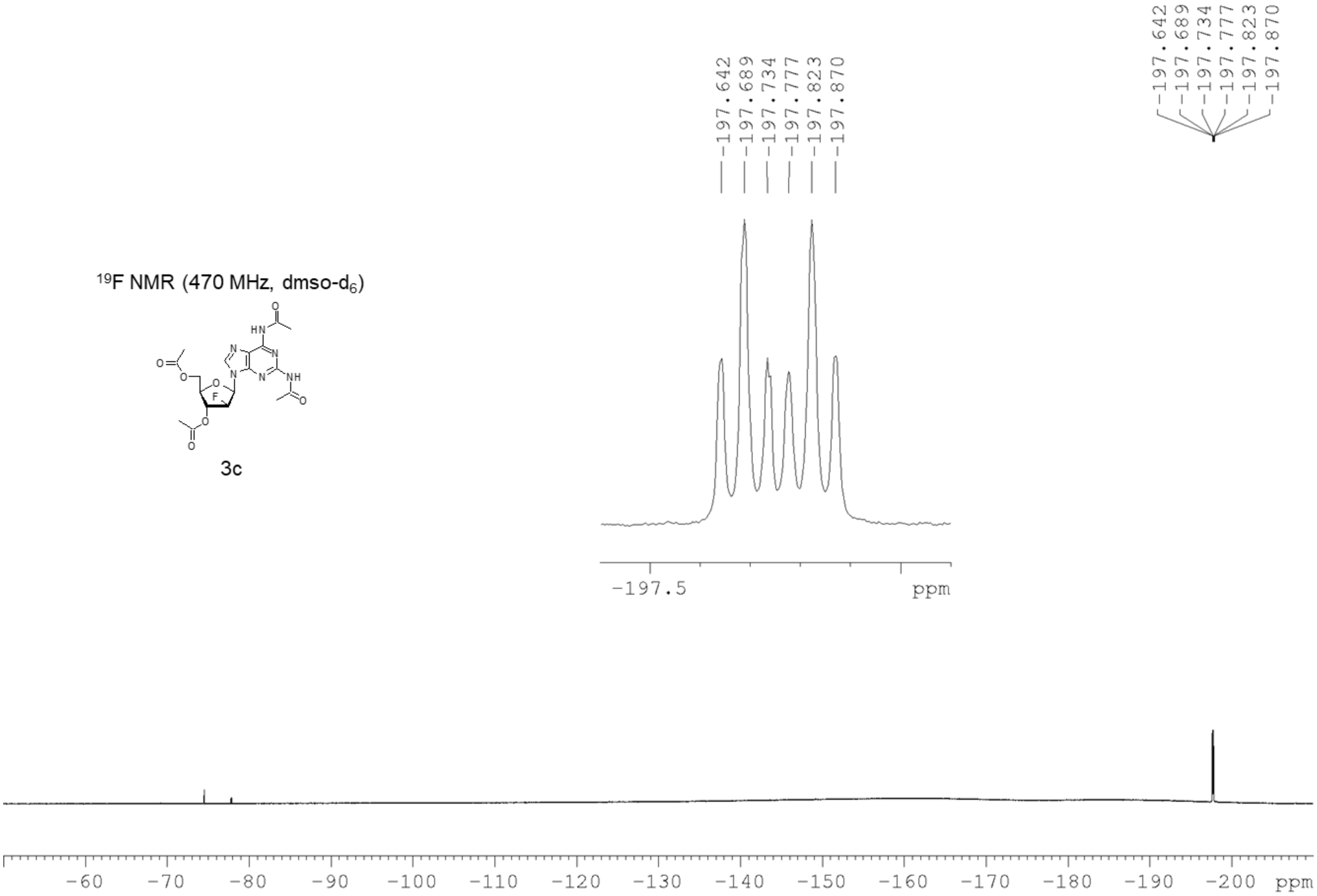

^{1}H NMR (500 MHz, dmso-d_6)

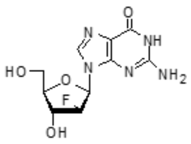

4a

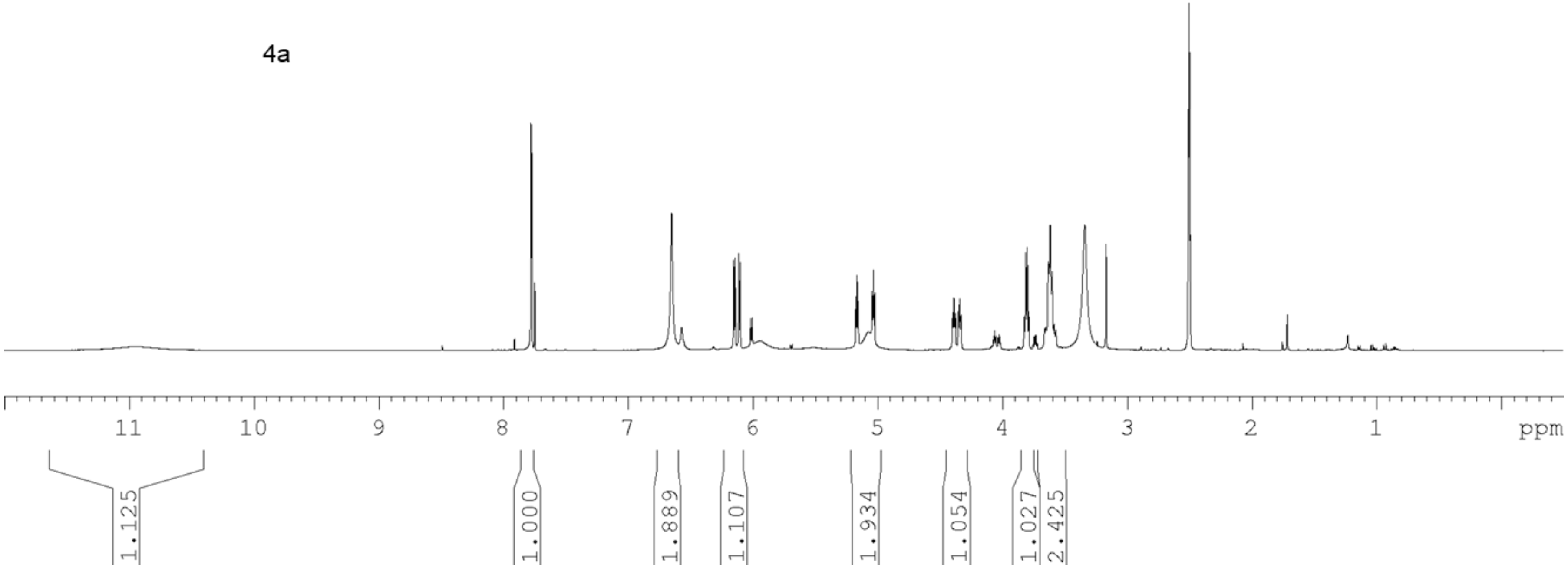

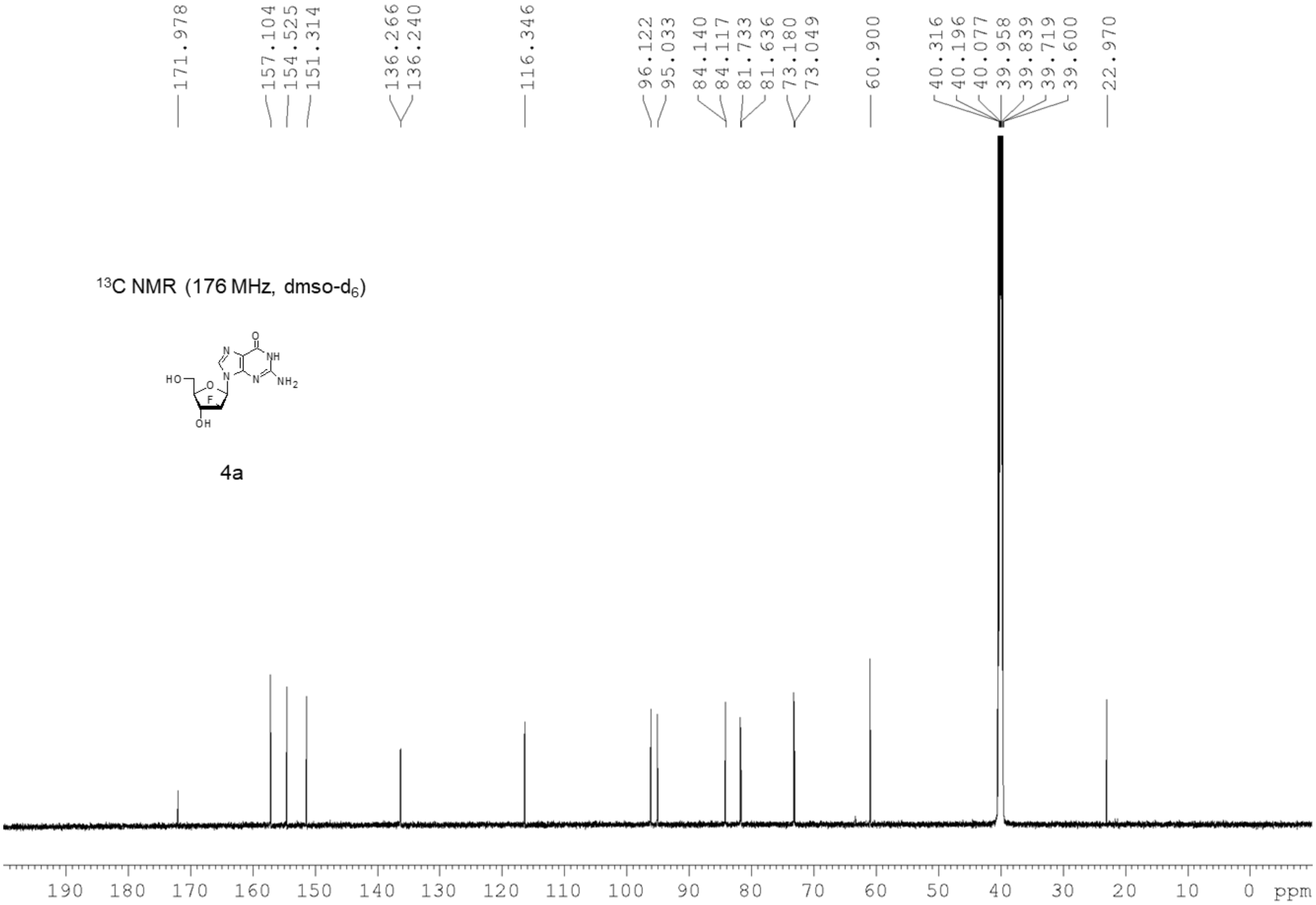

171.978
157.104
154.525
151.314
136.266
136.240
116.346
96.122
95.033
84.140
84.117
81.733
81.636
73.180
73.049
60.900
40.316
40.196
40.077
39.958
39.839
39.719
39.600
22.970
^{13}C NMR (176 MHz, dmso-d_6)
HO
O
F
OH
N
NH
NH$_2$
O
4a
190 180 170 160 150 140 130 120 110 100 90 80 70 60 50 40 30 20 10 0 ppm

^{19}F NMR (376 MHz, dmso-d_6)

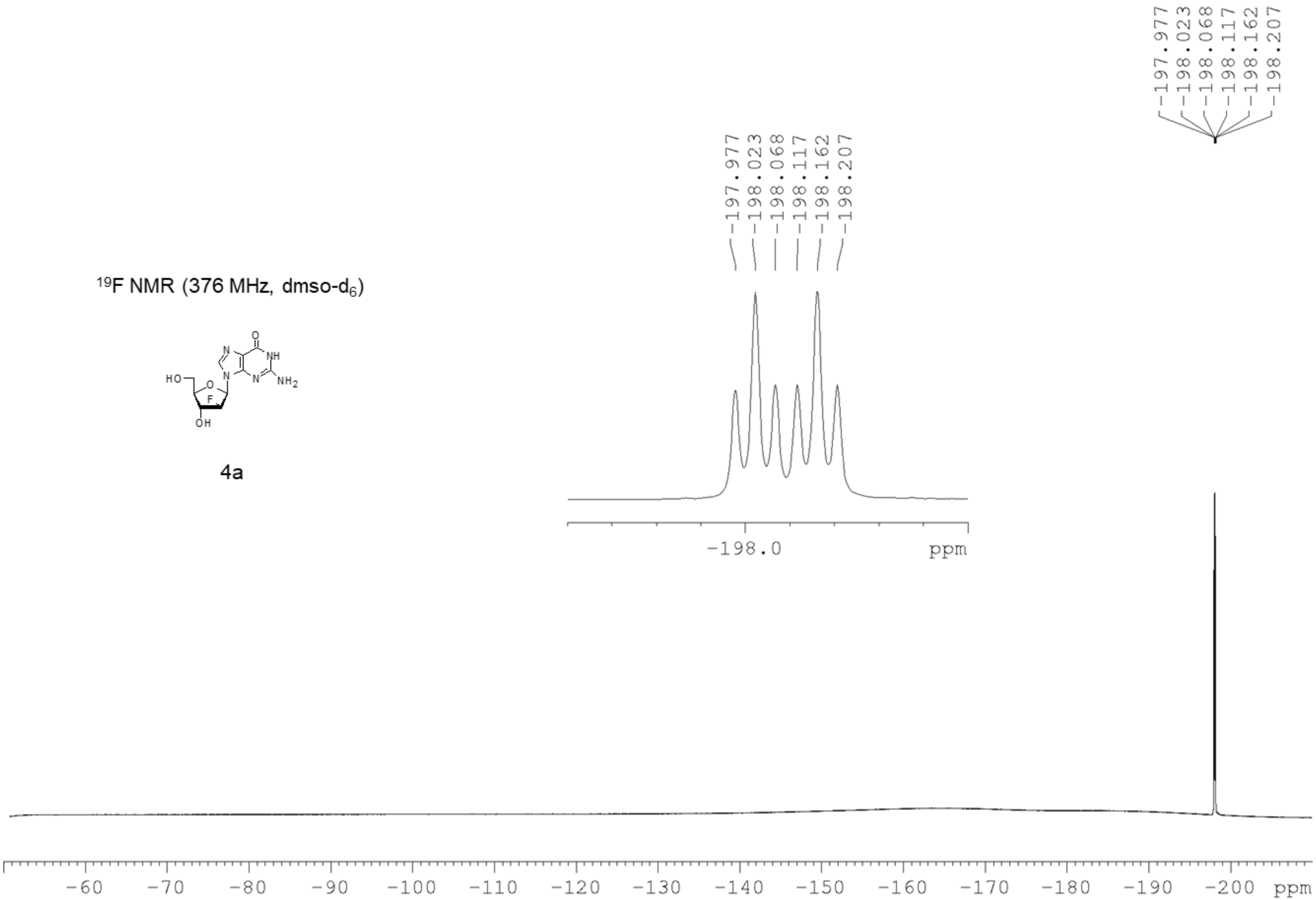

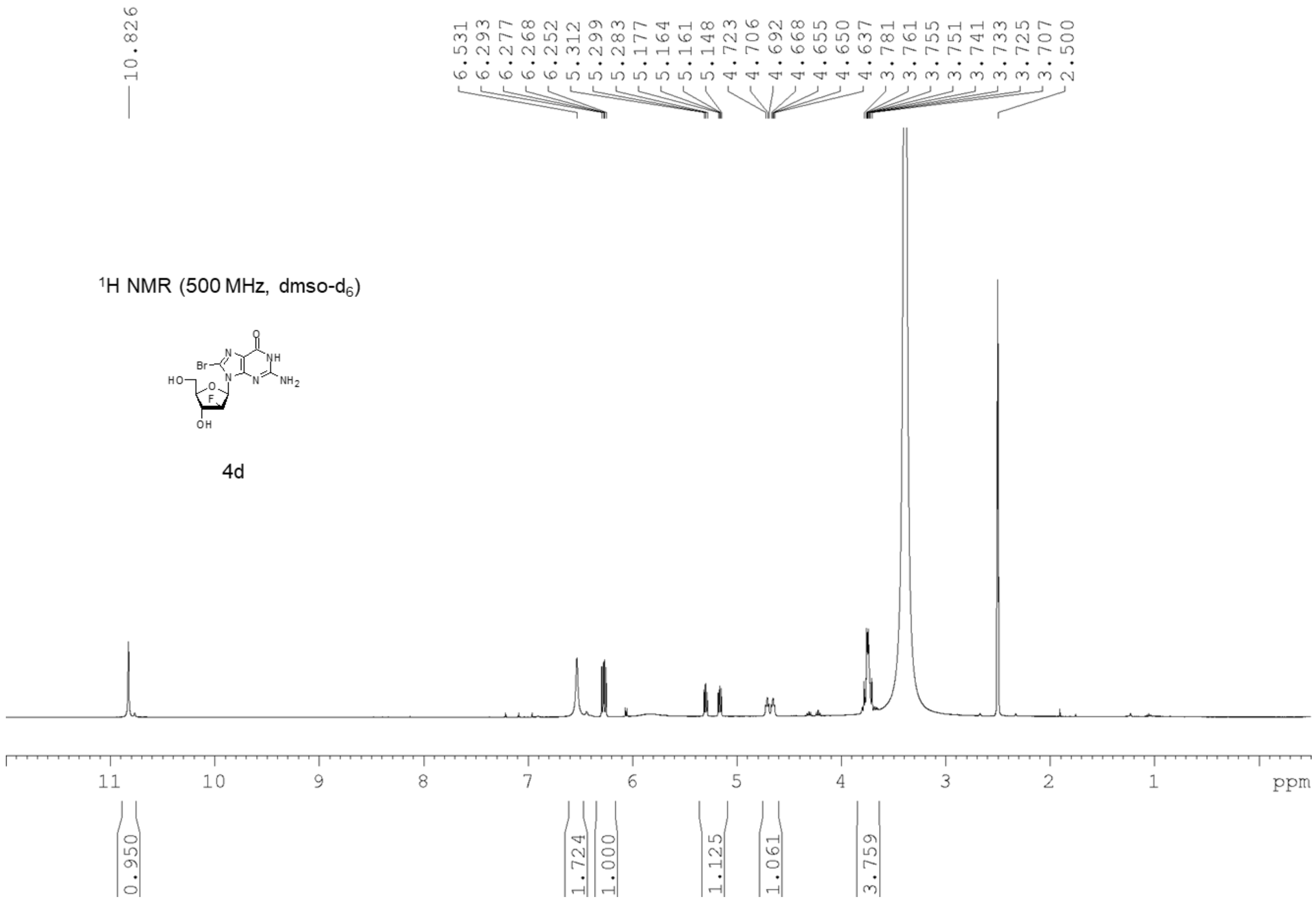
^{1}H NMR (500 MHz, dmso-d$_6$)
10.826
6.531
6.293
6.277
6.268
6.252
5.312
5.299
5.283
5.177
5.164
5.161
5.148
4.723
4.706
4.692
4.668
4.655
4.650
4.637
3.781
3.761
3.755
3.751
3.741
3.733
3.725
3.707
2.500
Br
HO
O
F
OH
N
NH
NH$_2$
O
11
10
9
8
7
6
5
4
3
2
1
ppm
0.950
1.724
1.000
1.125
1.061
3.759

4d

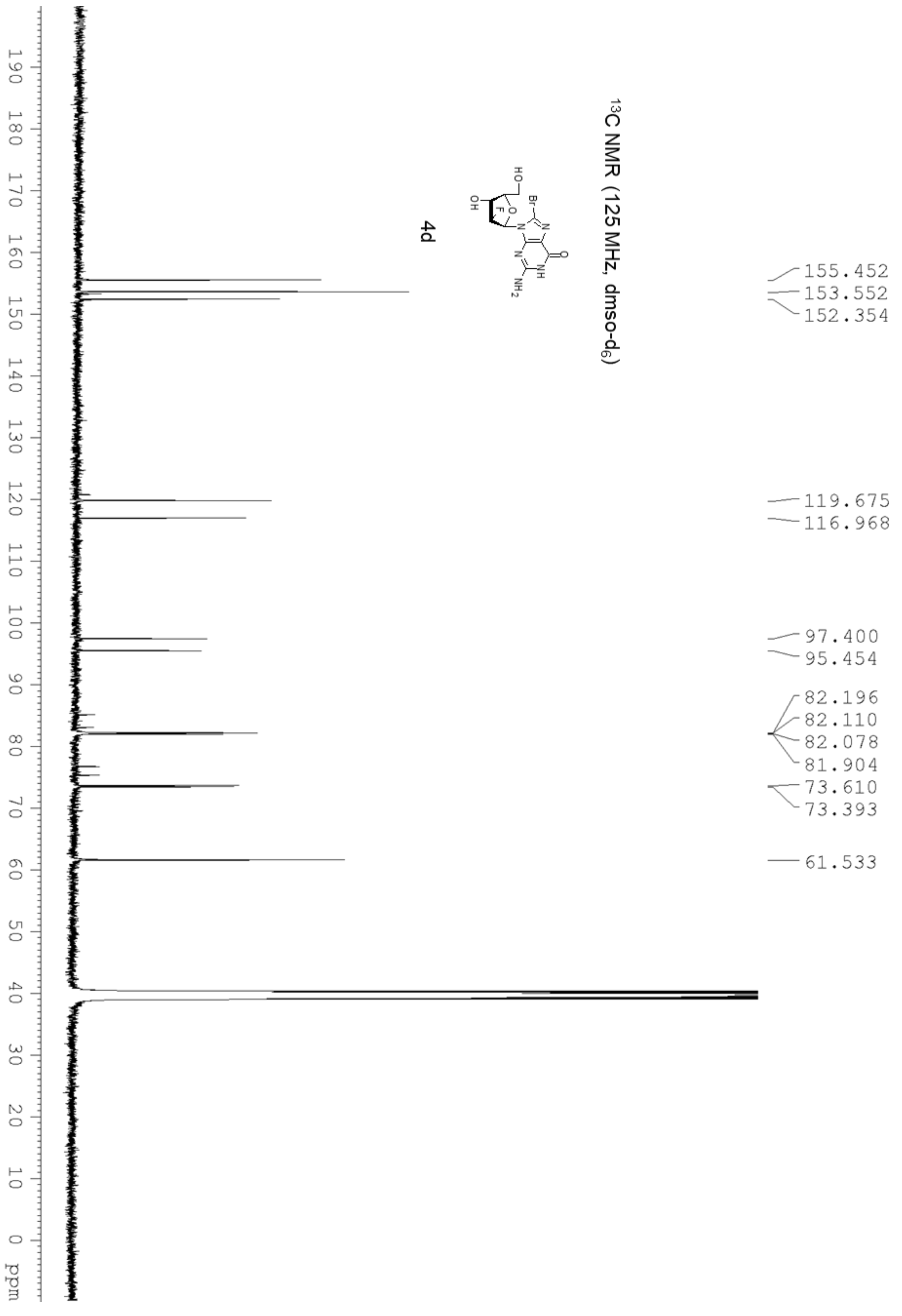

^{13}C NMR (125 MHz, dmso-d$_6$)
4d
155.452
153.552
152.354
119.675
116.968
97.400
95.454
82.196
82.110
82.078
81.904
73.610
73.393
61.533
190 180 170 160 150 140 130 120 110 100 90 80 70 60 50 40 30 20 10 0 ppm

^{19}F NMR (376 MHz, dmso-d_6)

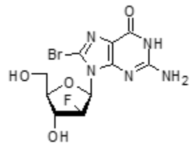

4d

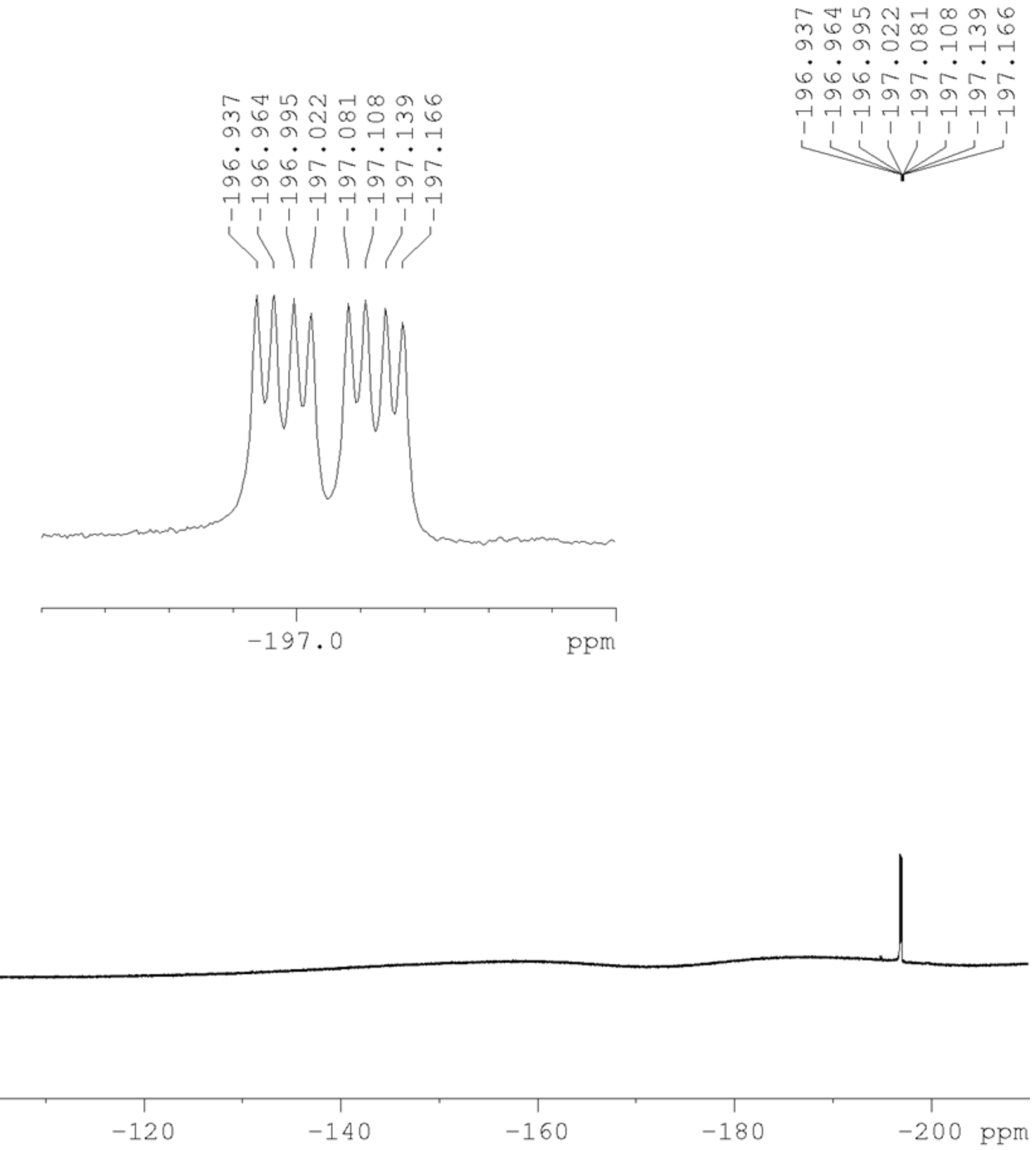

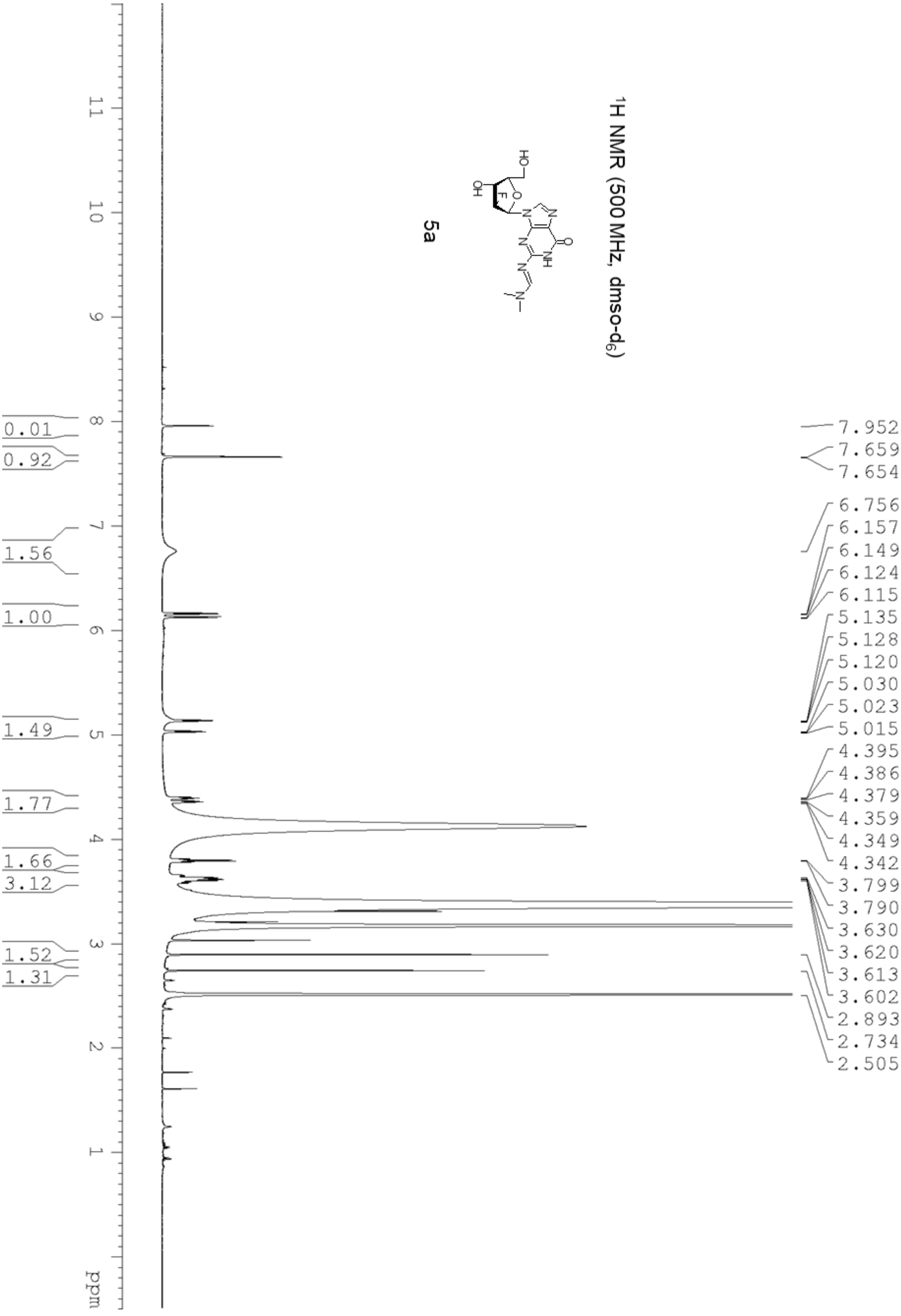

1H NMR (500 MHz, dmso-d6)
5a
7.952
7.659
7.654
6.756
6.157
6.149
6.124
6.115
5.135
5.128
5.120
5.030
5.023
5.015
4.395
4.386
4.379
4.359
4.349
4.342
3.799
3.790
3.630
3.620
3.613
3.602
2.893
2.734
2.505
0.01
0.92
1.56
1.00
1.49
1.77
1.66
3.12
1.52
1.31
ppm

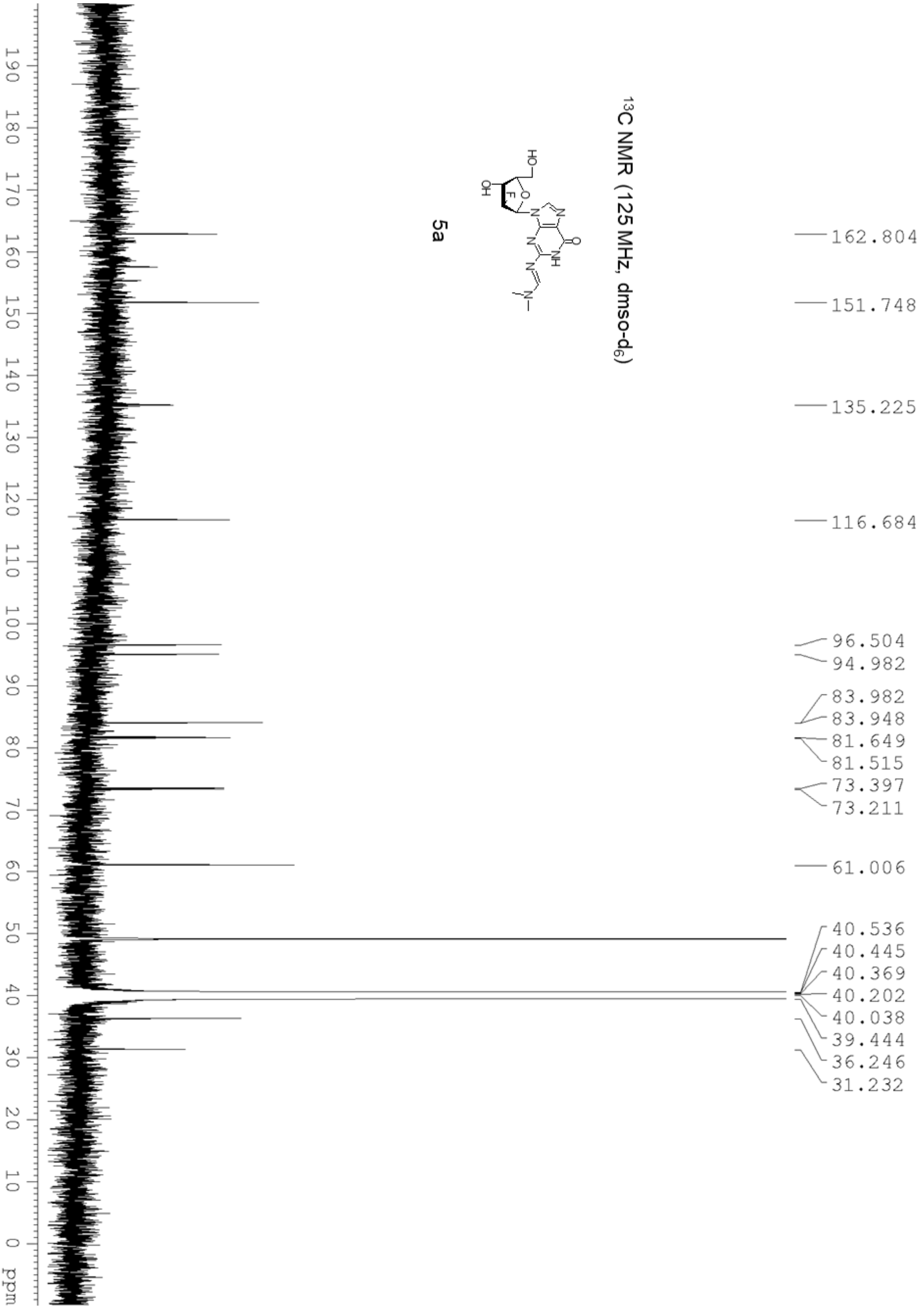

13C NMR (125 MHz, dmso-d6)
5a
162.804
151.748
135.225
116.684
96.504
94.982
83.982
83.948
81.649
81.515
73.397
73.211
61.006
40.536
40.445
40.369
40.202
40.038
39.444
36.246
31.232
ppm

^{19}F NMR (376 MHz, dmso-d_6)

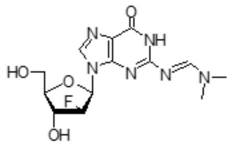

5a

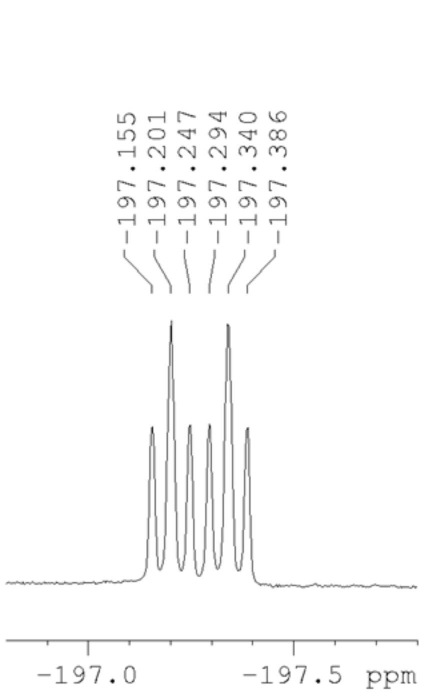

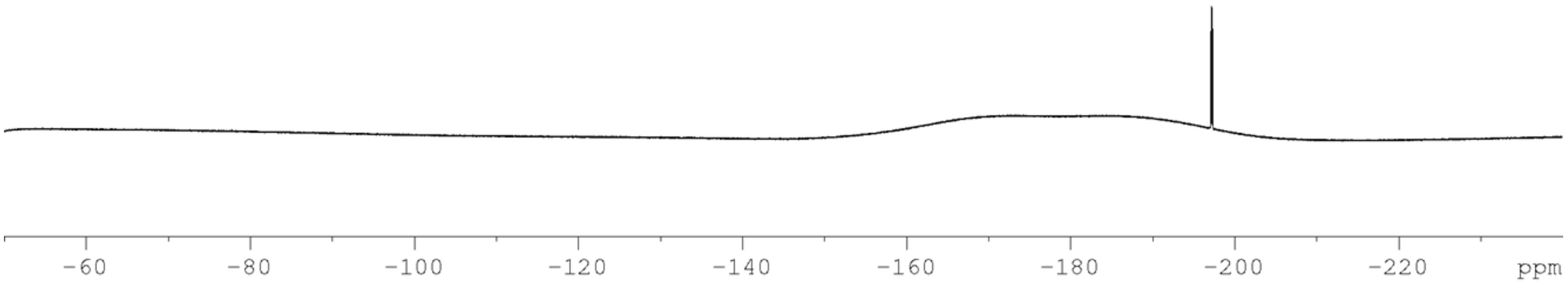

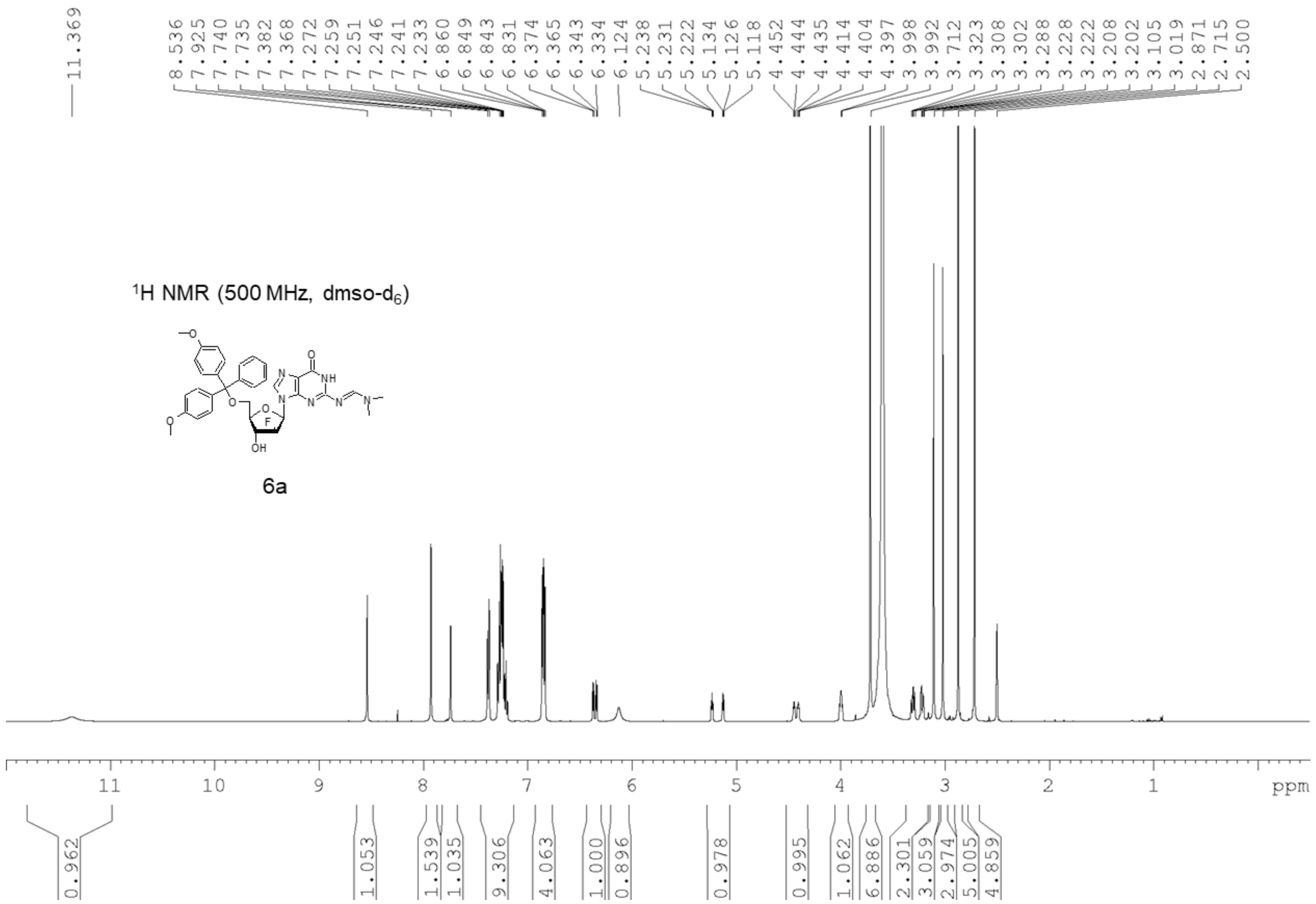

^{1}H NMR (500 MHz, dmso-d_6)
6a
11.369
8.536 7.925 7.740 7.735 7.382 7.368 7.272 7.259 7.251 7.246 7.241 7.233 6.860 6.849 6.843 6.831 6.374 6.365 6.343 6.334 6.124 5.238 5.231 5.222 5.134 5.126 5.118 4.452 4.444 4.435 4.414 4.404 4.397 3.998 3.992 3.712 3.323 3.308 3.302 3.288 3.228 3.222 3.208 3.202 3.105 3.019 2.871 2.715 2.500
11 10 9 8 7 6 5 4 3 2 1 ppm
0.962 1.053 1.539 1.035 9.306 4.063 1.000 0.896 0.978 0.995 1.062 6.886 2.301 3.059 2.974 5.005 4.859

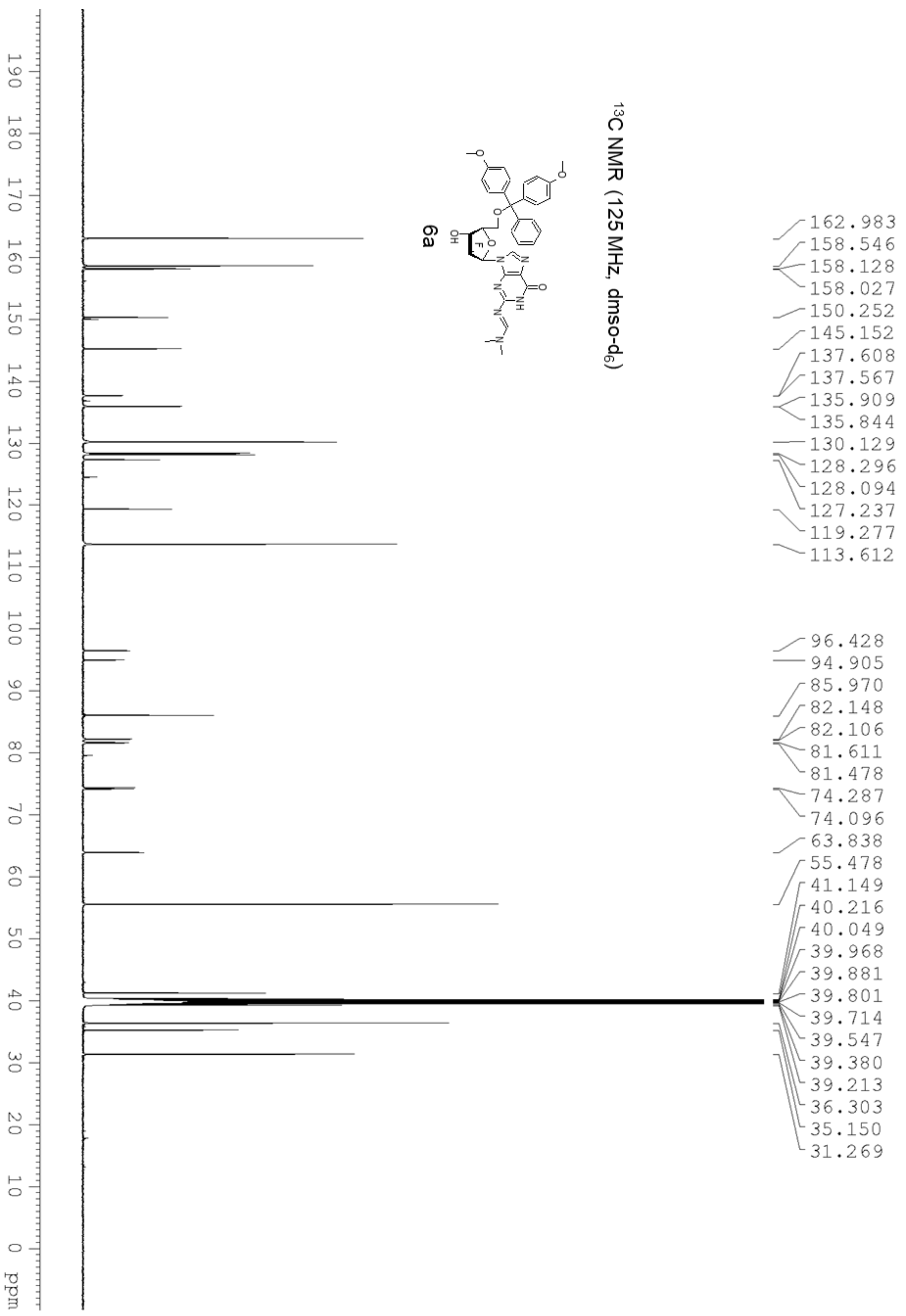

13C NMR (125 MHz, dmso-d6)
6a
162.983
158.546
158.128
158.027
150.252
145.152
137.608
137.567
135.909
135.844
130.129
128.296
128.094
127.237
119.277
113.612
96.428
94.905
85.970
82.148
82.106
81.611
81.478
74.287
74.096
63.838
55.478
41.149
40.216
40.049
39.968
39.881
39.801
39.714
39.547
39.380
39.213
36.303
35.150
31.269
190 180 170 160 150 140 130 120 110 100 90 80 70 60 50 40 30 20 10 0 ppm

^{19}F NMR (470 MHz, dmso-d_6)

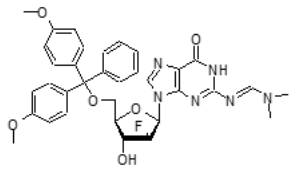

6a

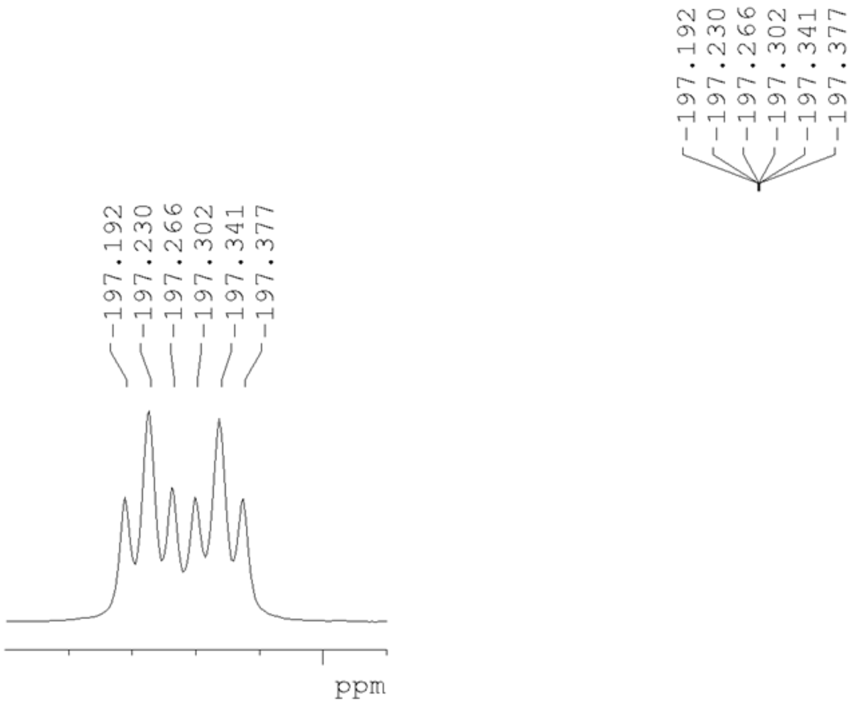

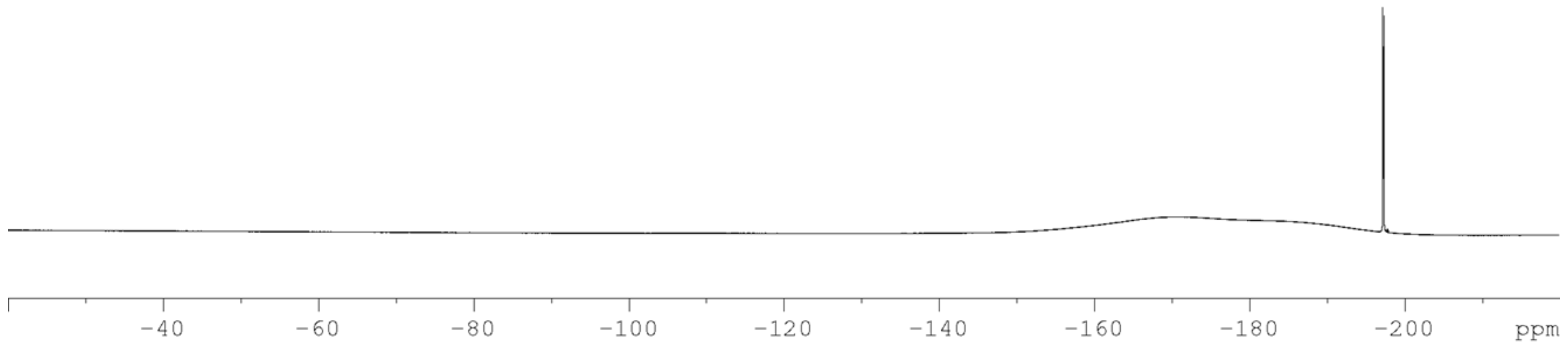

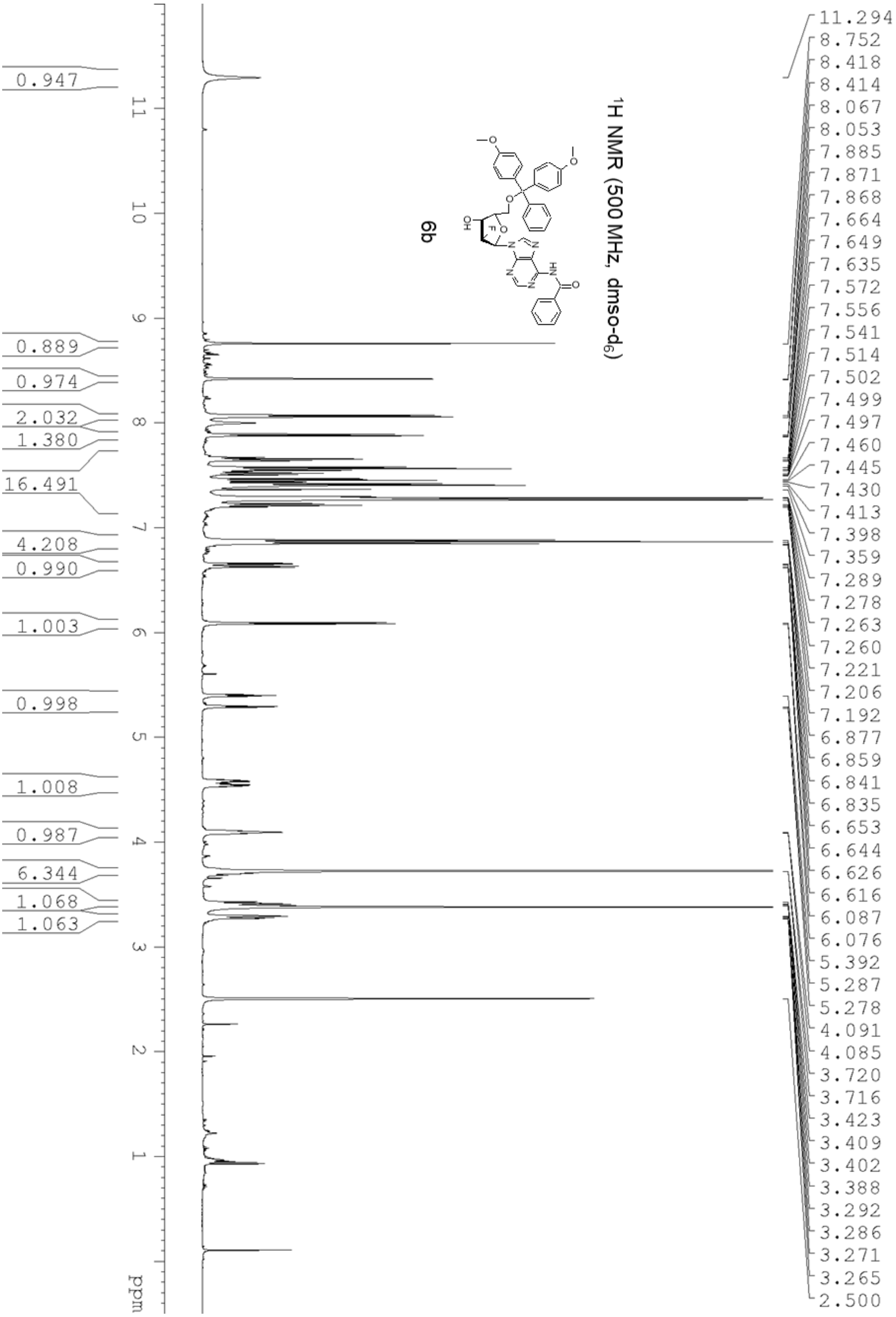

^{1}H NMR (500 MHz, dmso-d$_6$)
6b
11.294
8.752
8.418
8.414
8.067
8.053
7.885
7.871
7.868
7.664
7.649
7.635
7.572
7.556
7.541
7.514
7.502
7.499
7.497
7.460
7.445
7.430
7.413
7.398
7.359
7.289
7.278
7.263
7.260
7.221
7.206
7.192
6.877
6.859
6.841
6.835
6.653
6.644
6.626
6.616
6.087
6.076
5.392
5.287
5.278
4.091
4.085
3.720
3.716
3.423
3.409
3.402
3.388
3.292
3.286
3.271
3.265
2.500
0.947
0.889
0.974
2.032
1.380
16.491
4.208
0.990
1.003
0.998
1.008
0.987
6.344
1.068
1.063
ppm

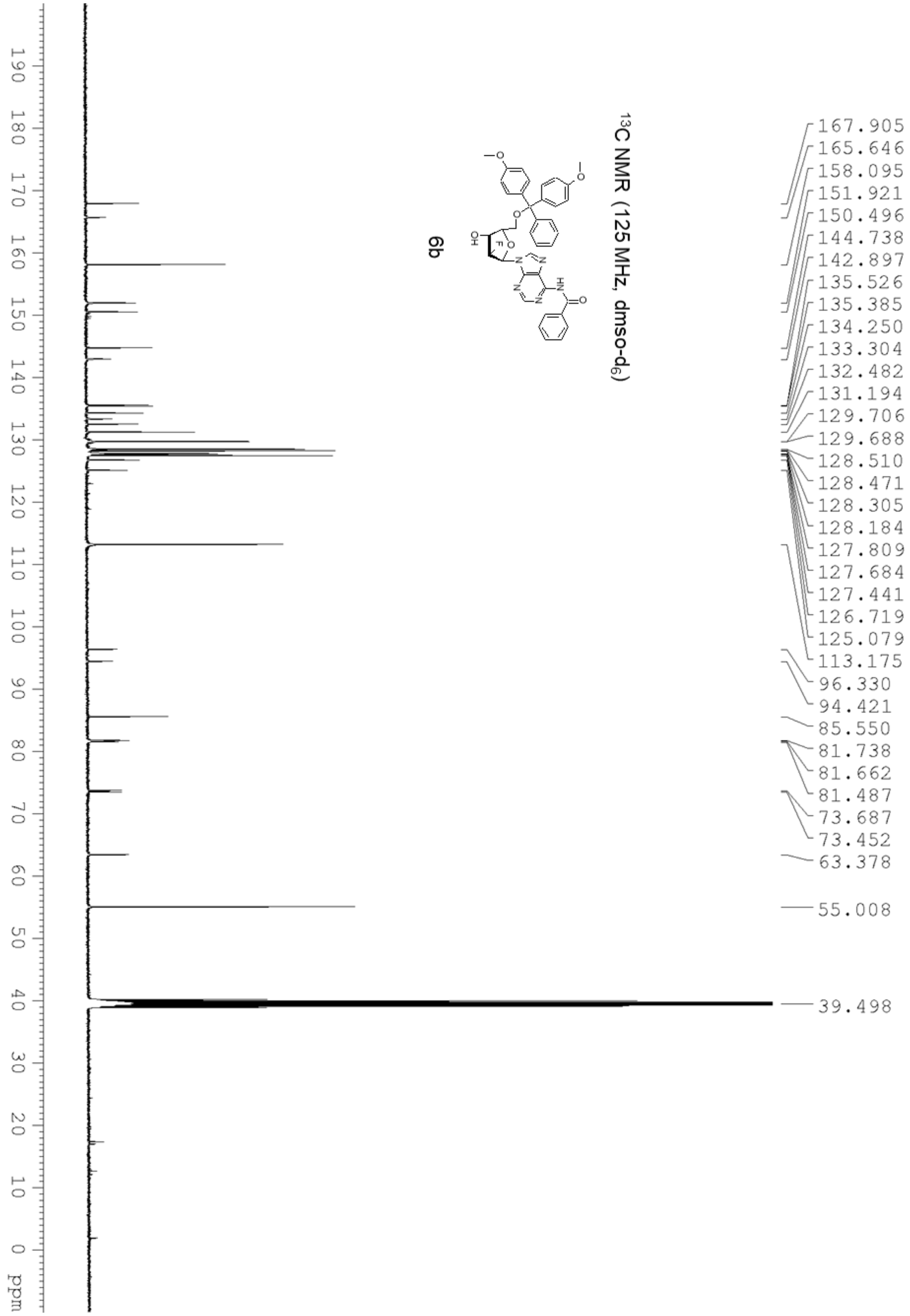

^{13}C NMR (125 MHz, dmso-d$_6$)
6b
167.905
165.646
158.095
151.921
150.496
144.738
142.897
135.526
135.385
134.250
133.304
132.482
131.194
129.706
129.688
128.510
128.471
128.305
128.184
127.809
127.684
127.441
126.719
125.079
113.175
96.330
94.421
85.550
81.738
81.662
81.487
73.687
73.452
63.378
55.008
39.498
190 180 170 160 150 140 130 120 110 100 90 80 70 60 50 40 30 20 10 0 ppm

^{19}F NMR (470 MHz, dmso-d_6)

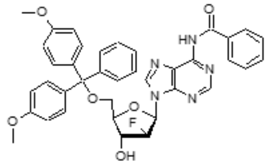

6b

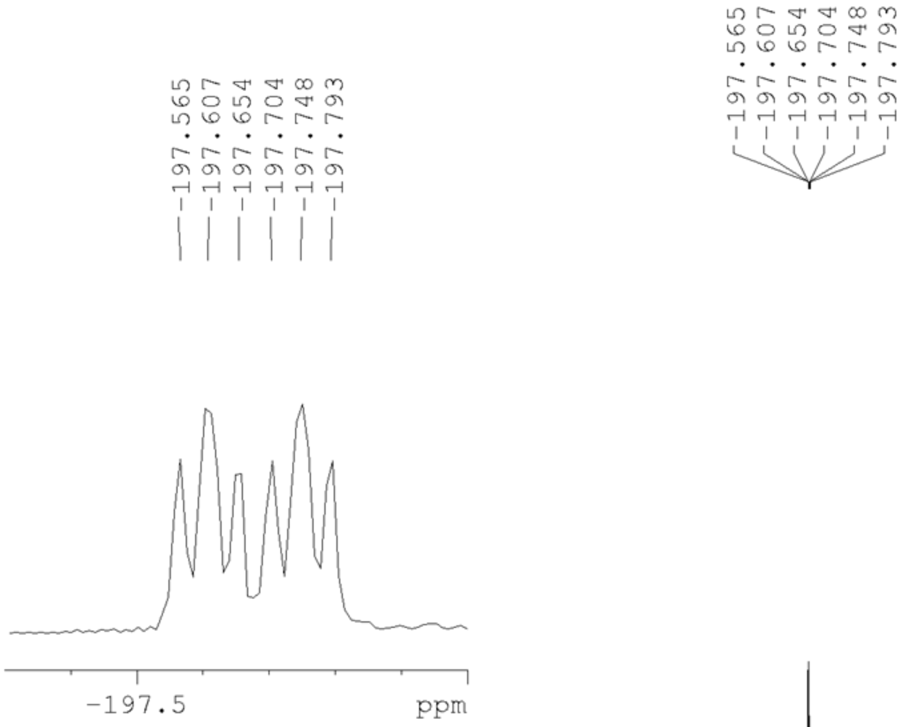

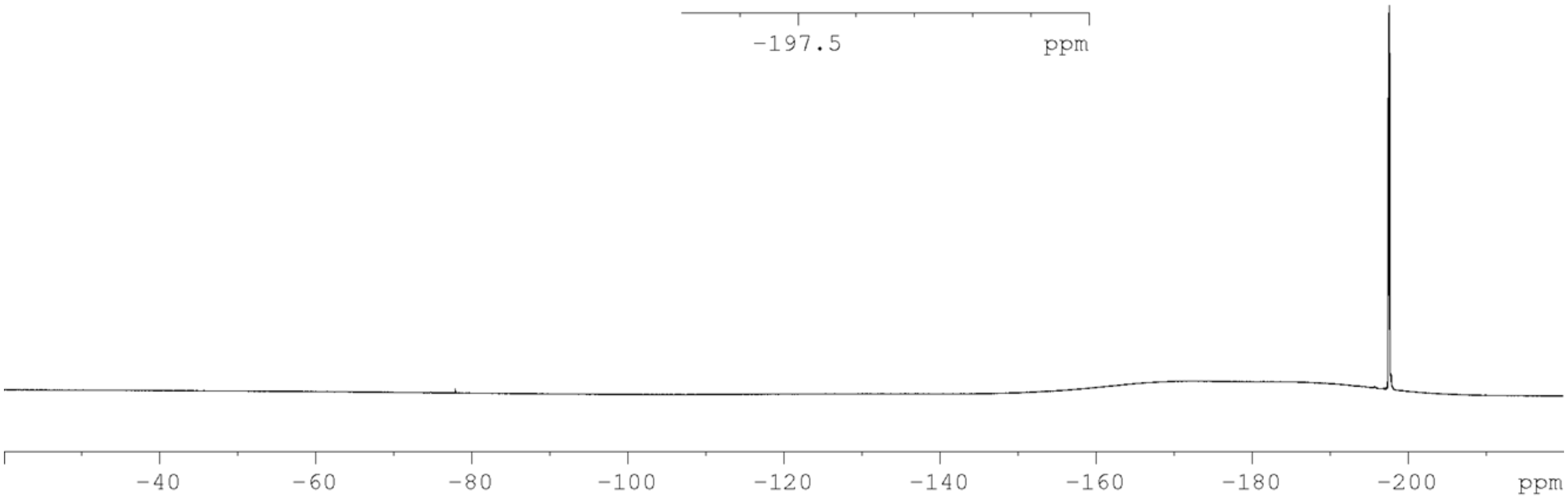

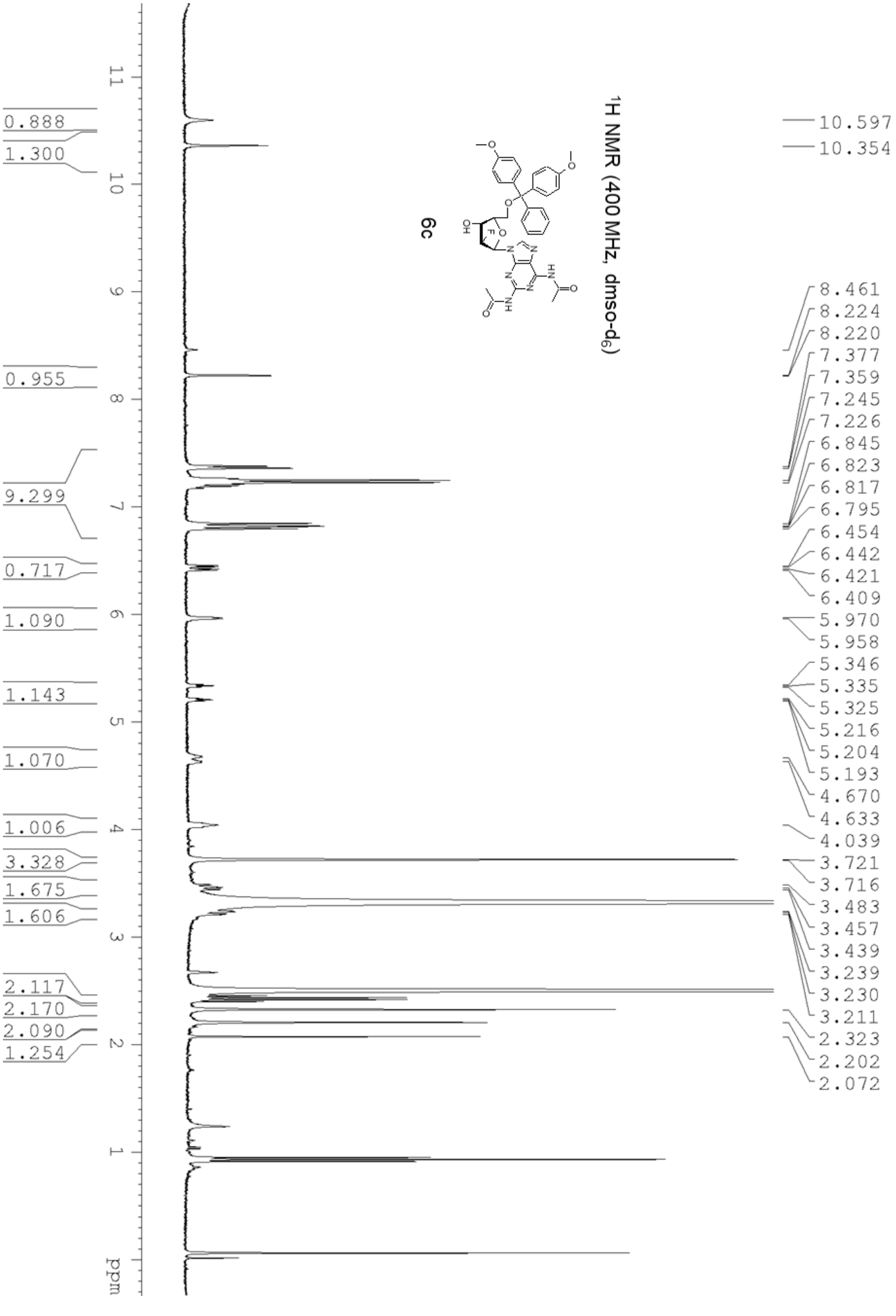

1H NMR (400 MHz, dmso-d6)
6c
10.597
10.354
8.461
8.224
8.220
7.377
7.359
7.245
7.226
6.845
6.823
6.817
6.795
6.454
6.442
6.421
6.409
5.970
5.958
5.346
5.335
5.325
5.216
5.204
5.193
4.670
4.633
4.039
3.721
3.716
3.483
3.457
3.439
3.239
3.230
3.211
2.323
2.202
2.072
0.888
1.300
0.955
9.299
0.717
1.090
1.143
1.070
1.006
3.328
1.675
1.606
2.117
2.170
2.090
1.254
ppm

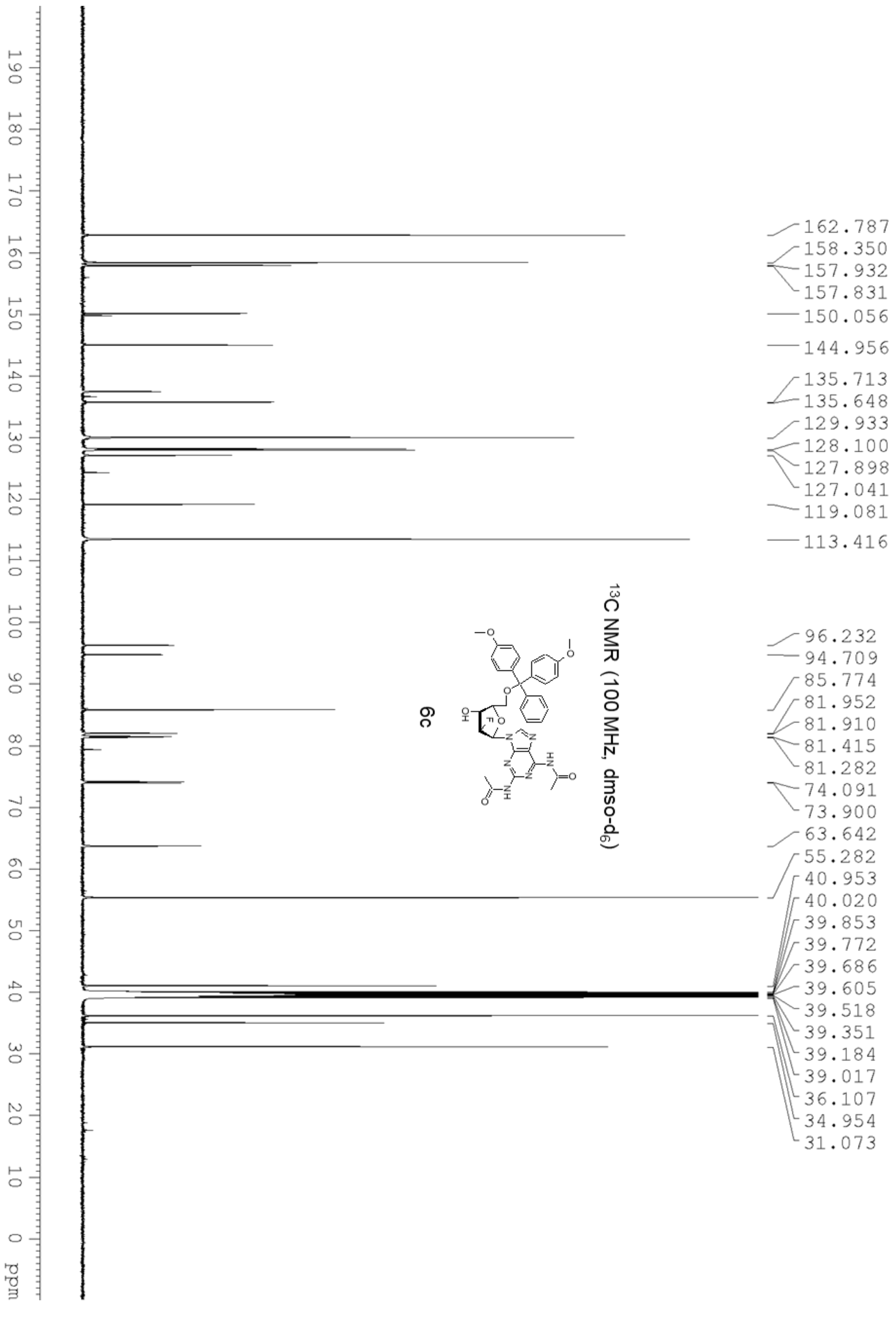

13C NMR (100 MHz, dmso-d6)
6c
162.787
158.350
157.932
157.831
150.056
144.956
135.713
135.648
129.933
128.100
127.898
127.041
119.081
113.416
96.232
94.709
85.774
81.952
81.910
81.415
81.282
74.091
73.900
63.642
55.282
40.953
40.020
39.853
39.772
39.686
39.605
39.518
39.351
39.184
39.017
36.107
34.954
31.073
ppm

^{19}F NMR (376 MHz, dmso-d_6)

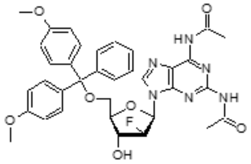

6c

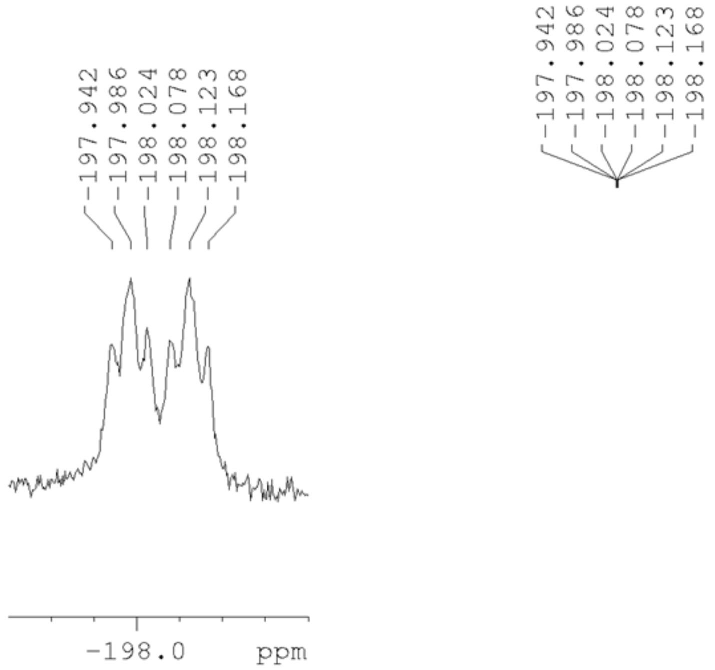

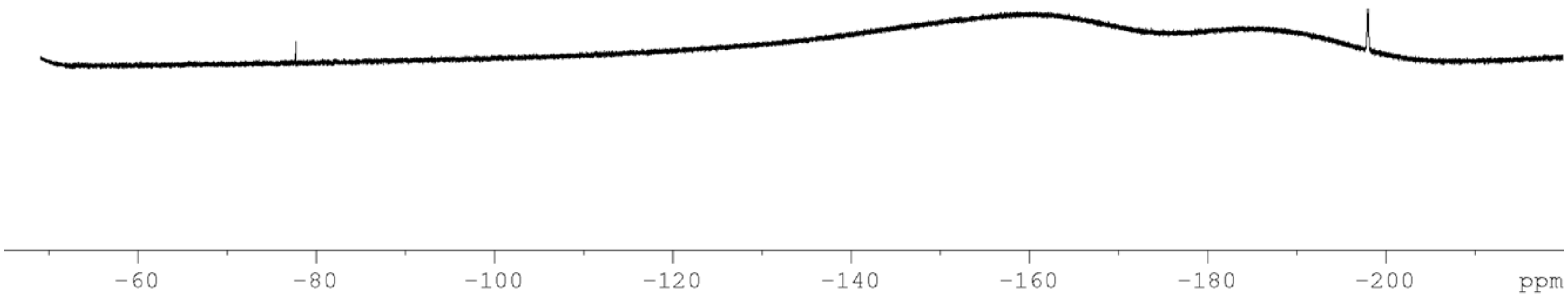

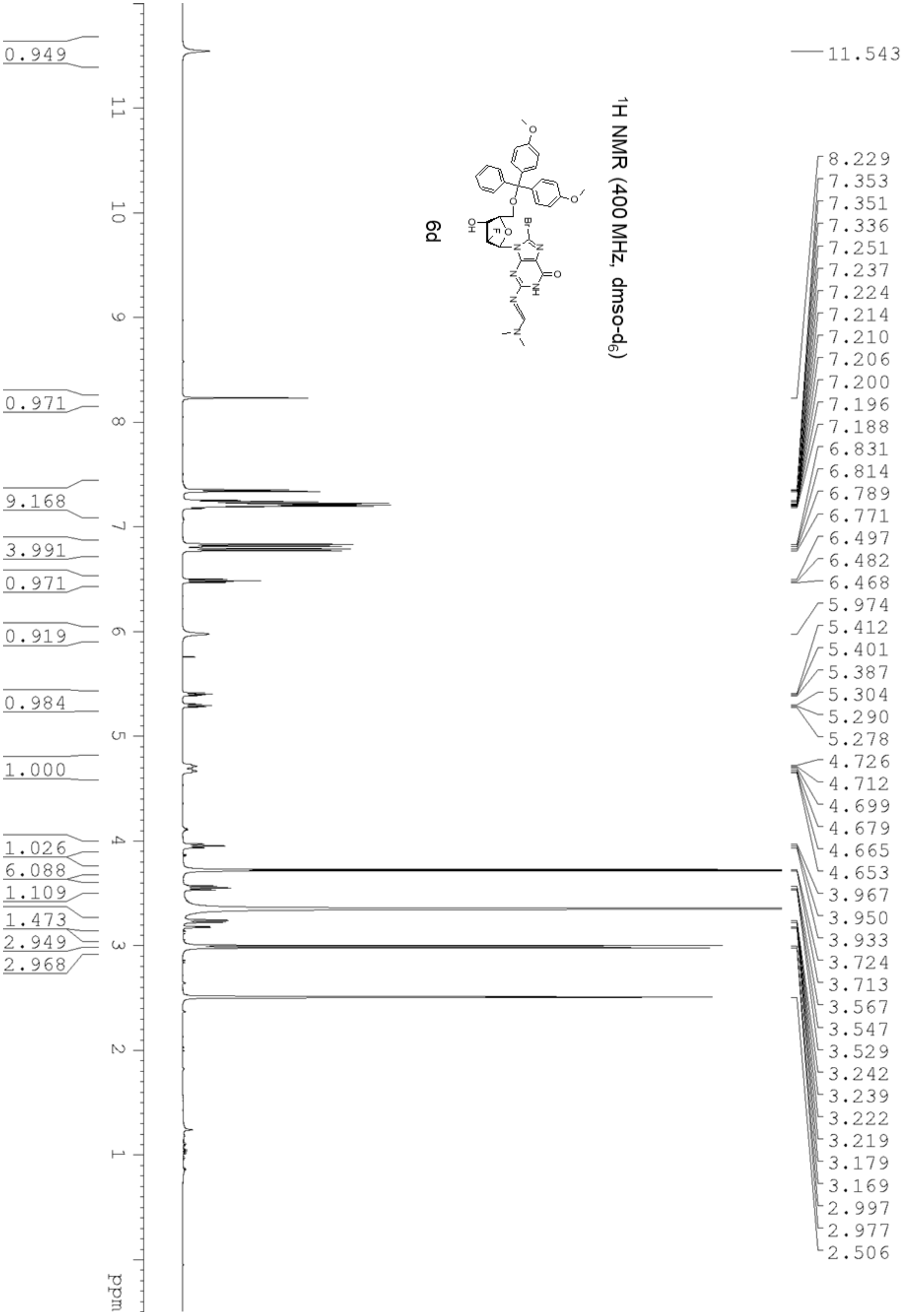
1H NMR (400 MHz, dmso-d6)
6d

^{13}C NMR (100 MHz, dmso-d_6)

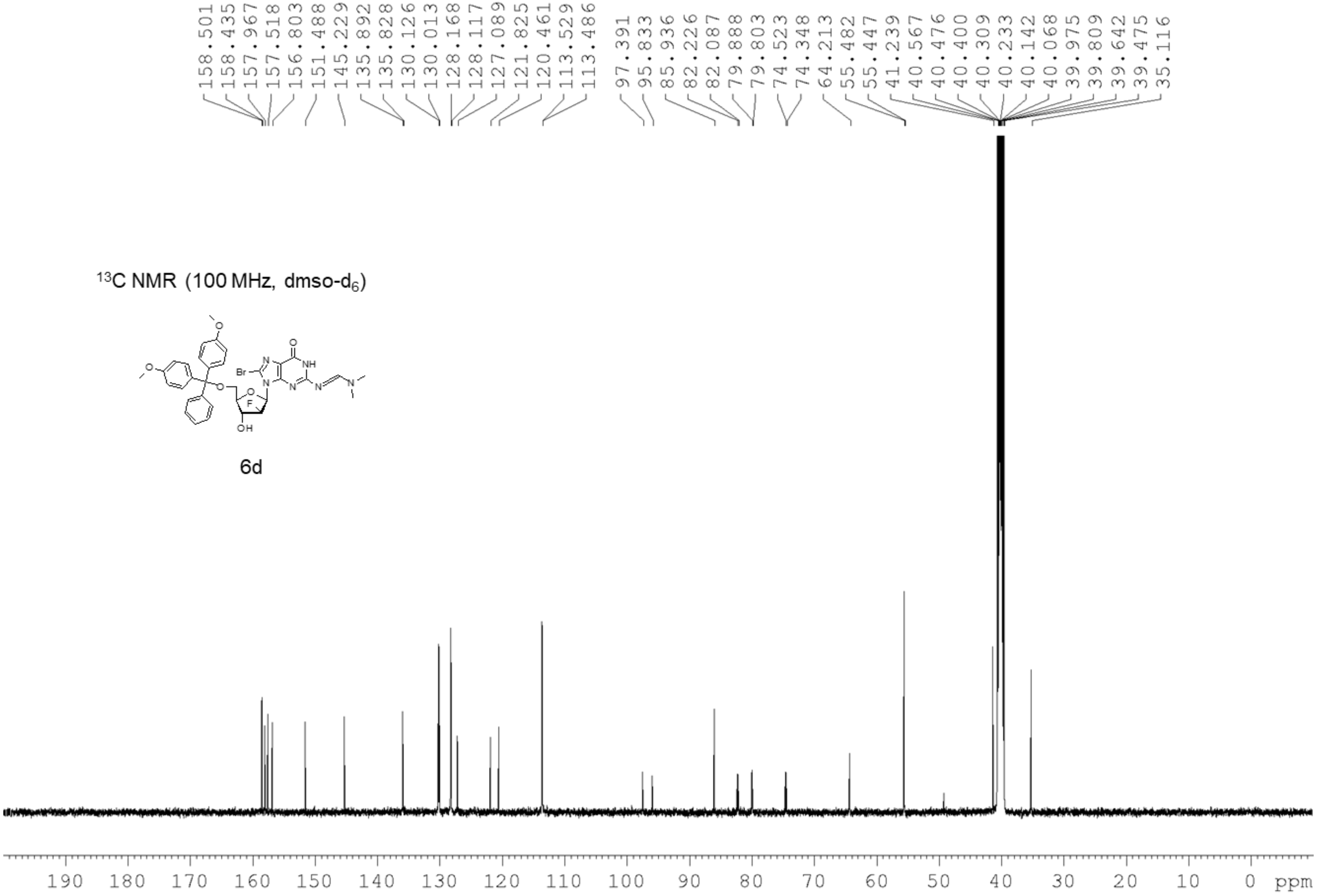

^{19}F NMR (376 MHz, dmso-d_6)

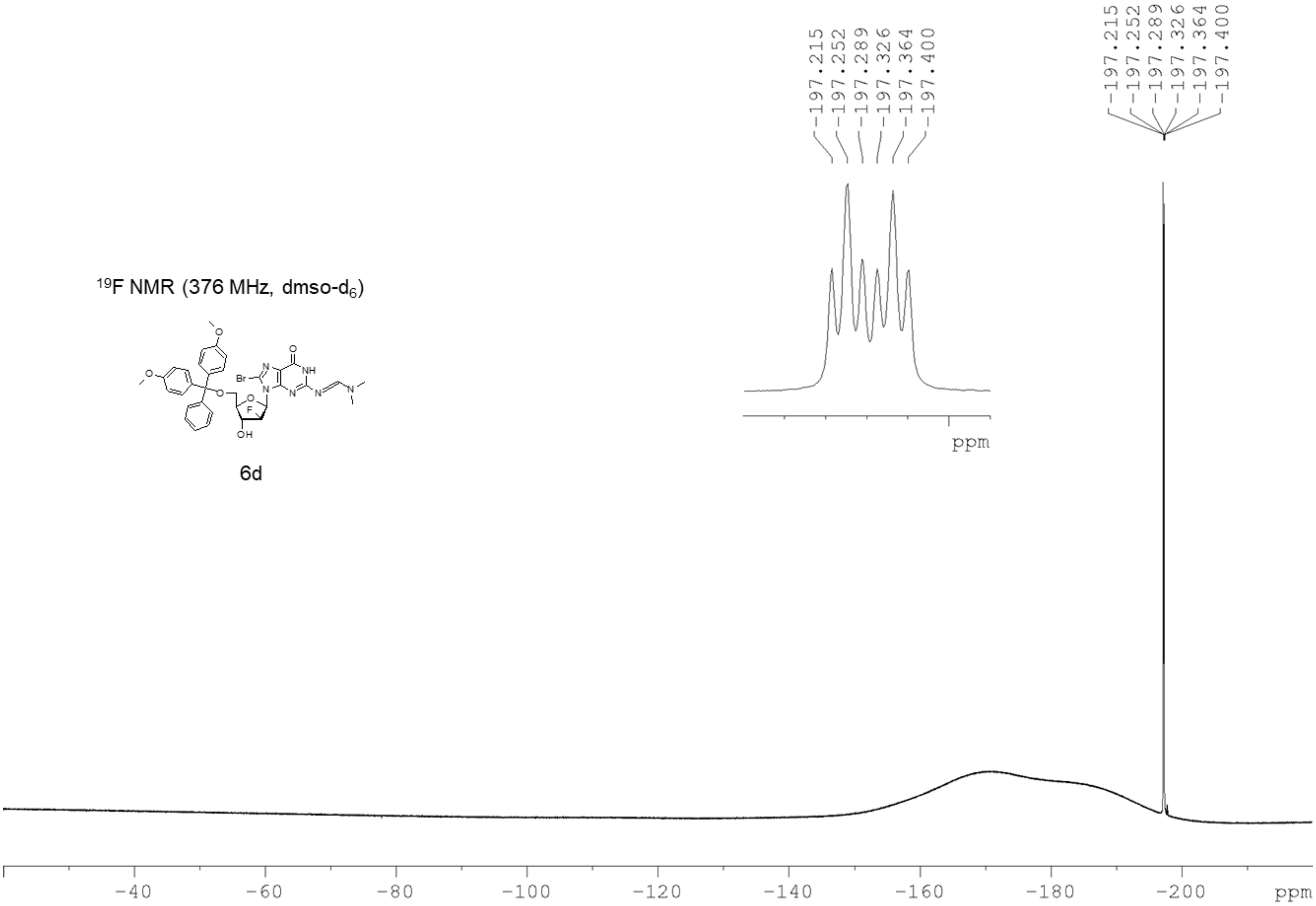

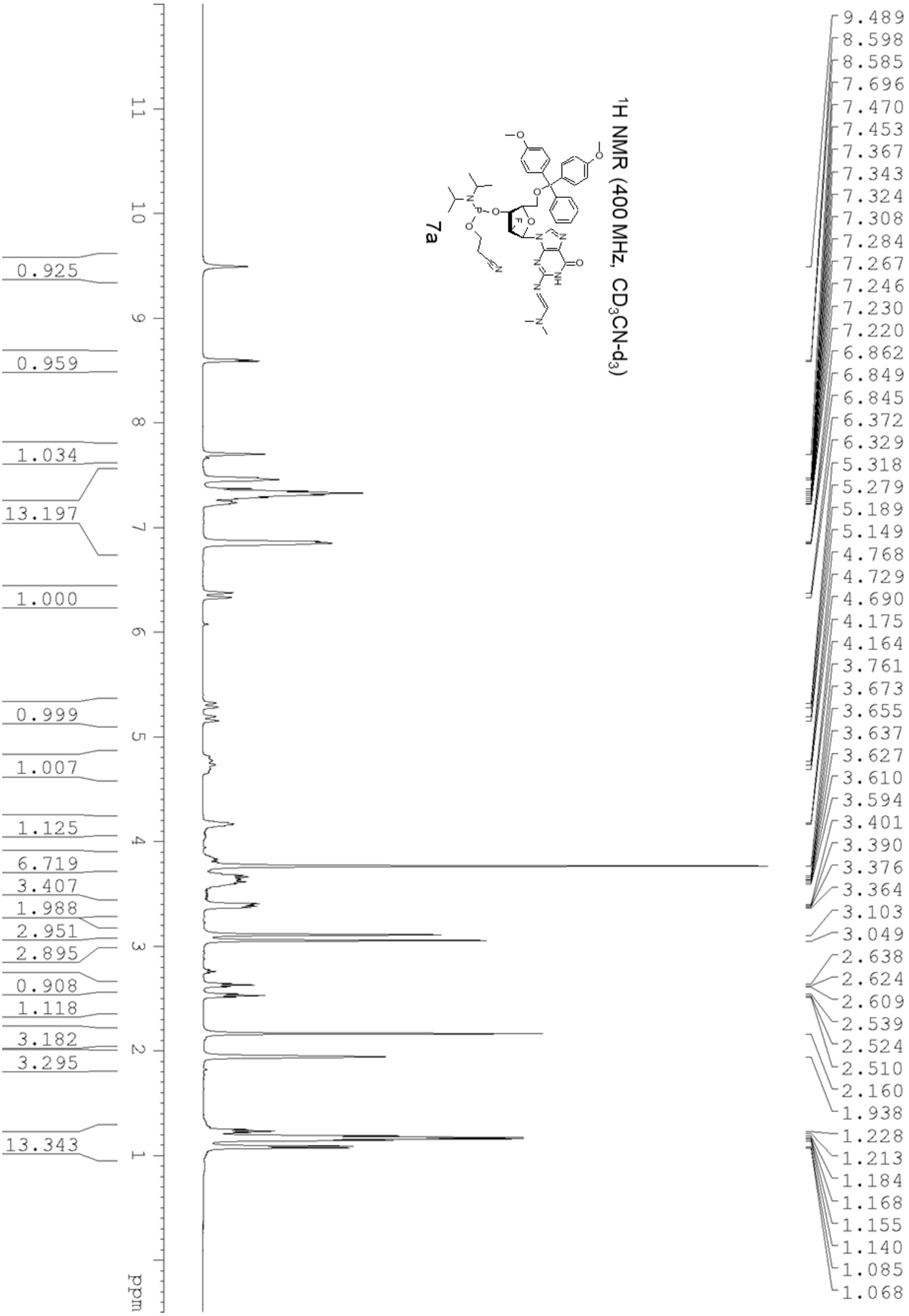

1H NMR (400 MHz, CD3CN-d3)
7a
9.489
8.598
8.585
7.696
7.470
7.453
7.367
7.343
7.324
7.308
7.284
7.267
7.246
7.230
7.220
6.862
6.849
6.845
6.372
6.329
5.318
5.279
5.189
5.149
4.768
4.729
4.690
4.175
4.164
3.761
3.673
3.655
3.637
3.627
3.610
3.594
3.401
3.390
3.376
3.364
3.103
3.049
2.638
2.624
2.609
2.539
2.524
2.510
2.160
1.938
1.228
1.213
1.184
1.168
1.155
1.140
1.085
1.068
0.925
0.959
1.034
13.197
1.000
0.999
1.007
1.125
6.719
3.407
1.988
2.951
2.895
0.908
1.118
3.182
3.295
13.343
ppm

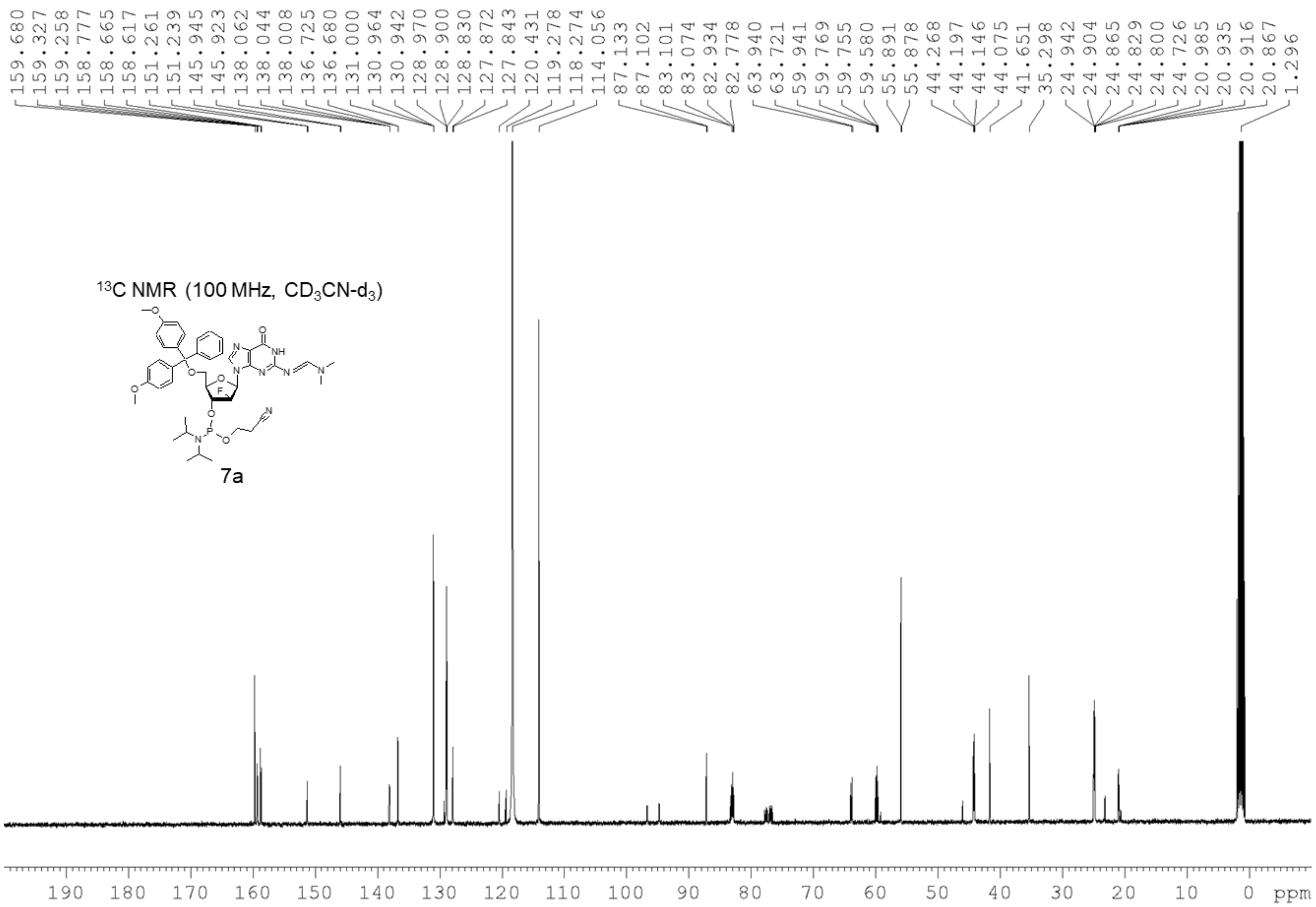

¹³C NMR (100 MHz, CD₃CN-d₃)
7a
159.680
159.327
159.258
158.777
158.665
158.617
151.261
151.239
145.945
145.923
138.062
138.044
138.008
136.725
136.680
131.000
130.964
130.942
128.970
128.900
128.830
127.872
127.843
120.431
119.278
118.274
114.056
87.133
87.102
83.101
83.074
82.934
82.778
63.940
63.721
59.941
59.769
59.755
59.580
55.891
55.878
44.268
44.197
44.146
44.075
41.651
35.298
24.942
24.904
24.865
24.829
24.800
24.726
20.985
20.935
20.916
20.867
1.296
190 180 170 160 150 140 130 120 110 100 90 80 70 60 50 40 30 20 10 0 ppm

^{19}F NMR (470 MHz, CD_3CN-d_3)

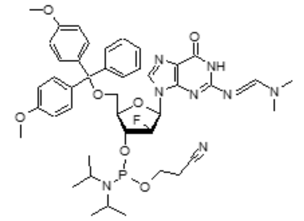

7a

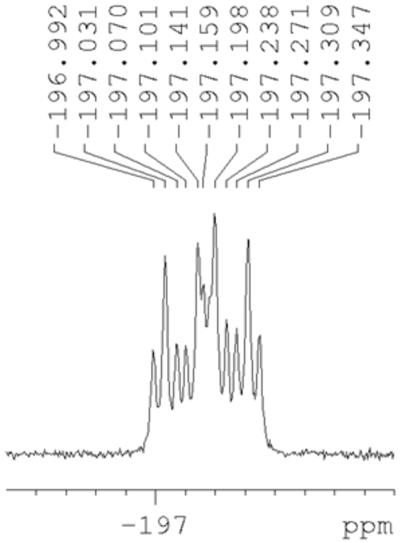

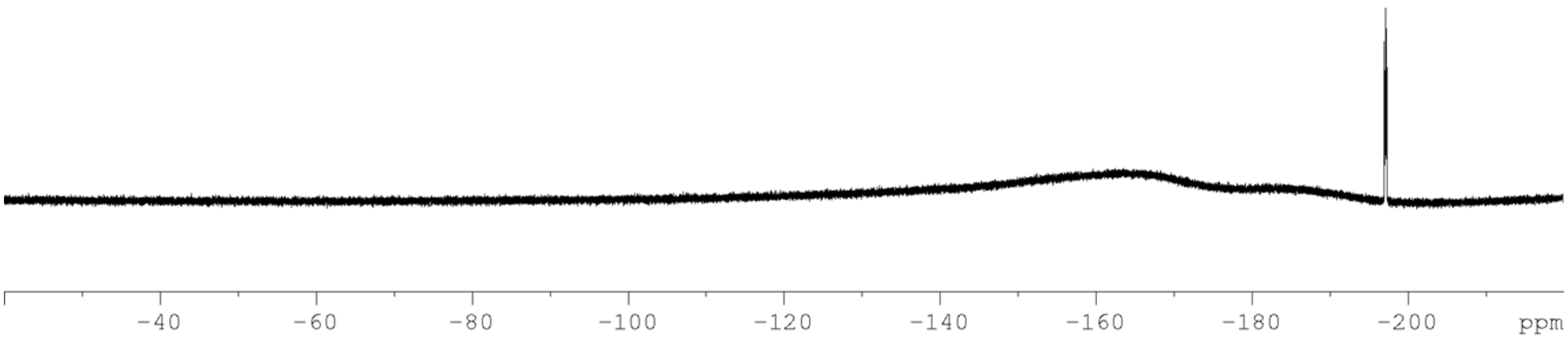

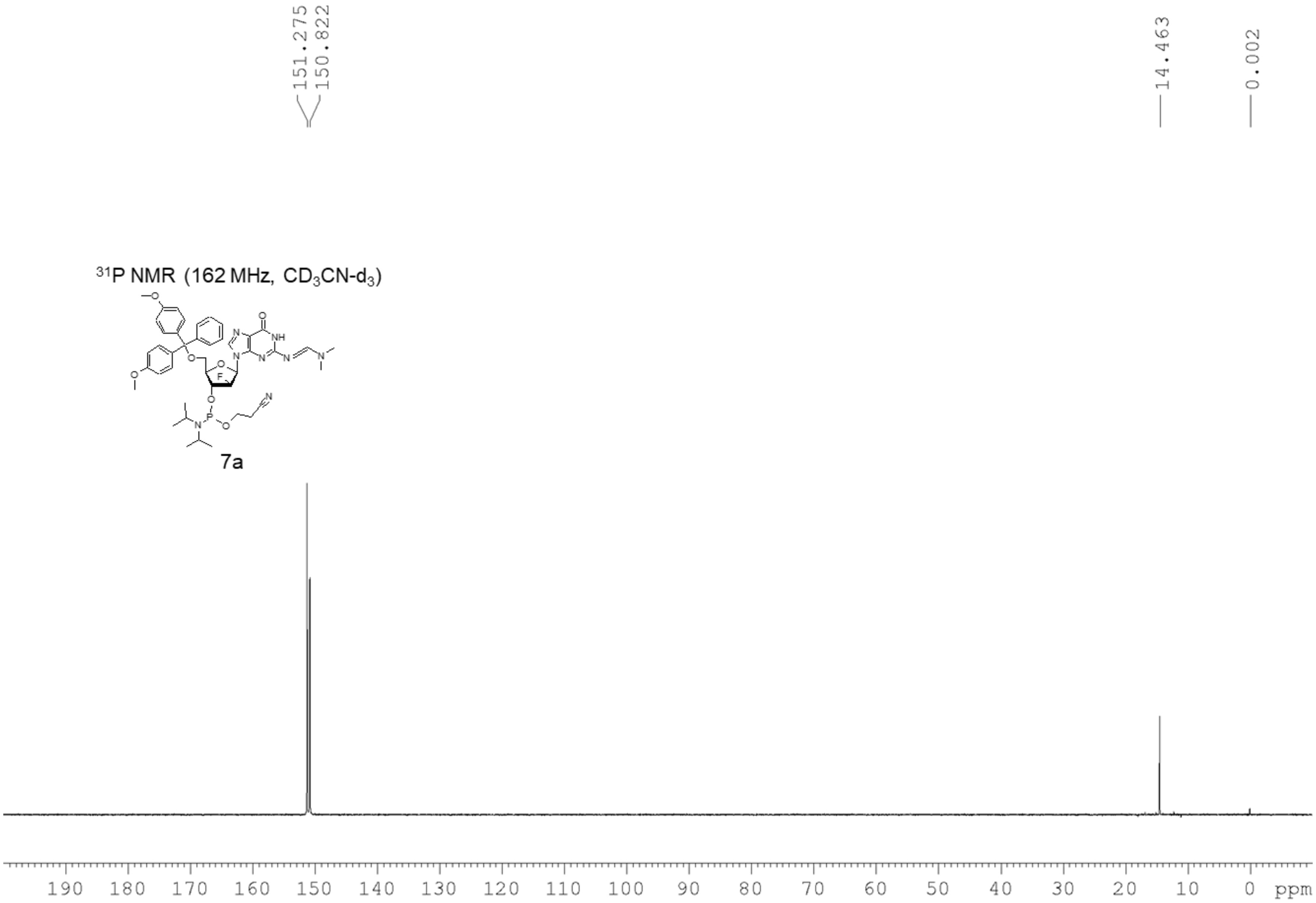
^{31}P NMR (162 MHz, CD$_3$CN-d$_3$)
7a
151.275
150.822
14.463
0.002
190 180 170 160 150 140 130 120 110 100 90 80 70 60 50 40 30 20 10 0 ppm

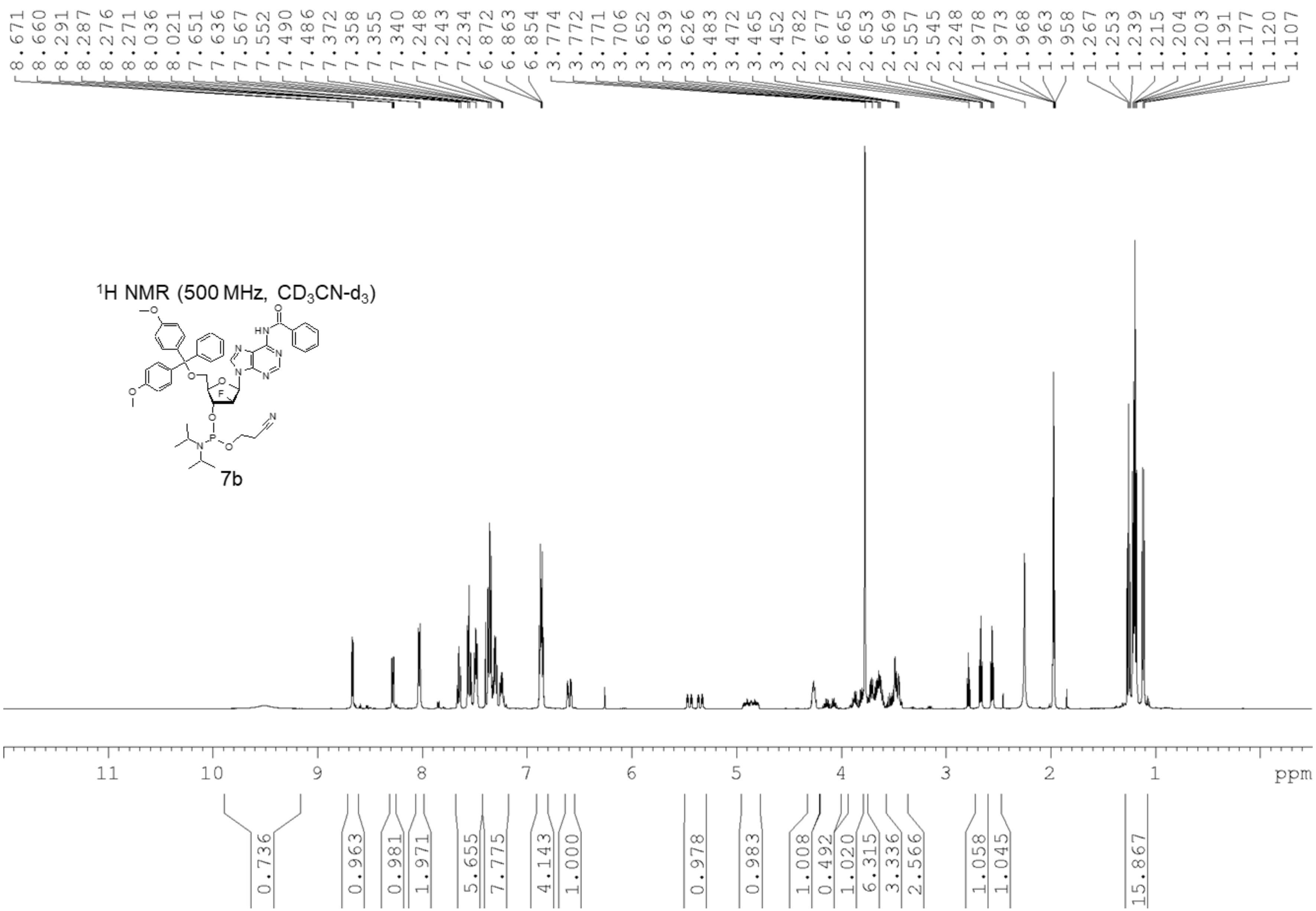

1H NMR (500 MHz, CD3CN-d3)
7b
8.671
8.660
8.291
8.287
8.276
8.271
8.036
8.021
7.651
7.636
7.567
7.552
7.490
7.486
7.372
7.358
7.355
7.340
7.248
7.243
7.234
6.872
6.863
6.854
3.774
3.772
3.771
3.706
3.652
3.639
3.626
3.483
3.472
3.465
3.452
2.782
2.677
2.665
2.653
2.569
2.557
2.545
2.248
1.978
1.973
1.968
1.963
1.958
1.267
1.253
1.239
1.215
1.204
1.203
1.191
1.177
1.120
1.107
11
10
9
8
7
6
5
4
3
2
1
ppm
0.736
0.963
0.981
1.971
5.655
7.775
4.143
1.000
0.978
0.983
1.008
0.492
1.020
6.315
3.336
2.566
1.058
1.045
15.867

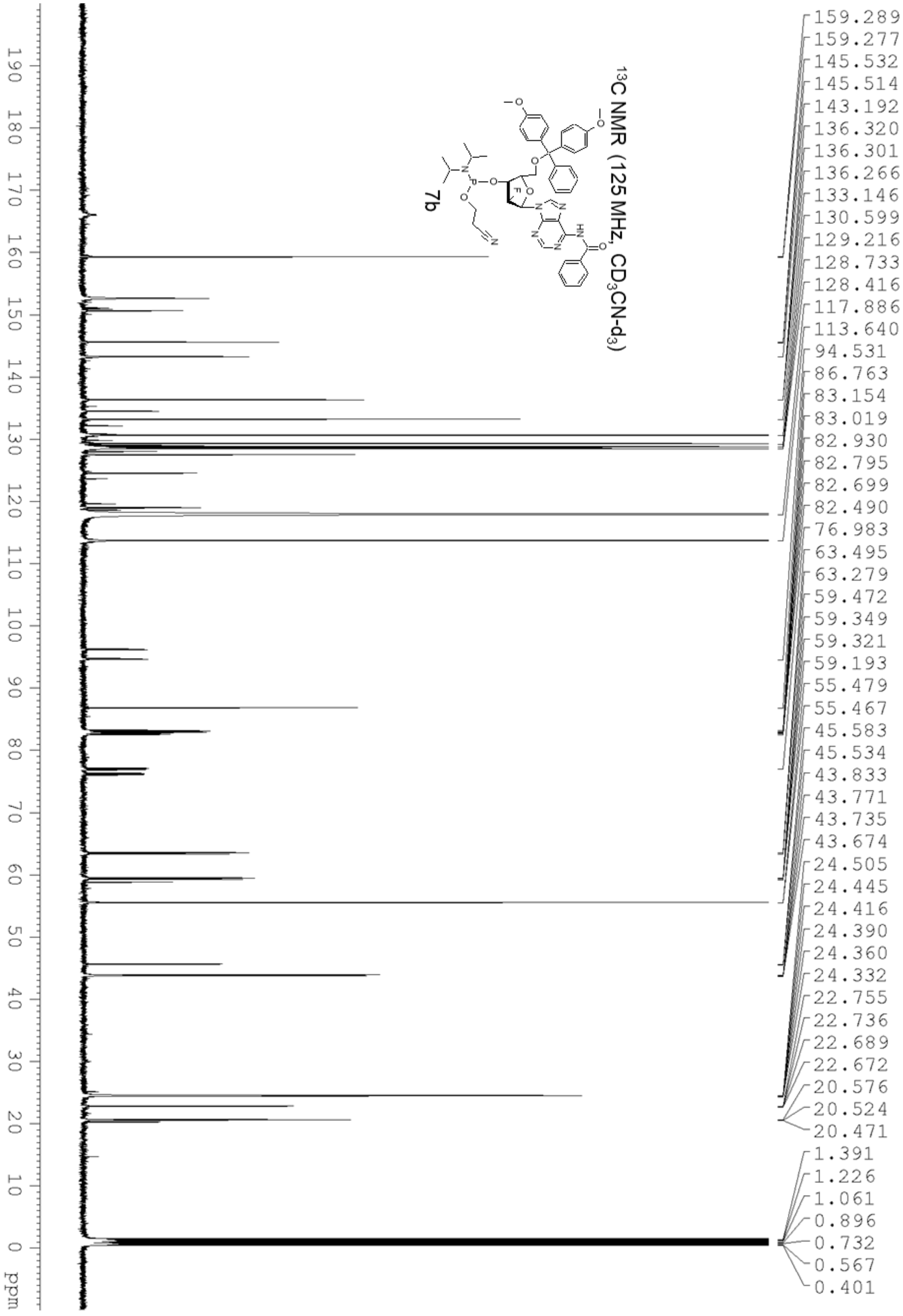

13C NMR (125 MHz, CD3CN-d3)
7b
159.289
159.277
145.532
145.514
143.192
136.320
136.301
136.266
133.146
130.599
129.216
128.733
128.416
117.886
113.640
94.531
86.763
83.154
83.019
82.930
82.795
82.699
82.490
76.983
63.495
63.279
59.472
59.349
59.321
59.193
55.479
55.467
45.583
45.534
43.833
43.771
43.735
43.674
24.505
24.445
24.416
24.390
24.360
24.332
22.755
22.736
22.689
22.672
20.576
20.524
20.471
1.391
1.226
1.061
0.896
0.732
0.567
0.401
ppm

^{19}F NMR (470 MHz, CD_3CN-d_3)

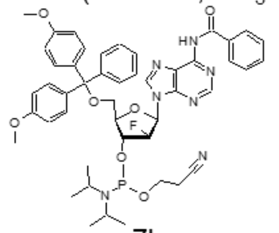

7b

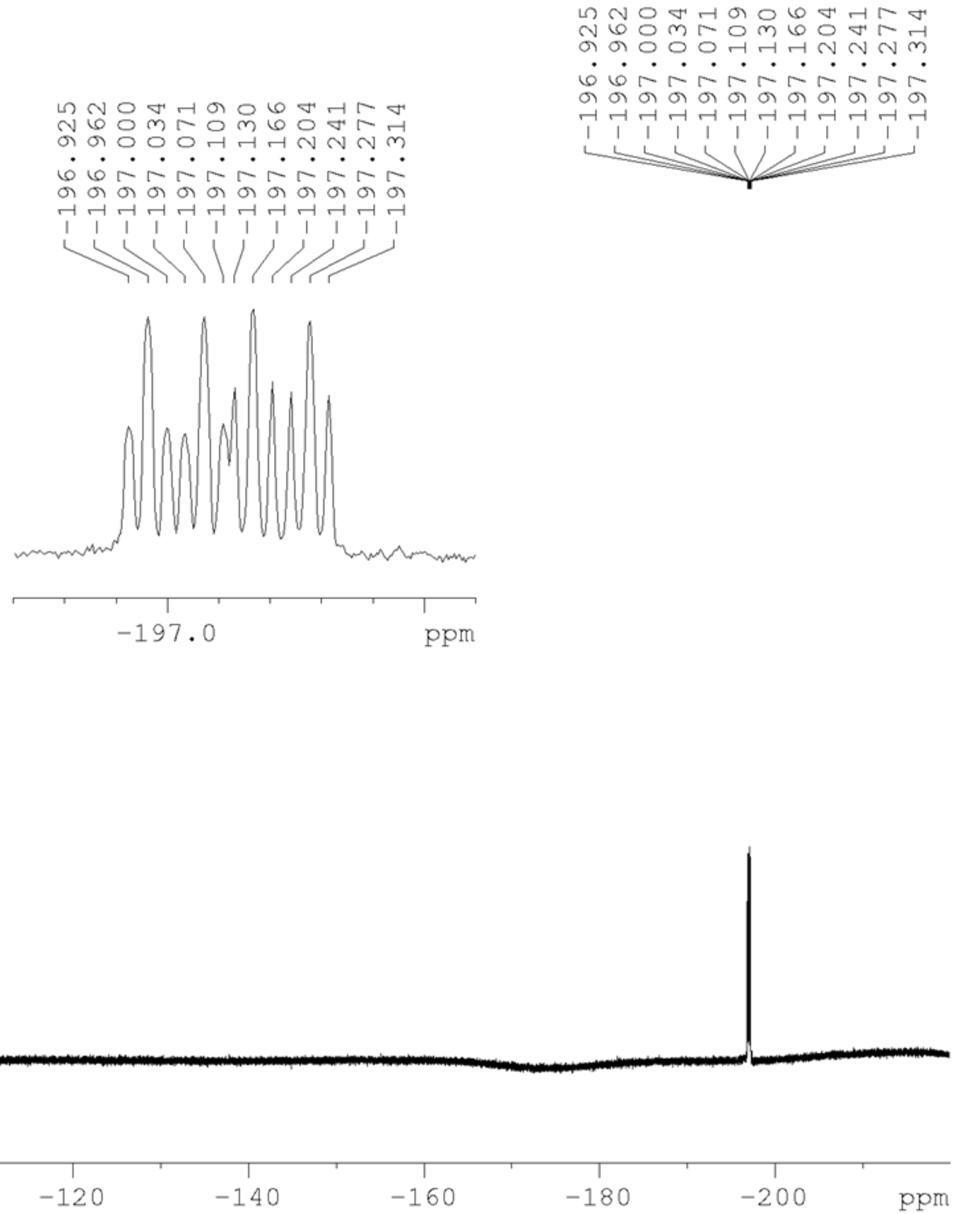

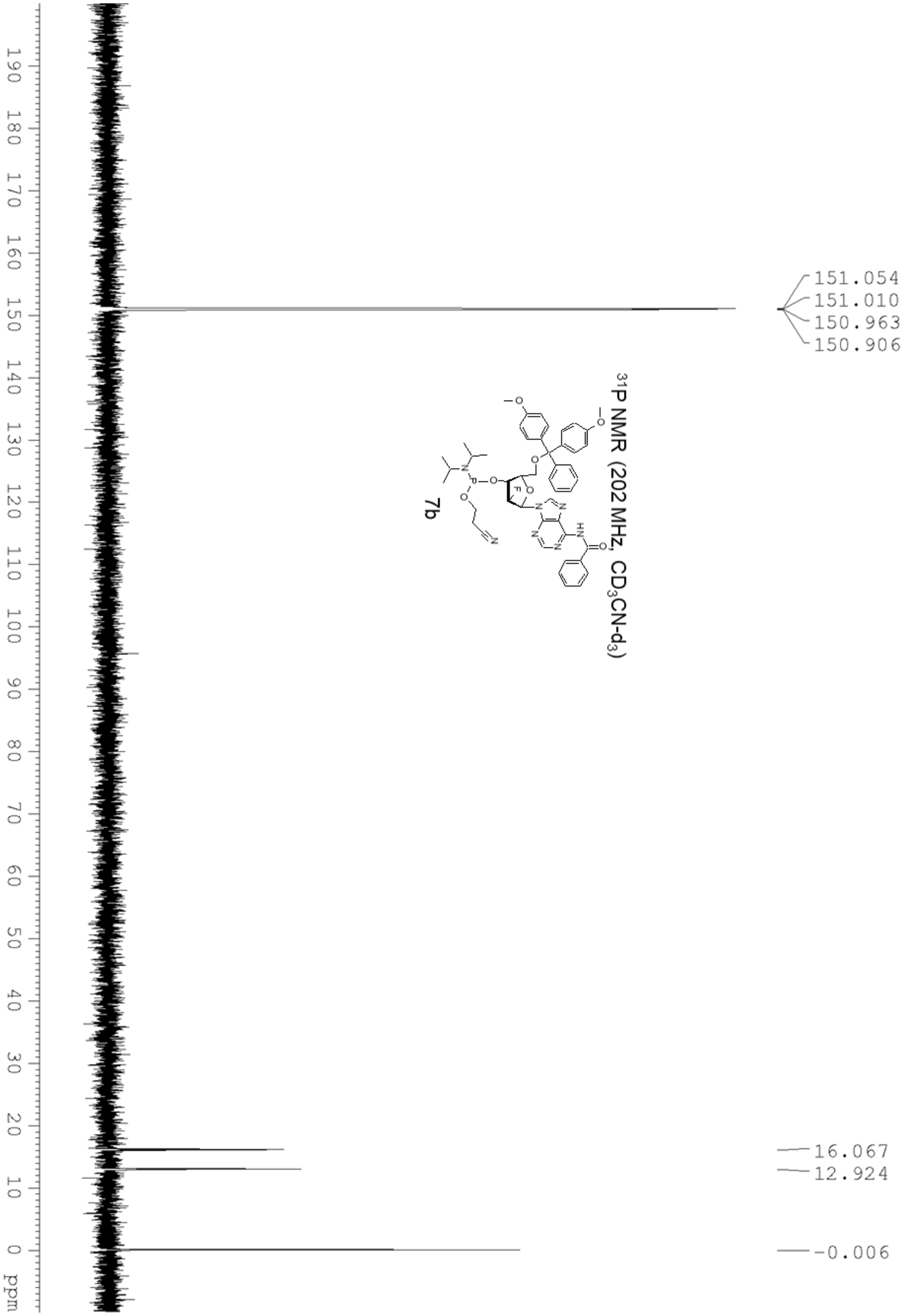

^{31}P NMR (202 MHz, CD_3CN-d_3)
7b
151.054
151.010
150.963
150.906
16.067
12.924
-0.006
190
180
170
160
150
140
130
120
110
100
90
80
70
60
50
40
30
20
10
0
ppm

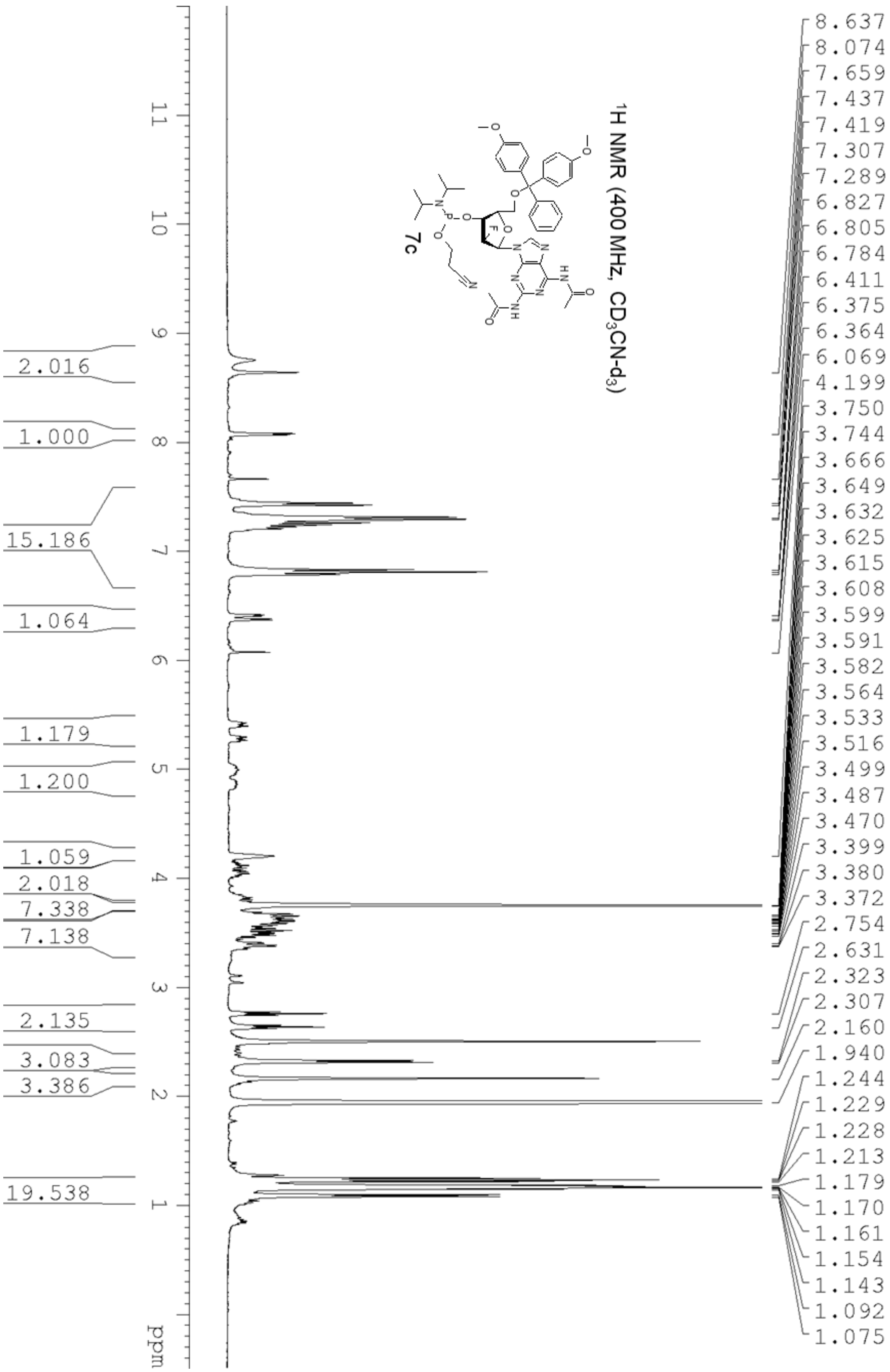

^{1}H NMR (400 MHz, CD$_3$CN-d$_3$)
7c
8.637
8.074
7.659
7.437
7.419
7.307
7.289
6.827
6.805
6.784
6.411
6.375
6.364
6.069
4.199
3.750
3.744
3.666
3.649
3.632
3.625
3.615
3.608
3.599
3.591
3.582
3.564
3.533
3.516
3.499
3.487
3.470
3.399
3.380
3.372
2.754
2.631
2.323
2.307
2.160
1.940
1.244
1.229
1.228
1.213
1.179
1.170
1.161
1.154
1.143
1.092
1.075
2.016
1.000
15.186
1.064
1.179
1.200
1.059
2.018
7.338
7.138
2.135
3.083
3.386
19.538
11
10
9
8
7
6
5
4
3
2
1
ppm

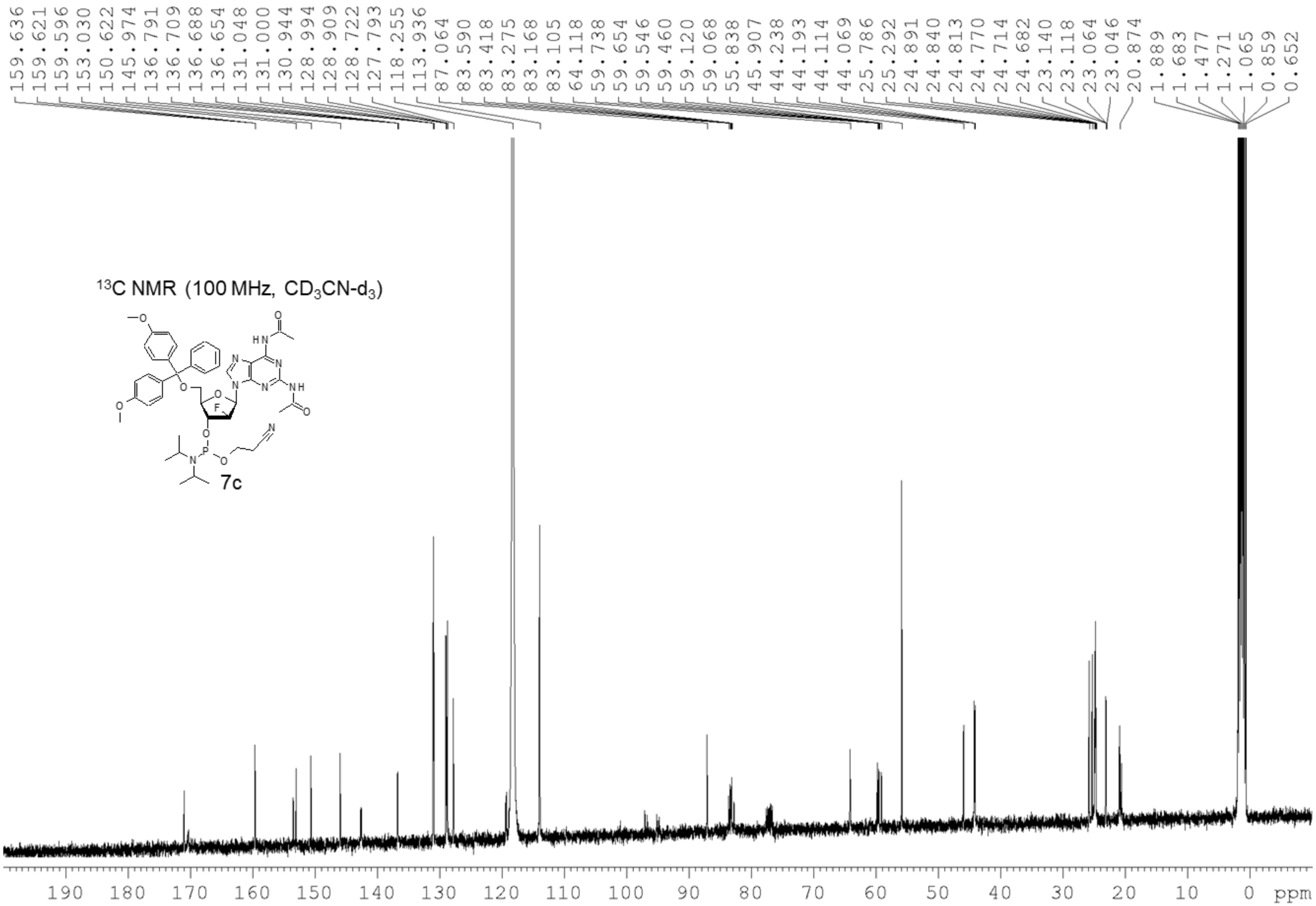

^{13}C NMR (100 MHz, CD$_3$CN-d$_3$)
7c
159.636
159.621
159.596
153.030
150.622
145.974
136.791
136.709
136.688
136.654
131.048
131.000
130.944
128.994
128.909
128.722
127.793
118.255
113.936
87.064
83.590
83.418
83.275
83.168
83.105
64.118
59.738
59.654
59.546
59.460
59.120
59.068
55.838
45.907
44.238
44.193
44.114
44.069
25.786
25.292
24.891
24.840
24.813
24.770
24.714
24.682
23.140
23.118
23.064
23.046
20.874
1.889
1.683
1.477
1.271
1.065
0.859
0.652
190 180 170 160 150 140 130 120 110 100 90 80 70 60 50 40 30 20 10 0 ppm

^{19}F NMR (376 MHz, CD_3CN-d_3)

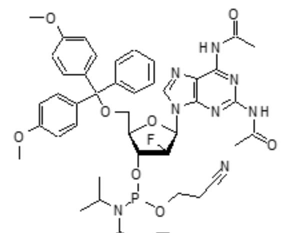

7c

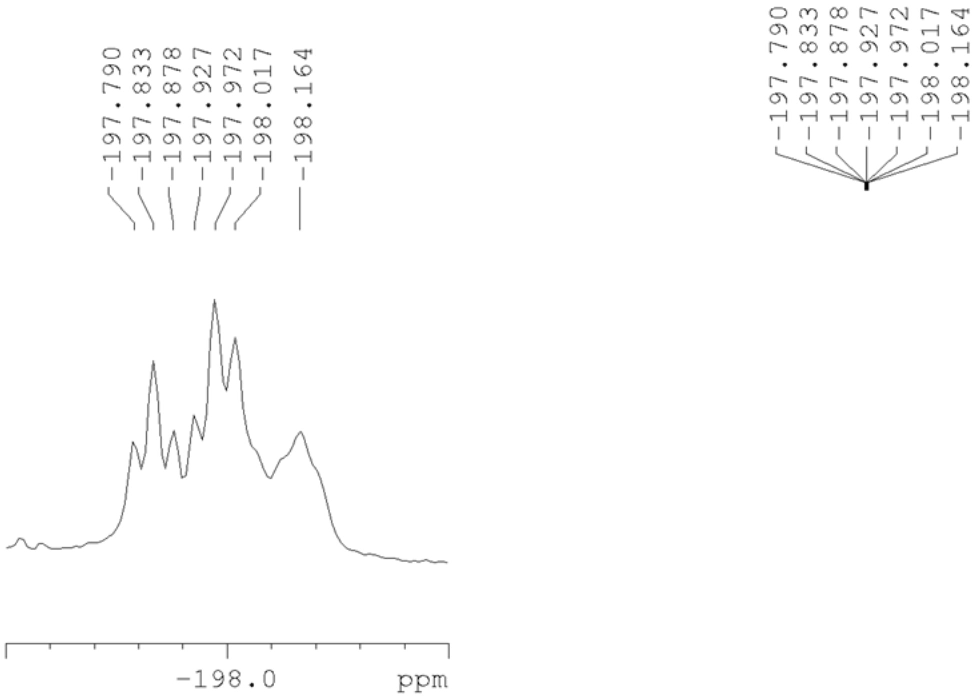

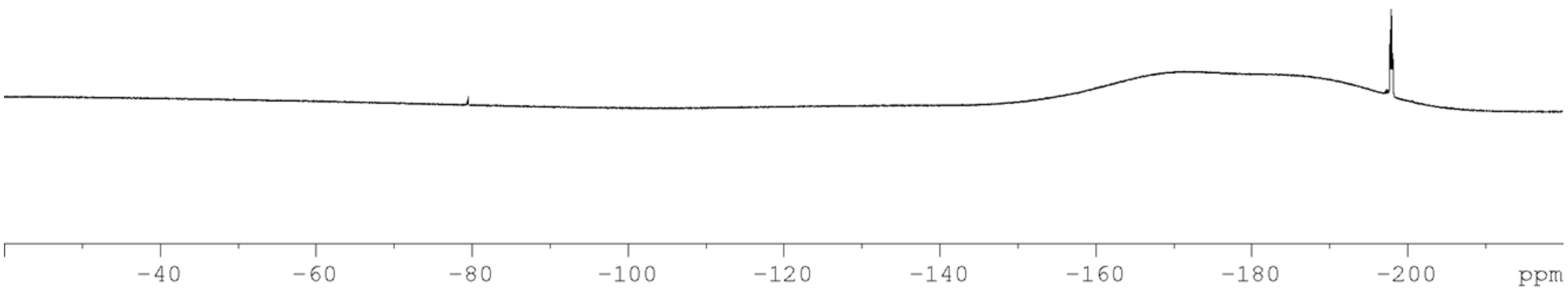

^{31}P NMR (162 MHz, CD_3CN-d_3)

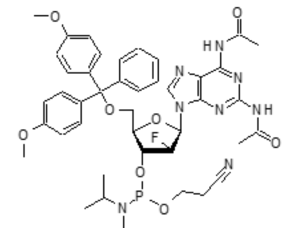

7c

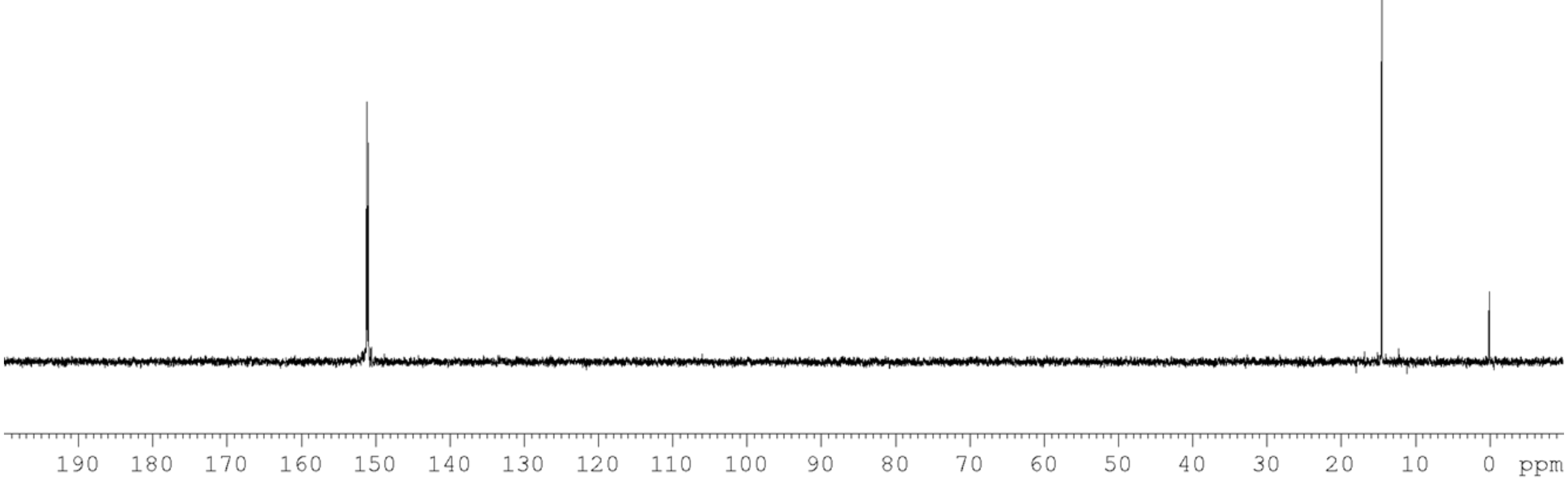

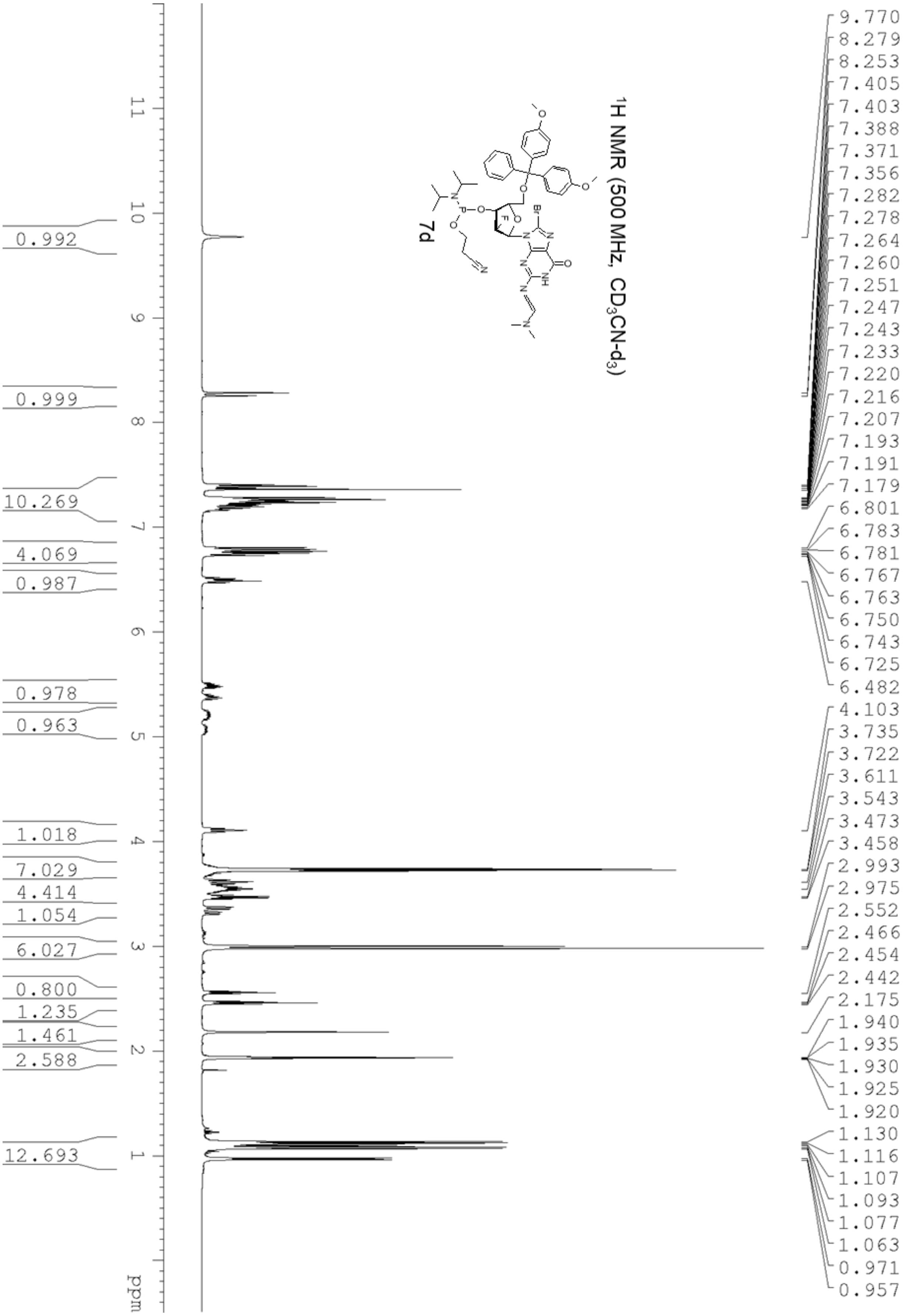

1H NMR (500 MHz, CD3CN-d3)
7d

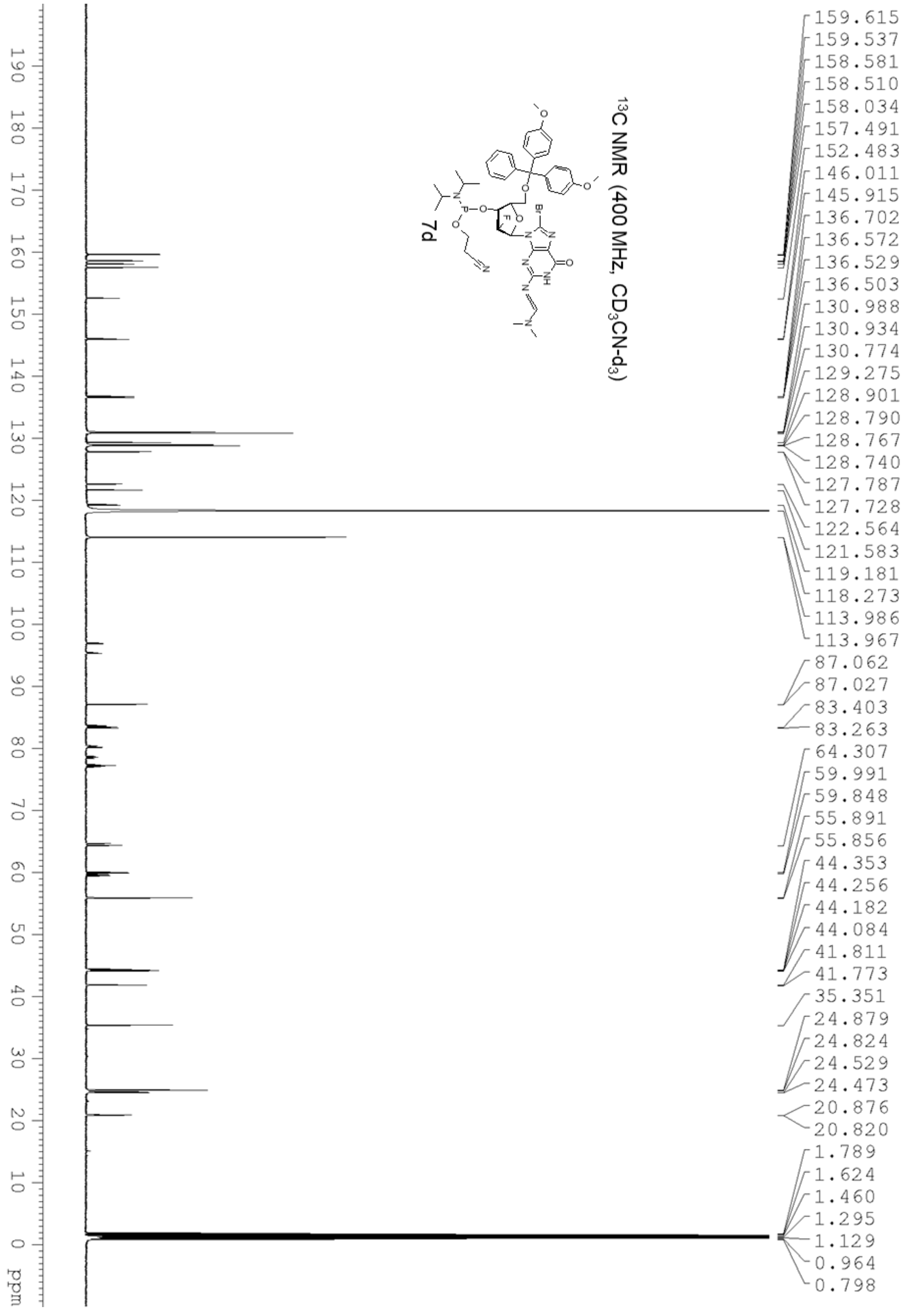
13C NMR (400 MHz, CD3CN-d3)
7d
159.615
159.537
158.581
158.510
158.034
157.491
152.483
146.011
145.915
136.702
136.572
136.529
136.503
130.988
130.934
130.774
129.275
128.901
128.790
128.767
128.740
127.787
127.728
122.564
121.583
119.181
118.273
113.986
113.967
87.062
87.027
83.403
83.263
64.307
59.991
59.848
55.891
55.856
44.353
44.256
44.182
44.084
41.811
41.773
35.351
24.879
24.824
24.529
24.473
20.876
20.820
1.789
1.624
1.460
1.295
1.129
0.964
0.798
ppm

^{19}F NMR (470 MHz, CD_3CN-d_3)

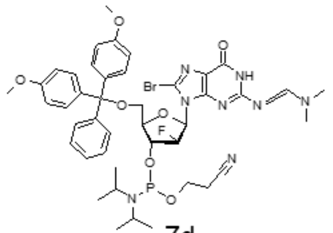

7d

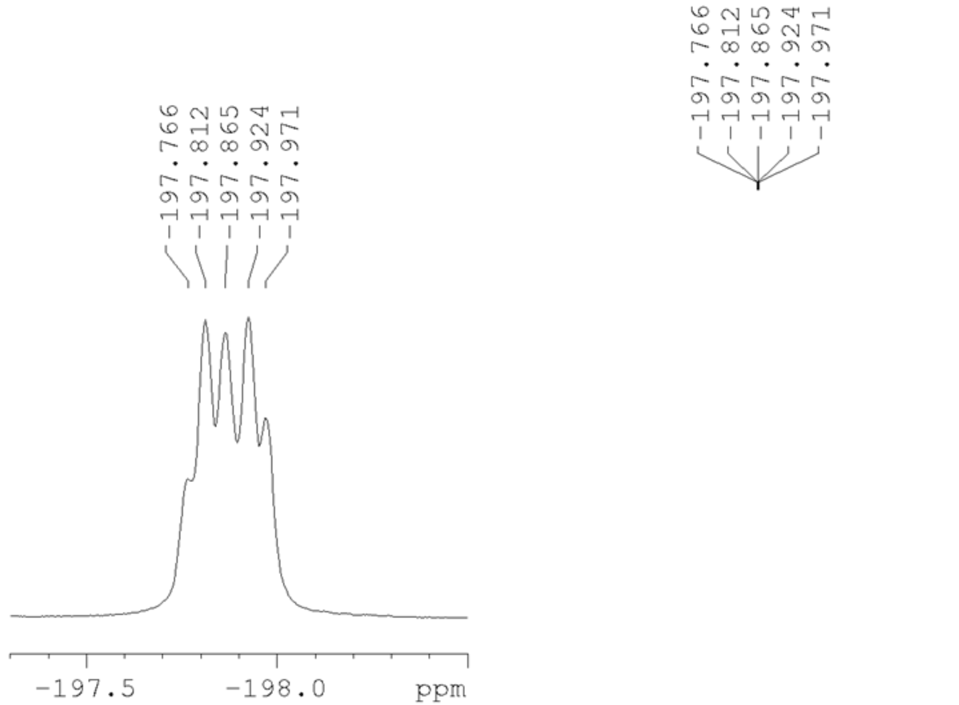

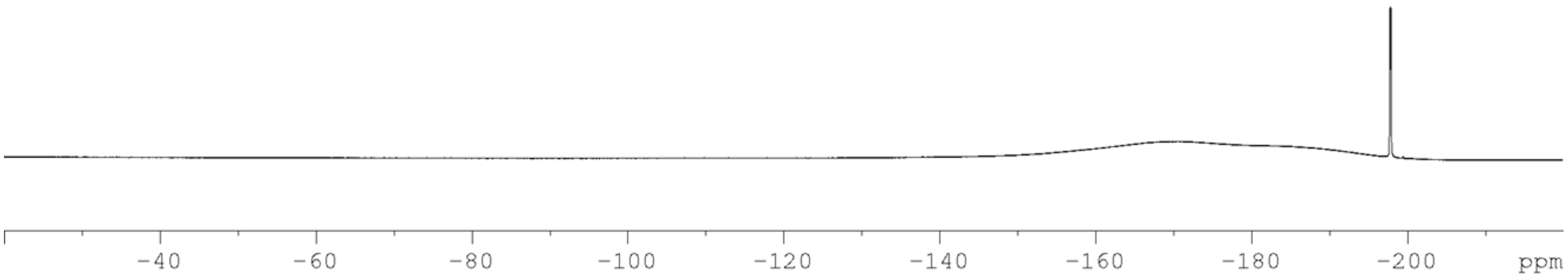

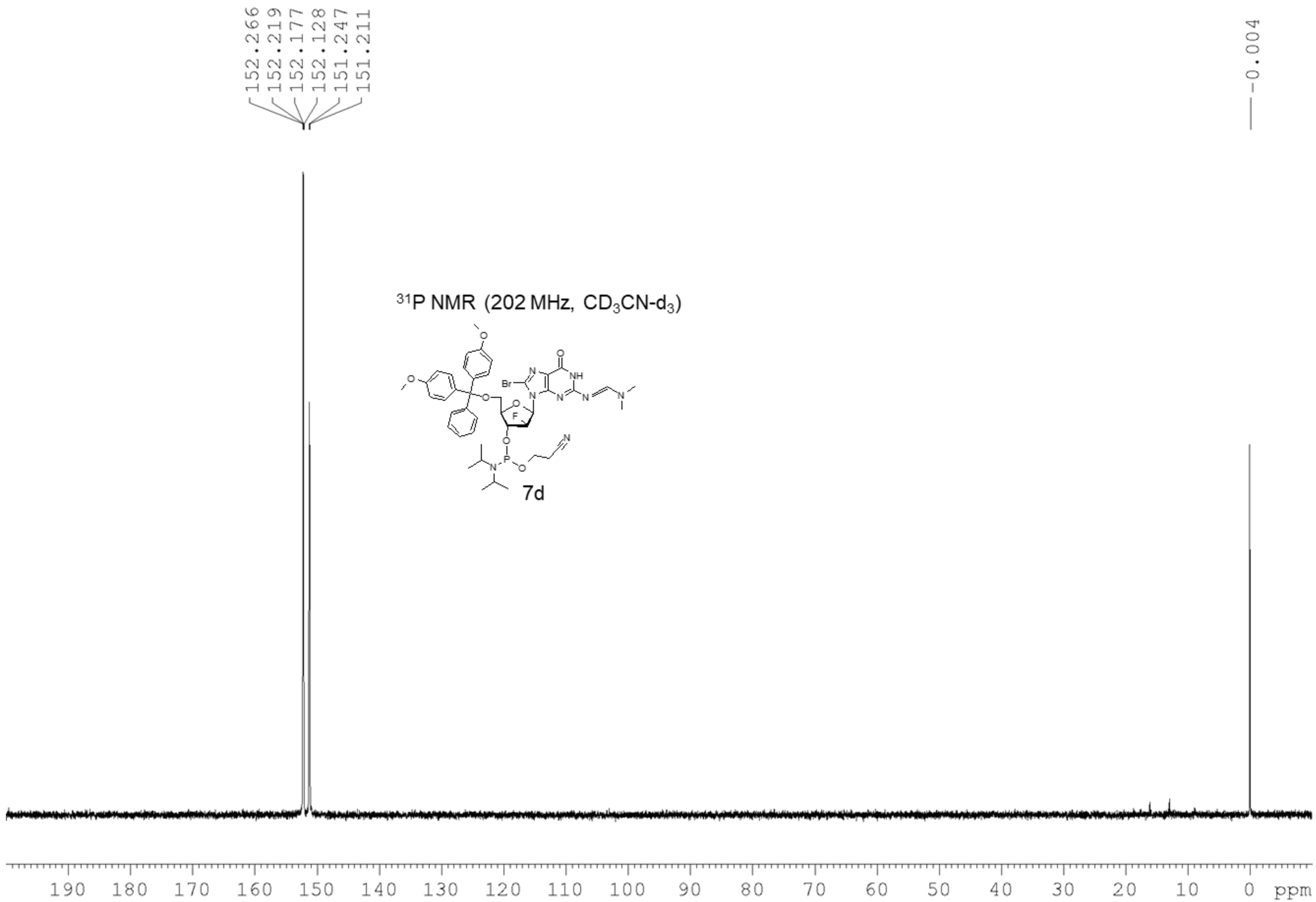
152.266
152.219
152.177
152.128
151.247
151.211
-0.004
^{31}P NMR (202 MHz, CD_3CN-d_3)
7d
190 180 170 160 150 140 130 120 110 100 90 80 70 60 50 40 30 20 10 0 ppm

S5. Polyacrylamide gels (kinetic assay).

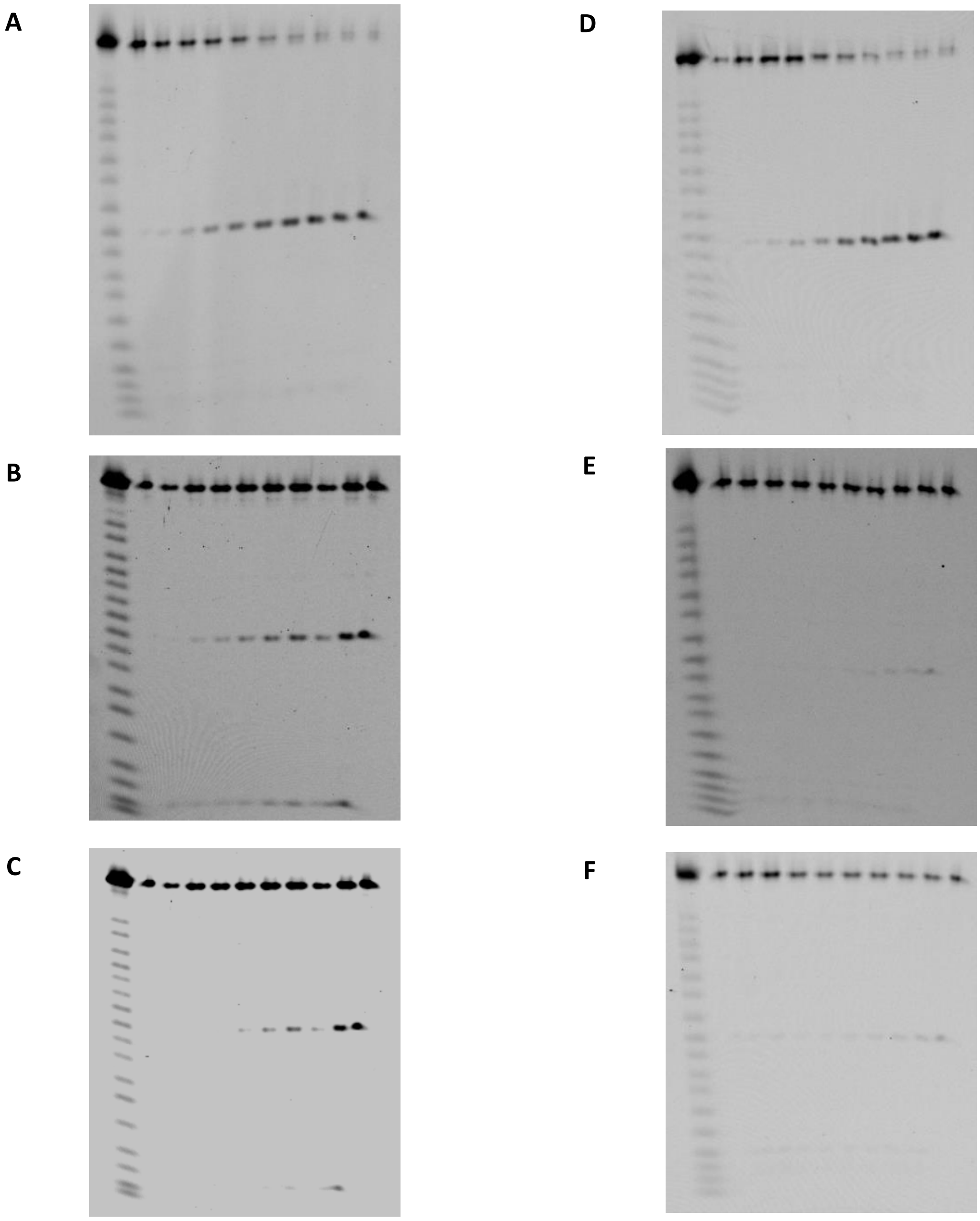

Figure S5. Examples of 12% polyacrylamide denaturing gels electrophoresis of chosen ribozymes kinetic assay A – RH1, B – RZ6A, C – RZ6C, D – RH1-12-5, E – RH1-8-5, F – RZ6-12-8 (Table 3). Far-left lane shows the Mg-ladder of FAM-labeled target RNA oligonucleotide. From left to right 0, 0.5, 1, 2.5, 5, 15, 30, 45, 60 and 90 min quenching time of respective ribozyme reaction. The observed cleavage is as expected after the NUH sequence in the FAM-labeled target oligoribonucleotide (Figure 1), giving a visible band of 10nt product.

S6. Reaction rate plots.

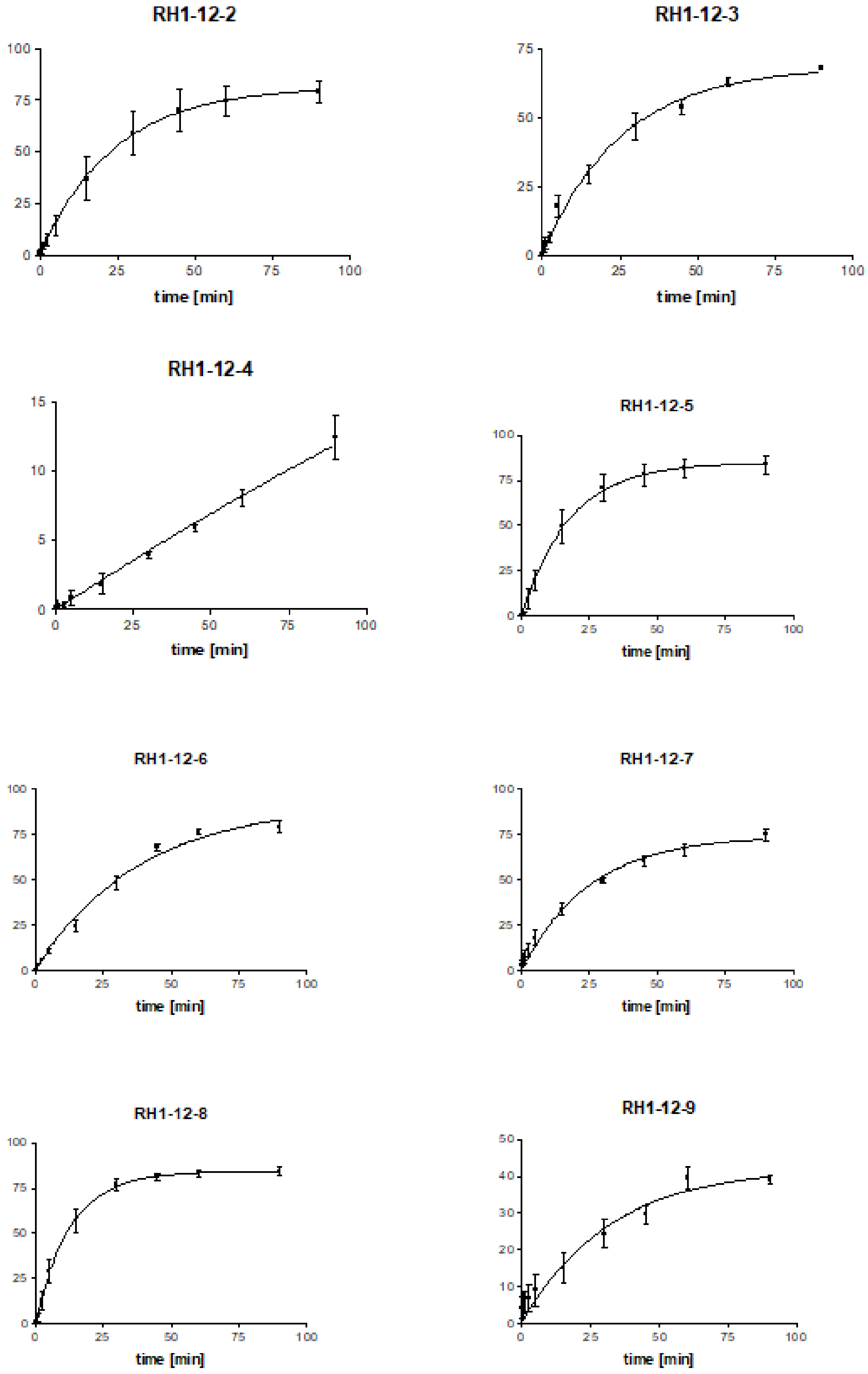

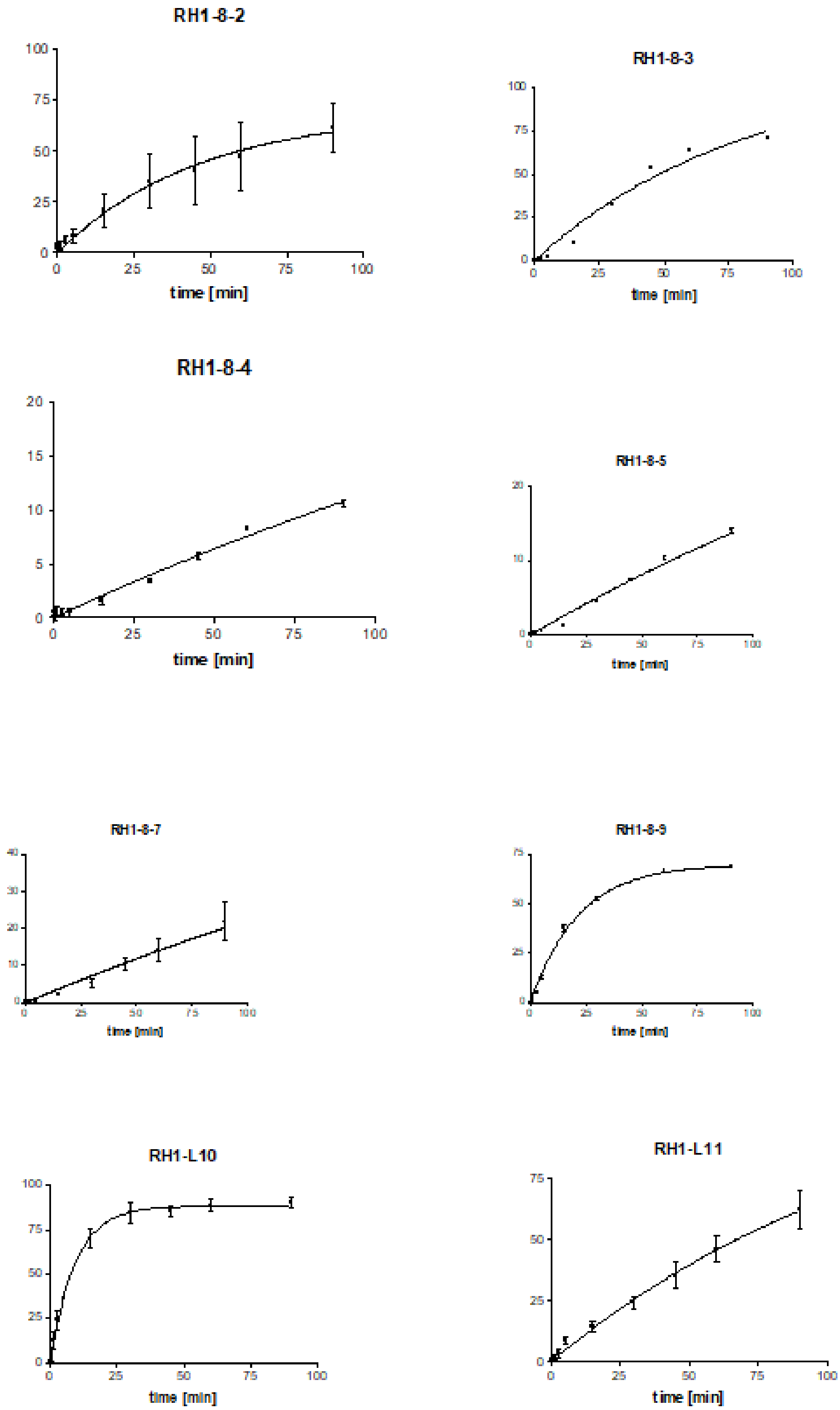

Figure S6. Kinetic curves of tested ribozymes. The bands from PAGE (Figure S5) were quantified, and k_{obs} parameters were obtained by fitting to $f = 1-e^{-kt}$ formula and collected in Table 4.

S7. Suplemental references.

1. Piasecka, J., Lenartowicz, E., Soszynska-Jozwiak, M., Szutkowska, B., Kierzek, R., and Kierzek, E. (2020). RNA secondary structure motifs of the influenza A virus as targets for siRNA-mediated RNA interference. Molecular Therapy - Nucleic Acids *19*, 627–642.
